# An Evaluation of Different Target Enrichment Methods in Pooled Sequencing Designs for Complex Disease Association Studies

**DOI:** 10.1371/journal.pone.0026279

**Published:** 2011-11-01

**Authors:** Aaron G. Day-Williams, Kirsten McLay, Eleanor Drury, Sarah Edkins, Alison J. Coffey, Aarno Palotie, Eleftheria Zeggini

**Affiliations:** 1 Wellcome Trust Sanger Institute, Wellcome Trust Genome Campus, Hinxton, Cambridgeshire, United Kingdom; 2 The Genome Analysis Centre, Norwich, United Kingdom; 3 Institute for Molecular Medicine Finland (FIMM), University of Helsinki, Helsinki, Finland; 4 Program in Medical and Population Genetics and Genetic Analysis Platform, The Broad Institute of MIT and Harvard, Cambridge, Massachusetts, United States of America; 5 Department of Medical Genetics, University of Helsinki and University Central Hospital, Helsinki, Finland; The University of Hong Kong, Hong Kong

## Abstract

Pooled sequencing can be a cost-effective approach to disease variant discovery, but its applicability in association studies remains unclear. We compare sequence enrichment methods coupled to next-generation sequencing in non-indexed pools of 1, 2, 10, 20 and 50 individuals and assess their ability to discover variants and to estimate their allele frequencies. We find that pooled resequencing is most usefully applied as a variant discovery tool due to limitations in estimating allele frequency with high enough accuracy for association studies, and that in-solution hybrid-capture performs best among the enrichment methods examined regardless of pool size.

## Introduction

Genome-wide association studies (GWAS) have precipitated a dramatic rise in the discovery of novel, robustly-associated complex trait loci. As the majority of these signals involve common alleles with modest or small effect sizes, a large proportion of genetic variance remains unexplained. Low frequency (minor allele frequency [MAF]
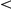
0.05) and rare (MAF
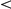
0.01) variants may be associated with complex traits and help account for the ‘missing’ heritability [1], [2] (for example as recently shown for hypertriglyceridemia [3]). A comprehensive catalogue of rare variants does not yet exist, although large-scale resequencing efforts such as the 1000 Genomes (1KG) [4] and UK10K (www.uk10k.org) Projects are enhancing our understanding of human sequence variation.

Experimental costs associated with variant discovery have been drastically reduced through the advent of next-generation sequencing technologies; however, whole-genome deep sequencing of individual samples in large disease association studies remains prohibitively expensive and likely will for some time. Pooling DNA samples could empower cost-efficient sequence variant identification and allele frequency estimation. This can in theory enable comparisons between disease cases and controls, bypassing the need for exhaustive genotyping, and allowing the identification of promising novel association signals, for example as applied to the discovery of the type 1 diabetes IFIH1 locus [5].

Non-indexed, or non-barcoded, pools (which form the focus of this study) do not enable the assignment of variants to individuals, but have lower associated costs. Even though targeted resequencing in pools has attractive attributes that may facilitate disease association studies, technical and analytical parameters central to this study design have not been empirically evaluated yet. Pooling studies are sensitive to DNA quantification and pool construction. The choice of target enrichment method is important. PCR is difficult to multiplex, optimize and normalize, but can be highly effective. The ability of PCR-enriched targeted resequencing to sensitively identify low frequency and rare variants and estimate their frequency in non-indexed pools has been established, but evaluations have been restricted to small-scale experiments investigating up to 300 kb [5]–[12]; however, most post-GWAS sequencing efforts target several megabases. Hybrid-capture methods (array-based [aHC] and in-solution hybrid-capture [sHC]) are easy to multiplex and enable large-scale experiments [13]–[17]. A recent investigation illustrated that they can be successfully applied to the targeted resequencing of 2.6 Mb in individual samples [18], but their effectiveness in pooled samples is not clear.

Here, we assess variant detection and frequency estimation of different sequence enrichment methods (long-range PCR, aHC and sHC) in non-indexed pools of 1, 2, 10, 20 and 50 samples (Tables S1, S2) across six genomic regions encompassing coding and non-coding sequence (1.6 Mb in total, Table S3), and evaluate the feasibility of these approaches in the context of complex disease association studies. Specifically, we evaluate the uniformity of target coverage, the sensitivity and specificity of variant detection and the accuracy of frequency estimation in non-indexed pools of different sizes and across different enrichment methods for the first time.

## Results

### Alignment of Reads to Target

Enrichment specificity can be assessed by comparing the proportion of sequencing reads that map to the target regions. The lower the specificity, the higher the sequencing capacity required to achieve the desired target coverage. We observed large variability in the total number of reads produced by each of the three enrichment methods (Table 1, Table S4). This variability is also evident for the PCR and aHC technical replicates we conducted (for the Pool of 20; Tables S5, S6). It is common practice in whole genome sequencing (WGS) to remove potential duplicate reads to avoid biases in coverage analyses as well as downstream analyses, but applying this practice in pooled targeted sequencing of a relatively small target region with a high depth of coverage is still a matter of debate. Therefore we calculated alignment statistics both before and after removing potential duplicate reads. PCR showed the highest percentage of sequencing reads that map to the target region both before and after duplicate read removal (Table 1, Table S4). Conversely, both aHC and sHC showed higher proportions of mapped on-target reads with good mapping quality scores (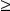
20) both before and after duplicate read removal (Table 1, Table S4). The mapping quality score of reads is an important factor in accurate variant detection and the specificity of target enrichment impacts directly on target coverage.

**Table 1:** Target sequence enrichment success before duplicate removal.

Pool	Number	Total Number	% Reads Mapped	% Reads Mapped	% Reads Mapped
of	Lanes	Reads	to Reference^a^	to Target^a^	to Target w/ 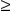 Q20^b^
1 PCR	1	44,232,852	48.97	46.05	44.27
1 aPD	1	61,487,334	95.80	21.82	21.58
1 sPD	1	35,813,898	97.90	46.55	45.95
2 PCR	1	30,843,770	97.92	85.97	79.61
2 aPD	1	58,352,664	92.19	13.07	12.91
2 sPD	1	29,554,192	97.50	46.96	46.36
10 PCR	2	55,278,922	84.51	73.44	67.02
10 aPD	2	90,319,688	96.44	18.62	18.15
10 sPD	2	85,783,964	97.83	48.13	47.48
20 PCR	3	121,378,560	89.33	80.88	75.37
20 aPD	3	103,231,280	97.24	34.05	33.44
20 sPD	3	111,444,476	97.11	45.91	45.31
50 PCR	7	132,547,082	99.74	70.90	67.42
50 aPD	7	251,257,124	96.02	22.62	22.27
50 sPD	7	295,115,044	97.52	49.97	49.30

For each pool and sequence enrichment method this table details the total number of reads generated for the pool, the percentage of total reads mapped to the reference genome, the percentage of total reads mapped to the target regions, and the percentage of mapped reads that mapped to the target regions with mapping quality 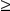
20. The total number of reads for a pool is calculated from the fastq file(s) generated for each lane of sequencing. The percentage of reads mapped to the reference is calculated from the BAM file generated from merging all the Maq map files for each lane for a pool. The percentage of reads mapped to the target regions is calculated as the number of reads with at least one base overlapping a target region divided by the total number of reads. The percentage of reads mapped to the target regions with a mapping quality score 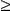
Q20 is calculated as the number of reads with at least one base overlapping a target region with mapping Q
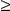
20 divided by the total number of reads.

a : Calculated by samtools view –c.

b : Calculated by samtoools veiw -c -q 20.

### Target Coverage Depth and Uniformity

**Figure 1:**
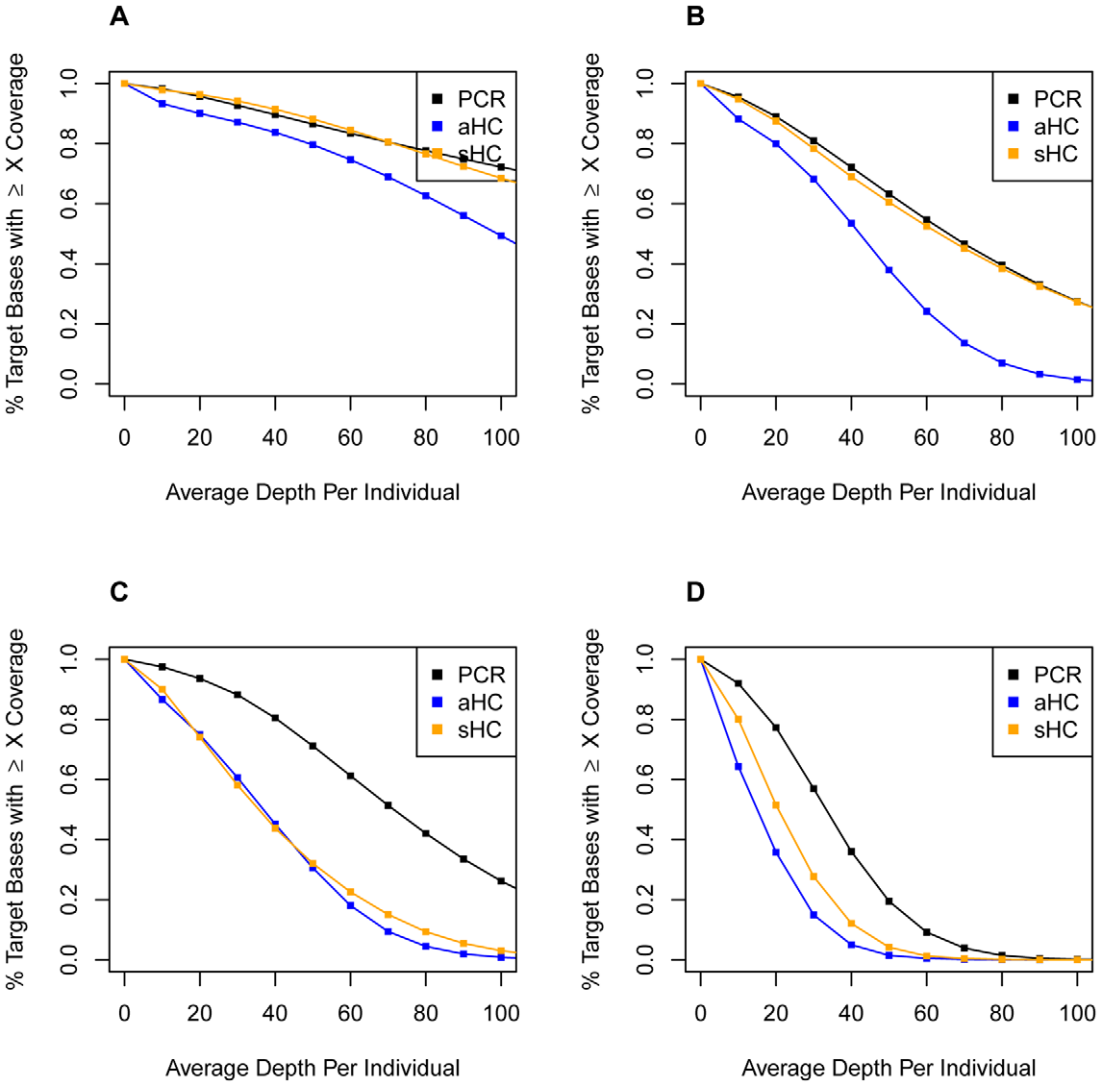
Target coverage per individual in pool before duplicate removal. This shows a cumulative relative frequency plot of the percentage of target bases with 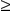
X coverage depth normalized by the number of individuals sequenced for: (A) Pool of 2, (B) Pool of 10, (C) Pool of 20 and (D) Pool of 50 individuals. The x-axis is in increments of 10× coverage. The black squares/lines illustrate the data for PCR enrichment, the blue squares/lines illustrate the data for aHC enrichment and the orange squares/lines illustrate the data for sHC enrichment. The first square represents the percentage of target bases with 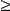
10× coverage per individual in the pool, and so on for each square in increments of 10×. This analysis assumes equal representation of each individual in the pool of DNA.

Target coverage depth directly affects the ability to detect variants, and depth is affected by the removal of potential duplicate reads. The higher enrichment specificity of PCR resulted in a higher overall mean read depth for target bases as compared to aHC and sHC, taking pool size and number of lanes sequenced into account regardless of duplicate read removal (Figure 1; Figures S1, S2, S3; Tables S7, S8). PCR yielded a higher percentage of target bases covered at 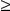
20× per individual across all pool sizes (Figure 1; Figure S1). However, target regions were not covered in a uniform way. For example, we found different coverage of protein coding versus non-coding target regions with duplicate read removal affecting the depth by approximately 100–200 reads but not the overall trend (Tables S9, S10; Figures S4, S5, S6, S7). Both aHC and sHC preferentially covered protein coding regions over non-coding regions across all pool sizes, whereas PCR demonstrated a bias in the opposite direction (Tables S9, S10; Figures S4, S5, S6, S7, S8, S9, S10, S11; t-test p-value
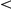
0.05 in all pools, for all methods). The same trends were observed in the technical replicates conducted (Figures S8, S9, S10, S11). An analysis of %GC, repeat and low complexity regions in the protein coding and non-coding target regions (Table S11) showed that non-coding DNA contains a higher proportion of repeat elements, thereby making it difficult to design highly specific oligonucleotide probes, affecting coverage for the hybrid capture methods. PCR experiments tended to favour the overall lower GC content of non-coding regions (Figures S12, S13, S14).

### Variant Detection Sensitivity and Specificity

Variant discovery is linked with coverage depth, but study design power importantly also depends on a balance between false positive and false negative variant discovery rates. A major reason for the removal of duplicate reads is to remove biases in variant detection and calling. To address issues related to removing duplicate reads in variant detection and frequency estimation in pooled targeted resequencing we analyzed all pools with the removal of duplicate reads before variant calling, and pools of 1, 10 and 50 individuals for the PCR and sHC enrichment without the removal of duplicate reads. We found the total number of called variants to increase with pool size, in keeping with the variants known to be present in each pool (Tables S12, S13). The removal of potential duplicate reads reduces the total number of variants called, with the effect being largest for PCR enrichment and for larger pools (Tables S12, S13). As the number of sequence-identified variants increased, the proportion present in dbSNP129 decreased regardless of duplicate read removal (Tables S14, S15). This trend could either be due to a higher false positive rate in larger pools, or to the fact that deep sequencing identified variants not present in dbSNP. We utilized HapMap, Illumina chip and 1KG data available for the pooled individuals to directly address questions of false positive and false negative rates (Table S1). sHC demonstrated the highest sensitivity to detect HapMap variants across all pool sizes and for both removing and not removing duplicate reads, except in the case of enriching a single individual after duplicate read removal (in which case aHC performed best; Table 2, Table S16). The removal of duplicate reads has a dramatic effect on the sensitivity in the pool of 1 enriched by PCR. Although the pre-duplicate read removal sensitivity is higher overall the difference in sensitivity is only approximately 1–3%. The same trend was observed when considering 1KG variants and the union of all known variants (Tables S19, S20, S21, S22).

**Table 2:** HapMap variation detection sensitivity after duplicate removal.

	Pool	Pool	Pool	Pool	Pool
	of 1	of 2	of 10	of 20	of 50
	(1089)^a^	(1459)^a^	(1999)^a^	(2067)^a^	(2145)^a^
PCR	26.26	87.46	92.35	96.27	95.80
aHC	97.15	85.33	96.60	97.82	94.41
sHC	94.12	95.07	98.30	98.16	96.88

This table contains the percentage of the known HapMap variants with at least one non-reference allele in the pool that each pool and enrichment method discovered (true positives). The false negative rate is 100 minus this value.

a : number of non-reference HapMap variants in pool.

We found that PCR had overall lower sensitivity to detect known singleton HapMap variants compared to HC methods (Table S23). Similarly, HC methods showed higher sensitivity to detect the variants identified in the single-individual pool particularly after duplicate read removal (Tables S24, S25), and sHC generally performed better than aHC. The ability to accurately call variants depends on sequence coverage, and the depth is affected by duplicate read removal. The read depth of false negative HapMap variants was significantly different to that of true positives, for both HC methods across pools of 2–50 individuals (Figures S15, S16, S17; data not shown pool of 2 and 50) (t-test p-value
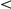
0.05 in all cases). A similar trend was observed for PCR (Figures S15, S16, S17). For both hybrid capture methods there was a trend towards a lower GC content in 200 base-pair regions around false negative HapMap variants compared to true positive variants, and the pattern was similar before and after duplicate read removal (Figures S18, S19, S20). This trend was not as prominent for the PCR experiments. The ability to call variants is also tied to the frequency of the variant in the pool. The false negative HapMap variants tended to have lower allele frequencies in the pools compared to true positives, and this trend was accentuated before duplicate read removal (Figures S21. S22, S23, S24, S25). This is in keeping with the fact that false negatives have lower depth coverage, making low frequency variant detection more difficult.

We found specificity (true negative rate), calculated on the basis of HapMap loci monomorphic in the pooled samples, to decrease as the complexity of the pool increased, and for a given pool the specificity was higher after duplicate read removal (Table 3, Table S17). False positives could be ascribed to genotype misclassification in HapMap or to sequencing error in our experiment. To resolve this, we examined data across 22 of the pooled samples present in both HapMap and 1KG. 1KG data corroborate the pooled sequencing findings across over 92% of overlapping loci for pools of more than one sample after duplicate reads are removed. For sHC, the concordance is 100% regardless of pool size when duplicate reads are removed, but is reduced to 95% when duplicates are included for the pool of 1 individual (Table 4, Table S18). The inclusion of duplicate reads uniformly increases the proportion of calls corroborated by 1KG for PCR. We examined the rate of genotype discordance between HapMap and 1KG at all sites in the regions examined for the 22 samples and found it to be 1.8%. Given the deep coverage of target bases in our experiment and concordance with 1KG we infer that the calculated false positive rates are likely to be overestimates.

**Table 3:** HapMap variation detection specificity after duplicate removal.

	Pool	Pool	Pool	Pool
	of 1	of 2	of 10	of 20
	(1722)^a^	(1353)^a^	(683)^a^	(590)^a^
PCR	99.88	98.97	97.66	96.95
aHC	98.84	98.67	97.22	96.61
sHC	99.07	98.74	97.22	96.95

This table contains the percentage of the known HapMap variants with no non-reference alleles and no missing genotypes in the pool that each pool and enrichment method correctly didn't call as a variant (true negatives). The false positive rate is 100 minus this value.

a : number of reference HapMap variants in pool.

**Table 4:** 1KG support for HapMap false positive loci after duplicate removal.

	Pool	Pool	Pool	Pool
	of 1	of 2	of 10	of 20
PCR	2(50%)	14(100%)	15(93.33%)	14(92.86%)
aHC	19(94.74%)	17(94.12%)	16(100%)	15(100%)
sHC	16(100%)	16(100%)	16(100%)	16(100%)

This table contains the number of loci considered false positives based on HapMap data that are present in 1KG and the percentage of these overlapping loci that the 1KG data supports the presence of non-reference alleles in the pool.

### Variant Frequency Estimation

**Figure 2:**
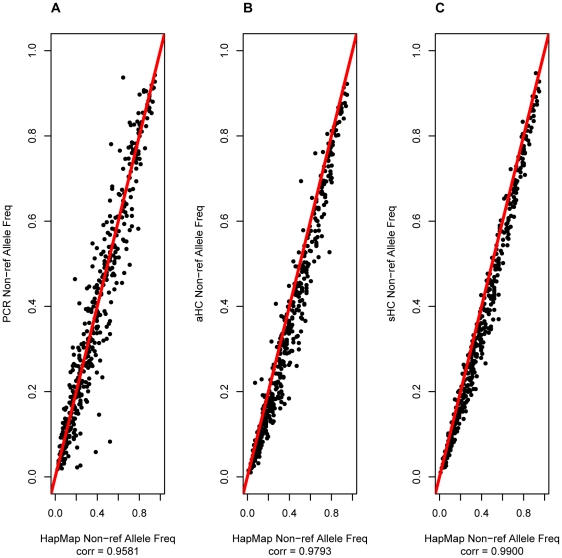
Accuracy of non-reference allele frequency estimation at HapMap/58C intersection variants for the Pool of 50 after duplicate removal. An analysis of the correlation between the non-reference allele frequency estimates from the sequencing based variant caller and the allele frequency calculated from the reference genotypes. The analysis includes the true positive variants called by the sequencing based variant caller for which there were 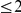 missing genotypes in the reference genotypes. The correlation coefficient is the Pearson's correlation coefficient. The figure shows the analysis for: (A) PCR enrichment, (B) aHC enrichment and (C) sHC enrichment.

**Figure 3:**
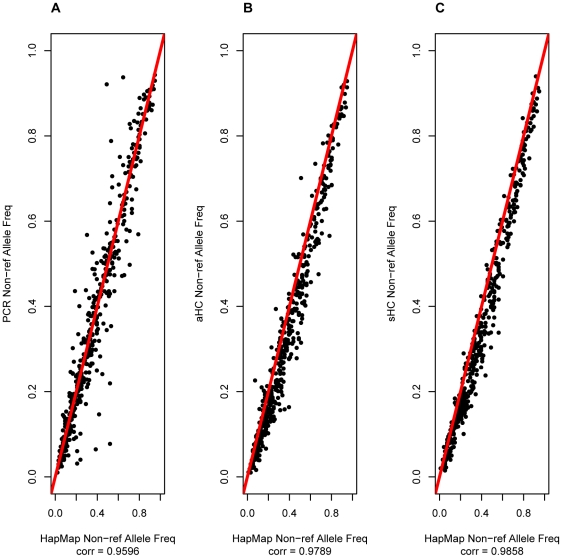
Accuracy of non-reference allele frequency estimation at HapMap/58C intersection variants for the Pool of 50 before duplicate removal. An analysis of the correlation between the non-reference allele frequency estimates from the sequencing based variant caller and the allele frequency calculated from the reference genotypes. The analysis includes the true positive variants called by the sequencing based variant caller for which there were 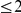 missing genotypes in the reference genotypes. The correlation coefficient is the Pearson's correlation coefficient. The figure shows the analysis for: (A) PCR, (B) aHC and (C) sHC enrichment.

The usefulness of pooled sequencing approaches in complex trait studies is primarily encapsulated by the ability to perform association tests through allele frequency estimate comparisons between pools of disease cases and controls. We compared estimated allele frequencies from the resequenced pools with those from HapMap and 58BC data and found that the sHC designs achieve the highest accuracy (Figures 2–3, Figures S26, S27, S28). The accuracy of frequency estimates improved with increasing pool size and was higher after duplicate read removal. The correlation between estimated allele frequency from sequencing the pool of 50 and from known genotypes was 95.8%, 97.9%, and 99.0% for PCR, aHC, and sHC respectively when duplicate reads were removed (Figure 2). However, when duplicate reads were included in the analysis the correlation in the same pool increased slightly for the PCR enrichment and dropped slightly for the HC methods (Figure 3). The decrease in correlation between true and estimated allele frequency pre-duplicate read removal was also seen for the pool of 10 individuals (Figures S26, S27). The allele frequency estimates appear to be stable and robust. For example, frequency estimates from the technical replicates of the Pool of 20 have a correlation of 98.59% for PCR and 99.31% for aHC (Figures S29, S30). Overall, pooled sequencing resulted in under-estimates of the true allele frequency regardless of duplicate read removal (Tables S26, S27).

We found the per-individual read depth at called variants to be weakly correlated with frequency estimate accuracy, and to vary across enrichment methods (Figures S31, S32, S33, S34). The inclusion of potential duplicate reads before the analysis increased this correlation (Figures S32, S35). There was a stronger correlation between the number of variant alleles in the pool and the accuracy of the allele frequency estimates (Figures S36, S37, S38, S39, S40). This correlation was also higher when potential duplicate reads were included in the analysis (Figures S37, S40). Interestingly, the higher the number of variant alleles in the pool, the worse the allele frequency estimates, a trend consistently observed across all enrichment methods and pool sizes. Specifically, we observed that low frequency variants tended to be more accurately estimated (Figures 2–3; Figures S26, S27, S28, S36, S37, S38, S39, S40).

### Reproducibility of Results

Reproducibility was assessed by performing technical replicates for PCR and aHC for the Pool of 20 individuals as a representative example. The HC replicates yielded more consistent results in terms of the number of reads produced and median coverage of target bases (Tables S5, S6). The sensitivity of HapMap variant detection varied by 4% between PCR replicates, and 2% between aHC replicates (Table S28). We next considered the number of variants that overlap between replicates as a function of the total number of unique variants called across replicates. The overlap rates of called variants across pairs of replicates were low (59%) for both PCR and aHC (Table S29). For variants called in both technical replicates the correlation between estimated allele frequencies was found to be high (98.6% and 99.3% for PCR and aHC respectively) (Figures S29, S30). When comparing allele frequencies for these overlapping variants (i.e. expecting identical estimates under an ideal experimental scenario), we found an average absolute allele frequency difference of 2.7% for PCR (across 7,233 overlapping variants) and 2.1% for aHC (6,713 variants) (Table S29).

### Cost

We compared the relative cost implications of the different study designs considered here. Considering the results after duplicate reads were removed, the Pool of 10 individuals had the highest sensitivity and specificity for pools greater than 1 individual but they were only 2% higher than the Pool of 50 which provided better allele frequency estimates and was more cost-effective. For example, for a pooling experiment involving 1000 cases and 1000 controls the Pool of 50 would be associated with 30% lower costs based on the number of sequencing lanes required as compared to the Pool of 10 and 86% lower costs than sequencing each individual on a single lane. Within each pool size, the cost of PCR was 3-fold more expensive than either of the hybrid-capture enrichment methods.

## Discussion

The field of human genetics is entering a new era of next-generation association studies. However, the cost of large-scale sequencing experiments of individual samples or indexed pools can be prohibitive, whilst the ability to accurately and inexpensively enrich and sequence targeted regions remains important to the research community. We have evaluated three enrichment methods in four non-indexed pool sizes to determine the best performing and most cost-effective strategy in the context of disease association studies.

The proportion of reads mapping to the target region, the uniformity of coverage of the target, and the read depth at targeted bases represent important measures of enrichment success. PCR yields 20–30% more on-target sequence reads than either aHC or sHC, resulting in a higher mean read depth for targeted bases. The hybrid capture methods show a bias for enrichment of protein coding versus non-coding target regions, and this difference can be explained by the high repeat content of non-coding regions. PCR shows the opposite bias, with non-coding regions covered at higher depth than coding regions, potentially ascribed to the lower GC content of non-coding regions.

The most relevant factors for disease association studies are variant detection sensitivity and specificity, and accuracy of allele frequency estimates. sHC shows the highest dbSNP129 overlap, and demonstrates the highest sensitivity and specificity for discovering HapMap and 1KG variants across all pool sizes. Similarly, sHC produces the best estimation of allele frequencies across the board. Allele frequency estimation appears to improve with increasing pool size, therefore arguing for pooling larger numbers of samples. Interestingly, low frequency variants appear to be better-estimated, potentially because of higher resolution to correctly call a smaller number of alternate alleles. The 2% average difference between allele frequencies across technical replicates indicates that estimates are not robust. A MAF difference at this scale could lead to false positive or false negative signals, particularly for variants at the lower end of the frequency spectrum, which are typically the focus of resequencing studies. Reviewed together, the results of our experiment indicate that in-solution hybrid capture in pools of 50 individuals has clear advantages over the alternative strategies considered here. Advances in sequencing and multiplexing protocols may have an effect on pool efficiency. We also conclude that non-indexed pooled resequencing studies are well-powered for variant discovery, but produce unreliable allele frequency estimates, particularly within the context of complex disease association studies.

## Materials and Methods

### Ethics Statement

This study has been approved by the ethics committee of the Wellcome Trust Sanger Institute (WTSI). This study only used extracted DNA from cell-lines, which falls outside of the UK Human Tissue Act. The use of the 1958BC samples is covered by a material transfer agreement (MTA) with the ALSPAC Laboratory, University of Bristol (the 1958BC sample custodian), which stated that the 1958BC had been collected under UK NHS Research Ethics Committee approval from SouthEast MREC, in Aug. 2002. REC Ref. MREC 01/1/44. The HapMap Populations/ELSI Group made recommendations for the HapMap project during the initial planning phase, and developed an informed consent form template (http://hapmap.ncbi.nlm.nih.gov/consent.html). The use of the HapMap CEU DNA is governed by these individually signed informed consent forms that grant permission for the use of the DNA in future studies approved by relevant ethics committees. The use of the HapMap DNAs were approved by the HapMap Repository (Coriell).

### DNA Samples

The samples sequenced consisted of 31 HapMap CEU individuals and 19 individuals from the 1958 British Birth Cohort (58BC). The HapMap DNA samples were obtained from Coriell Repositories and the sample IDs are: NA12249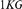, NA12156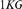, NA12004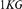, NA11831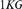, NA12716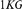, NA11832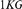, NA11993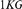, NA12057, NA11995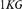, NA12006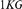, NA12144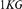, NA12802, NA12146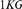, NA12005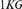, NA12003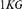, NA07000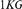, NA12043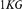, NA12044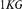, NA11992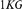, NA11881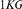, NA11994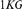, NA07345, NA12154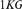, NA06994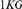, NA06985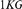, NA12239, NA07022, NA07034, NA12155, NA07056, NA06993. Individuals with a 1KG superscript were sequenced as part of pilot 1 of the 1,000 Genomes Project [4].

### Region Selection

The genomic regions selected for sequencing (Table S3) had shown suggestive evidence for association with type 2 diabetes following cumulative analysis of low frequency/rare variants directly typed on GWAS chips using a collapsing method [19]. Association in these regions did not replicate when further sample sets were tested. The targets for enrichment span 1.6 Mb in total and include entire genic regions that encompass 3′ and 5′ UTRs, introns, and exons, and have been defined as 50 Kb either side of the transcriptional start and stop sites.

### Array and Solution Oligonucleotide pool design

Genomic coordinates for the regions of interest were submitted to Nimblegen for the design of custom 385K arrays covering the target regions. Oligonucleotide pools for hybridization in solution phase were generated by Nimblegen to cover the same target regions. To cover real-estate on the array, three further regions were added on the hybrid-capture arm of the experiment (for a total of 1.96 Mb). These additional regions were excluded from the analysis presented here. This exclusion results in an under-estimation of the percentage of reads mapping back to target for the aHC and sHC experiments in Table 1.

### Preparation of the pools

Each DNA sample was quantified using standard picogreen protocols and normalized to 50 ng/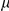
l. The pools were generated by mixing the required volumes of the appropriate number of samples to give a final concentration of each pool of 50 ng/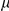
l. The concentration of the resulting pool was checked using picogreen. Aliquots of the same pool were used for both PCR and hybrid-capture.

### PCR

Primers were designed automatically using Primer 3 to achieve a 5-fold depth of 5- and 10 kb amplicons across the target regions. Where necessary, manual primer design of 5 kb amplicons using Primer 3 was used to fill any gaps in the coverage following the automatic design. In total 462×10 kb STSs and 737×5 kb STSs were designed automatically and 88×5 kb STSs manually. All primers were pre-screened on a set of four genomic DNAs. Products were separated on an 0.8% agarose gel, visualised with ethidium bromide staining and scored as pass/weak/fail. Based on the prescreening results a final set of STSs were chosen to give 3-fold coverage over the target regions which consisted of 256×10 kb STSs and 256×5 kb STSs. Aliquots of the same DNA pools used for hybrid capture were used as template for PCR amplification with each STS. 5 kb amplicons were amplified as follows: Primers were pre-aliquoted at a concentration of 10 ng/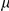
l, 4 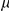
l per well into 384-well PCR plates. A premix was made consisting of 2 µl of 10× Buffer (as supplied with the enzyme), 0.4 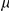
l 10 mM dNTPs, 0.8 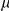
l 50 mM MgSO4 (as supplied with the enzyme), 0.12 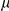
l Platinum Hi-Fi Taq, 11.8 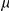
l DDW and 30 ng of pooled DNA per reaction and added to the pre-aliquoted primers. PCR cycling conditions were as follows: 98°C for 3 minutes, followed by 15 cycles of 94°C for 30 seconds, 68°C for 30 seconds, with the annealing temperature decreasing by 1oC per cycle, 68°C for 5 minutes followed by 19 cycles of 94°C for 30 seconds, 58°C for 30 seconds, 68°C for 5 minutes followed by 68°C for 10 minutes. 10 kb amplicons were amplified as follows: Primers were pre-aliquoted at a concentration of 10 ng/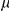
l, 4 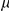
l per well into 384-well PCR plates. A premix was made consisting of 2 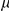
l of 10× Buffer (as supplied with the enzyme), 0.4 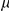
l 10 mM dNTPs, 0.8 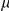
l 50 mM MgSO4 (as supplied with the enzyme), 0.16 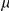
l Platinum Hi-Fi Taq, 11.14 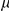
l DDW and 90 ng of pooled DNA per reaction and added to the pre-aliquoted primers. PCR cycling conditions for were as follows: 98°C for 3 minutes, followed by 15 cycles of 94°C for 30 seconds, 68°C for 30 seconds, with the annealing temperature decreasing by 1oC per cycle, 68°C for 10 minutes followed by 19 cycles of 94°C for 30 seconds, 58°C for 30 seconds, 68°C for 10 minutes followed by 68°C for 10 minutes. Products were visualised using ethidium bromide staining. PCR products from each DNA pool for all STSs were pooled together in equimolar amounts and used to construct an Illumina library prior to sequencing as described below.

### Illumina Library Construction

20 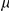
g of DNA were sheared to 100–400 bp using a Covaris S2 following manufacturer's protocols and the settings Duty Cycle, 20%; Intensity, 5.0; Cycles/burst, 200; Duration, 90; Mode, Freq Sweeping. Sheared samples were quantitated on a Bioanalyzer (Agilent, Santa Clara, USA). 10–15 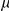
g of sheared DNA were end-repaired, A-tailed and Illumina sequencing adapters ligated to the resulting fragments using the Illumina Paired-End DNA Sample Prep protocol with the slight modification that the gel size selection step was replaced with a SPRI bead purification (following manufacturer's protocol).

### Array Hybridization

5 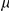
g of each library were hybridized to a custom Nimblegen 385-K array following manufacturer's protocols (Roche/Nimblegen) with the modification that no pre-hybridization PCR was performed. Captured samples were washed and eluted in 50 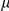
l of PCR-Grade water following manufacturer's protocols. Eluted samples were amplified using a master-mix containing 2 mM MgCl2, 0.2 mM dNTPs, 0.5 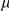
M PE.1. 0.5 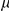
M PE.2 and 3 units of Platinum® Pfx DNA Polymerase per sample. Samples were aliquoted into 3 individual wells of a plate and amplified using the following conditions: 94°C for 5 minutes followed by 20 cycles of 94°C for 15 seconds, 58°C for 30 seconds, 72°C for 30 seconds and a final extension of 72°C for 5 minutes. PCR products were purified using SPRI beads prior to sequencing.

### Solution Hybridization

1 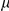
g of each library was hybridized to an oligo pool following manufacturer's protocols with the modifications that 14 cycles of pre-hybridization PCR were performed and 50× COT1DNA was used in the hybridization. Following hybridization the captured samples bound to the Streptavidin beads were washed following manufacturer's protocols. Post-capture PCR was performed on the captured samples bound to the beads as described above.

### Sequencing

Captured libraries were sequenced on the Illumina Genome Analyzer II (GAII) platform as paired-end 37-bp or 54-bp reads, following manufacturer's protocols. The raw sequencing reads are available through the European Genome-Phenome Archive (http://www.ebi.ac.uk/ega, accession EGAS00001000134) and the European Nucleotide Archive (http://www.ebi.ac.uk/ena, accession ERP000770).

### Read Mapping and Sequence Analysis

The reference human genome used in these analyses was UCSC assembly hg18 (NCBI Build 36), including unordered sequence. Each lane of sequencing was mapped to the reference genome using Maq (v0.7.1) with default parameters [20]. For pools with multiple lanes of sequencing, the individual lane mappings were merged with the Maq utility mapmerge. The phred-scaled base quality scores from the GAII were recalibrated using the Quality Score Recalibration tool in the Genome Analysis Toolkit (v1.0 build January 21, 2010) [21]. Duplicate reads were identified and marked using Picard (v1.17; http://picard.sourceforge.net/), and for a subset of the analyses duplicates were removed with SamTools (v0.1.7) [22]. The number of reads mapped and mapped to target regions was calculated using the view utility in SamTools. The %GC versus coverage analysis was performed using the CollectGcBiasMetrics utility in Picard. The analysis of the repeat and low-complexity content of the coding and non-coding target regions were performed with the RepeatMasker software (v. open-3.2.9) [23].

### Variant Calling and Frequency Estimation

Variants were called on the merged BAM file from all lanes for a pool. The BAM file used to call variants had recalibrated base quality scores, reads mapping off the end of the reference soft-clipped, and either duplicate reads marked or removed. The variant calling and frequency estimation was performed by Syzygy (v0.9.5.39) using the default parameters. Syzygy calls single nucleotide variants and single base insertion/deletions [7] (http://www.broadinstitute.org/software/syzygy/). This analysis only considered Syzygy single nucleotide variant calls. Variants are defined as a locus having 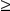
1 non-reference allele, an allele different than the reference genome used for mapping, present in the pool. Syzygy assigns a confidence score to all variant calls (high, medium and low). We analyzed all the called variants regardless of confidence.

### Comparison Genotypes

The sensitivity, specificity and frequency estimation analyses were conducted by comparing the variants and frequency estimates from the Syzygy calls to the known variant content in the pool using existing genotype data for each pooled individual. We used the non-redundant release 27 HapMap genotypes for the 31 HapMap individuals used in the pooling experiments. The genotypes were mapped to the forward strand of Build 36 of the reference genome and sensitivity analysis included all loci where the HapMap genotypes indicated that there was at least one non-reference base in the pool, whereas the specificity and allele frequency estimation analysis only included loci where all individuals in the pool had non-missing genotype data. Twenty-two of the HapMap individuals used in our pooling experiments were sequenced in Pilot 1 of the 1,000 Genomes Project. We used 1KG genotypes4 for these individuals from the final pilot 1 call set released March 28, 2010. Due to the fact that no pool consisted solely of individuals sequenced in 1KG, we are unable to perform specificity analysis for the 1KG loci. The 1958 Birth Cohort (58BC) genotypes came from 2 sources. Sixteen of the pooled individuals were genotyped as part of the Wellcome Trust Case Control Consortium 2 (WTCCC2; Illumina 1.2 M Duo platform) [24] and 6 individuals were genotyped as part of this project at the Wellcome Trust Sanger Institute (Illumina 670K platform). The WTCCC2 genotypes were downloaded from the European Genotype Archive (http://www.ebi.ac.uk/ega/). The frequency estimation and variant discovery specificity analysis for the pool of 50 was based on the intersection of variants that occurred in both the HapMap and 58BC genotype sets. The variant discovery sensitivity analysis for the pool of 50 was carried out by taking the union of variants in 1KG, HapMap and 58BC genotype sets. The dbSNP variants used were dbSNP129 variants downloaded from the UCSC genome browser, with all rsIDs that mapped 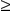
2 locations in the genome removed (referred to as the non-redundant dbSNP129). The coding/non-coding analysis was performed by defining coding intervals for each gene as per the March 27, 2009 release of the consensus coding sequence (CCDS) project [25].

### Statistical Sequence and Variant Analysis

All statistical analyses were performed with the R statistical software package [26]. The target regions and called variants were separated into different subsets and two-sided, two-sample t-tests with unequal variances were performed to assess differences in the means of the distributions. An obtained t-test p-value of 0 indicates that the p-value of the test was more significant than the statistical software R would calculate (the highest exponent on the machine used for calculation is 1024). The correlation coefficients reported in Figure 2 and Figures S15, S16, S17, S18, S19, S20, S21, S22, S23, S24 are Pearson's correlation coefficients. Figures S19, S20, S21 further investigate the relationship between individual read depth and allele frequency accuracy, defined as the HapMap frequency minus the Syzygy estimated frequency, by a least squares fitting of the model, 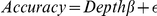, and the red lines in these figures shows the resulting estimate of the intercept and 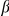. Figures S22, S23, S24 further investigate the relationship between allele count and allele frequency accuracy, as defined above, by a least squares fitting of the model, 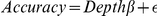, and the red lines in these figures shows the resulting estimate of the intercept and 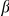.

## Supporting Information

Figure S1
**Target coverage per individual in pool after duplicate removal.** This shows a cumulative relative frequency plot of the percentage of target bases with 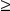
X coverage depth normalized by the number of individuals sequenced for: (A) Pool of 2, (B) Pool of 10, (C) Pool of 20 and (D) Pool of 50 individuals. The x-axis is in increments of 10× coverage. The black squares/lines illustrate the data for PCR enrichment, the blue squares/lines illustrate the data for aHC enrichment and the orange squares/lines illustrate the data for sHC enrichment. The first square represents the percentage of target bases with 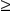
10× coverage per individual in the pool, and so on for each square in increments of 10×. This analysis assumes equal representation of each individual in the pool of DNA.(TIF)

Figure S2
**Target coverage per lane of sequencing before duplicate removal.** This shows a cumulative relative frequency plot of the percentage of target bases with 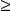
X coverage depth normalized by the number of lanes sequenced for: (A) Pool of 2, (B) Pool of 10, (C) Pool of 20 and (D) Pool of 50 individuals. The x-axis is in increments of 10× coverage. The black squares/lines illustrate the data for PCR enrichment, the blue squares/lines illustrate the data for aHC enrichment and the orange squares/lines illustrate the data for sHC enrichment. The first square represents the percentage of target bases with 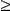
10× coverage per lane sequenced, and so on for each square in increments of 10×.(TIF)

Figure S3
**Target coverage per lane of sequencing after duplicate removal.** This shows a cumulative relative frequency plot of the percentage of target bases with 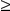
X coverage depth normalized by the number of lanes sequenced after duplicate removal for: (A) Pool of 2, (B) Pool of 10, (C) Pool of 20 and (D) Pool of 50 individuals. The x-axis is in increments of 10× coverage. The black squares/lines illustrate the data for PCR enrichment, the blue squares/lines illustrate the data for aHC enrichment and the orange squares/lines illustrate the data for sHC enrichment. The first square represents the percentage of target bases with 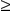
10× coverage per lane sequenced, and so on for each square in increments of 10×.(TIF)

Figure S4
**Pool of 20 coding vs. non-coding target coverage per lane after duplicate removal.** This figure shows a cumulative relative frequency plot of the percentage of target bases with 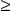
X coverage depth normalized by the number of lanes sequenced after duplicate removal for the Pool of 20 individuals for: (A) PCR, (B) aHC and (C) sHC enrichment. The orange squares/lines illustrate the data for protein coding target bases and the black squares/lines illustrate the data for the non-coding target bases. The first square represents the percentage of target bases with 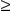
10× coverage per lane in the pool, and so on for each square in increments of 10×.(TIF)

Figure S5
**Pool of 20 coding vs. non-coding target coverage per lane before duplicate removal.** This figure shows a cumulative relative frequency plot of the percentage of target bases with 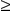
X coverage depth normalized by the number of lanes sequenced for the Pool of 20 individuals for: (A) PCR, (B) aHC and (C) sHC enrichment. The orange squares/lines illustrate the data for protein coding target bases and the black squares/lines illustrate the data for the non-coding target bases. The first square represents the percentage of target bases with 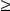
10× coverage per lane in the pool, and so on for each square in increments of 10×.(TIF)

Figure S6
**Pool of 50 coding vs. non-coding target coverage per lane after duplicate removal.** This figure shows a cumulative relative frequency plot of the percentage of target bases with 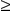
X coverage depth normalized by the number of lanes sequenced after duplicate removal for the Pool of 50 individuals for: (A) PCR, (B) aHC and (C) sHC enrichment. The orange squares/lines illustrate the data for protein coding target bases and the black squares/lines illustrate the data for the non-coding target bases. The first square represents the percentage of target bases with 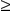
10× coverage per lane in the pool, and so on for each square in increments of 10×.(TIF)

Figure S7
**Pool of 50 coding vs. non-coding target coverage per lane before duplicate removal.** This figure shows a cumulative relative frequency plot of the percentage of target bases with 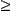
X coverage depth normalized by the number of lanes sequenced for the Pool of 50 individuals for: (A) PCR, (B) aHC and (C) sHC enrichment. The orange squares/lines illustrate the data for protein coding target bases and the black squares/lines illustrate the data for the non-coding target bases. The first square represents the percentage of target bases with 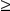
10× coverage per lane in the pool, and so on for each square in increments of 10×.(TIF)

Figure S8
**Pool of 20 PCR replicates coding vs. non-coding target coverage per lane after duplicate removal.** This figure shows a cumulative relative frequency plot of the percentage of target bases with 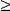
X coverage depth normalized by the number of lanes sequenced for the Pool of 20 individuals PCR replicates for: (A) Replicate 1, (B) Replicate 2. Replicate 1 is the replicate used in all the main analyses. The orange squares/lines illustrate the data for protein coding target bases and the black squares/lines illustrate the data for the non-coding target bases. The first square represents the percentage of target bases with 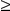
10× coverage per lane in the pool, and so on for each square in increments of 10×.(TIF)

Figure S9
**Pool of 20 PCR replicates coding vs. non-coding target coverage per lane before duplicate removal.** This figure shows a cumulative relative frequency plot of the percentage of target bases with 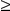
X coverage depth normalized by the number of lanes sequenced for the Pool of 20 individuals PCR replicates for: (A) Replicate 1, (B) Replicate 2. Replicate 1 is the replicate used in all the main analyses. The orange squares/lines illustrate the data for protein coding target bases and the black squares/lines illustrate the data for the non-coding target bases. The first square represents the percentage of target bases with 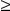
10× coverage per lane in the pool, and so on for each square in increments of 10×.(TIF)

Figure S10
**Pool of 20 aHC replicates coding vs. non-coding target coverage per lane after duplicate removal.** This figure shows a cumulative relative frequency plot of the percentage of target bases with 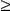
X coverage depth normalized by the number of lanes sequenced for the Pool of 20 individuals aHC replicates for: (A) Replicate 1, (B) Replicate 2. Replicate 1 is the replicate used in all the main analyses. The orange squares/lines illustrate the data for protein coding target bases and the black squares/lines illustrate the data for the non-coding target bases. The first square represents the percentage of target bases with 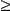
10× coverage per lane in the pool, and so on for each square in increments of 10×.(TIF)

Figure S11
**Pool of 20 aHC replicates coding vs. non-coding target coverage per lane before duplicate removal.** This figure shows a cumulative relative frequency plot of the percentage of target bases with 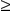
X coverage depth normalized by the number of lanes sequenced for the Pool of 20 individuals aHC replicates for: (A) Replicate 1, (B) Replicate 2. Replicate 1 is the replicate used in all the main analyses. The orange squares/lines illustrate the data for protein coding target bases and the black squares/lines illustrate the data for the non-coding target bases. The first square represents the percentage of target bases with 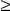
10× coverage per lane in the pool, and so on for each square in increments of 10×.(TIF)

Figure S12 **Pool of 20 genomic coverage as function of %GC of reference after duplicate removal.** This figure analyzes the normalized coverage and mean base quality of mapped bases compared to the percentage of GC bases for the reference genome divided into 500 base-pair windows in the Pool of 20 individuals for: (A) PCR, (B) aHC and (C) sHC enrichment. Normalized coverage for a %GC bin is the proportion of coverage this window accounts for relative to the mean coverage across all %GC bins.
(TIF)

Figure S13 **Pool of 50 genomic coverage as function of %GC of reference after duplicate removal.** This figure analyzes the normalized coverage and mean base quality of mapped bases compared to the percentage of GC bases for the reference genome divided into 500 base-pair windows in the Pool of 50 individuals for: (A) PCR, (B) aHC and (C) sHC enrichment. Normalized coverage for a %GC bin is the proportion of coverage this window accounts for relative to the mean coverage across all %GC bins.
(TIF)

Figure S14 **Pool of 50 genomic coverage as function of %GC of reference before duplicate removal.** This figure analyzes the normalized coverage and mean base quality of mapped bases compared to the percentage of GC bases for the reference genome divided into 500 base-pair windows in the Pool of 50 individuals for: (A) PCR, (B) aHC and (C) sHC enrichment. Normalized coverage for a %GC bin is the proportion of coverage this window accounts for relative to the mean coverage across all %GC bins.
(TIF)

Figure S15
**Pool of 10 per individual coverage at HapMap true positive, false positive and false negative variants after duplicate removal.** This figure shows a cumulative relative frequency plot of the percentage of variants with 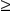
X coverage per individual in the pool at HapMap true positive, false positive and false negative variants for: (A) PCR, (B) aHC and (C) sHC enrichment. The black squares/lines illustrate the data for false negative variants, the blue squares/lines illustrate the data for false positive variants and the orange squares/lines illustrate the data for true positive variants. The first square represents the percentage of variants in a class with 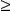
10× coverage per individual in the pool, and so on for each square in increments of 10×.(TIF)

Figure S16
**Pool of 10 per individual coverage at HapMap true positive, false positive and false negative variants before duplicate removal.** This figure shows a cumulative relative frequency plot of the percentage of variants with 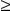
X coverage per individual in the pool at HapMap true positive, false positive and false negative variants for: (A) PCR and (B) sHC enrichment. The black squares/lines illustrate the data for false negative variants, the blue squares/lines illustrate the data for false positive variants and the orange squares/lines illustrate the data for true positive variants. The first square represents the percentage of variants in a class with 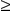 10× coverage per individual in the pool, and so on for each square in increments of 10×.(TIF)

Figure S17
**Pool of 20 per individual coverage at HapMap true positive, false positive and false negative variants after duplicate removal.** This figure shows a cumulative relative frequency plot of the percentage of variants with 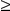
X coverage per individual in the pool at HapMap true positive, false positive and false negative variants for: (A) PCR, (B) aHC and (C) sHC enrichment. The black squares/lines illustrate the data for false negative variants, the blue squares/lines illustrate the data for false positive variants and the orange squares/lines illustrate the data for true positive variants. The first square represents the percentage of variants in a class with 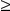
10× coverage per individual in the pool, and so on for each square in increments of 10×.(TIF)

Figure S18
**Pool of 10 %GC context at HapMap true positive, false positive and false negative variants after duplicate removal.** This figure shows a cumulative relative frequency plot of the percentage of variants with a genomic context %GC of 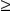
X% in a window of 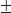
100 base-pairs around each HapMap true positive, false positive and false negative variants for: (A) PCR, (B) aHC and (C) sHC enrichment. The black squares/lines illustrate the data for false negative variants, the blue squares/lines illustrate the data for false positive variants and the orange squares/lines illustrate the data for true positive variants. The first square represents the percentage of variants in a class with 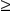
10% GC in a 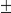
100 base-pair window around a variant coverage, and so on for each square in increments of 10% GC content.(TIF)

Figure S19
**Pool of 10 %GC context at HapMap true positive, false positive and false negative variants before duplicate removal.** This figure shows a cumulative relative frequency plot of the percentage of variants with a genomic context %GC of 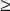
X% in a window of 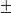
100 base-pairs around each HapMap true positive, false positive and false negative variants for: (A) PCR and (B) sHC enrichment. The black squares/lines illustrate the data for false negative variants, the blue squares/lines illustrate the data for false positive variants and the orange squares/lines illustrate the data for true positive variants. The first square represents the percentage of variants in a class with 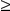
10% GC in a 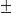
100 base-pair window around a variant coverage, and so on for each square in increments of 10% GC content.(TIF)

Figure S20
**Pool of 20 %GC context at HapMap true positive, false positive and false negative variants after duplicate removal.** This figure shows a cumulative relative frequency plot of the percentage of variants with a genomic context %GC of 
X% in a window of 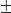
100 base-pairs around each HapMap true positive, false positive and false negative variants for: (A) PCR, (B) aHC and (C) sHC enrichment. The black squares/lines illustrate the data for false negative variants, the blue squares/lines illustrate the data for false positive variants and the orange squares/lines illustrate the data for true positive variants. The first square represents the percentage of variants in a class with 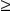
10% GC in a 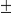
100 base-pair window around a variant coverage, and so on for each square in increments of 10% GC content.(TIF)

Figure S21
**HapMap frequency distribution of true positive and false negative variants in the Pool of 10 after duplicate removal.** This figure shows a cumulative relative frequency plot of the percentage of variants with true allele frequency 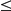
X in the pool at HapMap true positive and false negative variants for: (A) PCR, (B) aHC and (C) sHC enrichment. The black squares/lines illustrate the data for false negative variants and the orange squares/lines illustrate the data for true positive variants. The first square represents the percentage of variants in a class with allele frequency 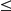
0.01, and so on for each square in 0.01 frequency increments.(TIF)

Figure S22
**HapMap frequency distribution of true positive and false negative variants in the Pool of 10 before duplicate removal.** This figure shows a cumulative relative frequency plot of the percentage of variants with true allele frequency 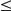
X in the pool at HapMap true positive and false negative variants for: (A) PCR and (B) sHC enrichment. The black squares/lines illustrate the data for false negative variants and the orange squares/lines illustrate the data for true positive variants. The first square represents the percentage of variants in a class with allele frequency 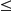
0.01, and so on for each square in 0.01 frequency increments.(TIF)

Figure S23
**HapMap frequency distribution of true positive and false negative variants in the Pool of 20 after duplicate removal.** This figure shows a cumulative relative frequency plot of the percentage of variants with true allele frequency 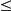
X in the pool at HapMap true positive and false negative variants for: (A) PCR, (B) aHC and (C) sHC enrichment. The black squares/lines illustrate the data for false negative variants and the orange squares/lines illustrate the data for true positive variants. The first square represents the percentage of variants in a class with allele frequency 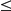
0.01, and so on for each square in 0.01 frequency increments.(TIF)

Figure S24
**HapMap/58C intersection frequency distribution of true positive and false negative variants in the Pool of 50 after duplicate removal.** This figure shows a cumulative relative frequency plot of the percentage of variants with true allele frequency 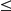
X in the pool at HapMap/58C intersection true positive and false negative variants for: (A) PCR, (B) aHC and (C) sHC enrichment. The black squares/lines illustrate the data for false negative variants and the orange squares/lines illustrate the data for true positive variants. The first square represents the percentage of variants in a class with allele frequency 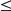
0.01, and so on for each square in 0.01 frequency increments. This analysis is for the 507 sites where all 50 individuals had genotype data for, which lead to only 14 false negatives for PCR, 8 false negatives for aHC and 1 false negative for sHC.(TIF)

Figure S25
**HapMap/58C intersection frequency distribution of true positive and false negative variants in the Pool of 50 before duplicate removal.** This figure shows a cumulative relative frequency plot of the percentage of variants with true allele frequency 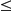
X in the pool at HapMap/58C intersection true positive and false negative variants for: (A) PCR, (B) aHC and (C) sHC enrichment. The black squares/lines illustrate the data for false negative variants and the orange squares/lines illustrate the data for true positive variants. The first square represents the percentage of variants in a class with allele frequency 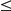
0.01, and so on for each square in 0.01 frequency increments. This analysis is for the 507 sites where all 50 individuals had genotype data for, which lead to only 11 false negatives for PCR, 1 false negatives for aHC and 1 false negative for sHC.(TIF)

Figure S26 **Accuracy of non-reference allele frequency estimation at HapMap variants for the Pool of 10 after duplicate removal.** An analysis of the correlation between the non-reference allele frequency estimate from the sequencing based variant caller and the allele frequency from the reference genotypes. The analysis includes the true positive variants called by the sequencing based variant caller for which there were no missing genotypes in the reference genotypes. The correlation coefficient is the Pearson's correlation coefficient. The figure shows the analysis for: (A) PCR, (B) aHC and (C) sHC enrichment.
(TIF)

Figure S27 **Accuracy of non-reference allele frequency estimation at HapMap variants for the Pool of 10 before duplicate removal.** An analysis of the correlation between the non-reference allele frequency estimate from the sequencing based variant caller and the allele frequency from the reference genotypes. The analysis includes the true positive variants called by the sequencing based variant caller for which there were no missing genotypes in the reference genotypes. The correlation coefficient is the Pearson's correlation coefficient. The figure shows the analysis for: (A) PCR and (B) sHC enrichment.
(TIF)

Figure S28 **Accuracy of non-reference allele frequency estimation at HapMap variants for the Pool of 20 after duplicate removal.** An analysis of the correlation between the non-reference allele frequency estimate from the sequencing based variant caller and the allele frequency from the reference genotypes. The analysis includes the true positive variants called by the sequencing based variant caller for which there were no missing genotypes in the reference genotypes. The correlation coefficient is the Pearson's correlation coefficient. The figure shows the analysis for: (A) PCR, (B) aHC and (C) sHC enrichment.
(TIF)

Figure S29 **Comparison of non-reference allele frequency estimation for Pool of 20 PCR technical replicates after duplicate removal.** The correlation of non-reference allele frequency estimates for overlapping variants between the PCR technical replicates. The y-axis are the non-reference allele frequencies for replicate 2 and the x-axis are the non-reference allele frequencies for replicate 1. The correlation is the Pearson' correlation coefficient between allele frequencies.
(TIF)

Figure S30 **Comparison of non-reference allele frequency estimation for Pool of 20 aHC technical replicates after duplicate removal.** The correlation of non-reference allele frequency estimates for overlapping variants between the aHC technical replicates. The y-axis are the non-reference allele frequencies for replicate 2 and the x-axis are the non-reference allele frequencies for replicate 1. The correlation is the Pearson' correlation coefficient between allele frequencies.
(TIF)

Figure S31
**HapMap allele frequency estimation accuracy as a function of per individual depth in the Pool of 10 after duplicate removal.** This figure is a scatter plot of the accuracy of the allele frequency estimates from the sequencing compared to the per individual read depth at HapMap true positive variants in the Pool of 10 individuals for: (A) PCR, (B) aHC and (C) sHC enrichment. The accuracy of the estimates are calculated as the frequency calculated from the HapMap genotypes minus the frequency estimated from the sequencing data. The y-axis is the accuracy value and the x-axis is the per individual read depth in the pool. The red line is the least squares fit of the model 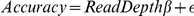, and the corr is the Pearson's correlation coefficient between the accuracy and read depth.(TIF)

Figure S32
**HapMap allele frequency estimation accuracy as a function of per individual depth in the Pool of 10 before duplicate removal.** This figure is a scatter plot of the accuracy of the allele frequency estimates from the sequencing compared to the per individual read depth at HapMap true positive variants in the Pool of 10 individuals for: (A) PCR and (B) sHC enrichment. The accuracy of the estimates are calculated as the frequency calculated from the HapMap genotypes minus the frequency estimated from the sequencing data. The y-axis is the accuracy value and the x-axis is the per individual read depth in the pool. The red line is the least squares fit of the model 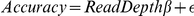, and the corr is the Pearson's correlation coefficient between the accuracy and read depth.(TIF)

Figure S33
**HapMap allele frequency estimation accuracy as a function of per individual depth in the Pool of 20 after duplicate removal.** This figure is a scatter plot of the accuracy of the allele frequency estimates from the sequencing compared to the per individual read depth at HapMap true positive variants in the Pool of 20 individuals for: (A) PCR, (B) aHC and (C) sHC enrichment. The accuracy of the estimates are calculated as the frequency calculated from the HapMap genotypes minus the frequency estimated from the sequencing data. The y-axis is the accuracy value and the x-axis is the per individual read depth in the pool. The red line is the least squares fit of the model 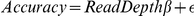, and the corr is the Pearson's correlation coefficient between the accuracy and read depth.(TIF)

Figure S34
**HapMap/58BC intersection allele frequency estimation accuracy as a function of per individual depth in the Pool of 50 after duplicate removal.** This figure is a scatter plot of the accuracy of the allele frequency estimates from the sequencing compared to the per individual read depth at HapMap/58BC intersection true positive variants in the Pool of 50 individuals for: (A) PCR, (B) aHC and (C) sHC enrichment. The accuracy of the estimates are calculated as the frequency calculated from the HapMap genotypes minus the frequency estimated from the sequencing data. The y-axis is the accuracy value and the x-axis is the per individual read depth in the pool. The red line is the least squares fit of the model 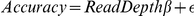, and the corr is the Pearson's correlation coefficient between the accuracy and read depth.(TIF)

Figure S35
**HapMap/58BC intersection allele frequency estimation accuracy as a function of per individual depth in the Pool of 50 before duplicate removal.** This figure is a scatter plot of the accuracy of the allele frequency estimates from the sequencing compared to the per individual read depth at HapMap/58BC intersection true positive variants in the Pool of 50 individuals for: (A) PCR, (B) aHC and (C) sHC enrichment. The accuracy of the estimates are calculated as the frequency calculated from the HapMap genotypes minus the frequency estimated from the sequencing data. The y-axis is the accuracy value and the x-axis is the per individual read depth in the pool. The red line is the least squares fit of the model 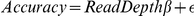, and the corr is the Pearson's correlation coefficient between the accuracy and read depth.(TIF)

Figure S36
**HapMap allele frequency estimation accuracy as a function of allele count in the Pool of 10 after duplicate removal.** This figure is a scatter plot of the accuracy of the allele frequency estimates from the sequencing compared to the number of variant alleles at HapMap true positive variants in the Pool of 10 individuals for: (A) PCR, (B) aHC and (C) sHC enrichment. The accuracy of the estimates are calculated as the frequency calculated from the HapMap genotypes minus the frequency estimated from the sequencing data. The y-axis is the accuracy value and the x-axis is the number of variant alleles in the pool. The red line is the least squares fit of the model 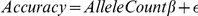, and the corr is the Pearson's correlation coefficient between the accuracy and read depth.(TIF)

Figure S37
**HapMap allele frequency estimation accuracy as a function of allele count in the Pool of 10 before duplicate removal.** This figure is a scatter plot of the accuracy of the allele frequency estimates from the sequencing compared to the number of variant alleles at HapMap true positive variants in the Pool of 10 individuals for: (A) PCR and (B) sHC enrichment. The accuracy of the estimates are calculated as the frequency calculated from the HapMap genotypes minus the frequency estimated from the sequencing data. The y-axis is the accuracy value and the x-axis is the number of variant alleles in the pool. The red line is the least squares fit of the model 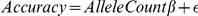, and the corr is the Pearson's correlation coefficient between the accuracy and read depth.(TIF)

Figure S38
**HapMap allele frequency estimation accuracy as a function of allele count in the Pool of 20 after duplicate removal.** This figure is a scatter plot of the accuracy of the allele frequency estimates from the sequencing compared to the number of variant alleles at HapMap true positive variants in the Pool of 20 individuals for: (A) PCR, (B) aHC and (C) sHC enrichment. The accuracy of the estimates are calculated as the frequency calculated from the HapMap genotypes minus the frequency estimated from the sequencing data. The y-axis is the accuracy value and the x-axis is the number of variant alleles in the pool. The red line is the least squares fit of the model 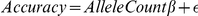, and the corr is the Pearson's correlation coefficient between the accuracy and read depth.(TIF)

Figure S39
**HapMap/58BC intersection allele frequency estimation accuracy as a function of allele count in the Pool of 50 after duplicate removal.** This figure is a scatter plot of the accuracy of the allele frequency estimates from the sequencing compared to the number of variant alleles at HapMap/58BC intersection true positive variants in the Pool of 50 individuals for: (A) PCR, (B) aHC and (C) sHC enrichment. The accuracy of the estimates are calculated as the frequency calculated from the HapMap genotypes minus the frequency estimated from the sequencing data. The y-axis is the accuracy value and the x-axis is the number of variant alleles in the pool. The red line is the least squares fit of the model 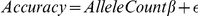, and the corr is the Pearson's correlation coefficient between the accuracy and read depth.(TIF)

Figure S40
**HapMap/58BC intersection allele frequency estimation accuracy as a function of allele count in the Pool of 50 before duplicate removal.** This figure is a scatter plot of the accuracy of the allele frequency estimates from the sequencing compared to the number of variant alleles at HapMap/58BC intersection true positive variants in the Pool of 50 individuals for: (A) PCR, (B) aHC and (C) sHC enrichment. The accuracy of the estimates are calculated as the frequency calculated from the HapMap genotypes minus the frequency estimated from the sequencing data. The y-axis is the accuracy value and the x-axis is the number of variant alleles in the pool. The red line is the least squares fit of the model 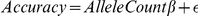, and the corr is the Pearson's correlation coefficient between the accuracy and read depth.(TIF)

Table S1 **Non-indexed pool designs.** This table details the HapMap and 1958BC sample composition of the non-indexed pools of size 2, 10, 20 and 50. The table also details the number of HapMap individuals in each pool that were sequenced in pilot 1 of the 1KG project.
(PDF)

Table S2 **Pool sequencing designs.** This table details the number of lanes sequenced per pool, the read lengths generated per lane, and whether the pool had technical replicates performed.
(PDF)

Table S3 **Target regions for enrichment.** These 6 genomic regions were selected for sequence enrichment on the basis of preliminary rare variant association to Type 2 Diabetes. The target regions include 50 Kb upstream and down stream of the translation start and stop sites for each gene, and include both protein coding (COD) and non-coding (NON-COD) sequence.
(PDF)

Table S4
**Target sequence enrichment success after duplicate removal.** For each pool and sequence enrichment method this table details the total number of reads generated for the pool, the estimated percentage of duplicate reads, the percentage of total reads mapped to the reference genome after duplicate removal, the percentage of total reads mapped to the target regions after duplicate removal, and the percentage of mapped reads that mapped to the target regions with mapping quality 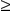
20 after duplicate removal. The total number of reads for a pool is calculated from the fastq file(s) generated for each lane of sequencing. The percentage of reads mapped to the reference is calculated from the BAM file generated from merging all the Maq map files for each lane for a pool. The percentage of reads mapped to the target regions is calculated as the number of reads with at least one base overlapping a target region divided by the total number of reads. The percentage of reads mapped to the target regions with a mapping quality score 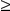
Q20 is calculated as the number of reads with at least one base overlapping a target region with mapping Q
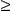
20 divided by the total number of reads.(PDF)

Table S5 **Enrichment success for technical replicates before duplicate removal.** For each technical replicate of the Pool of 20 this table details the total number of reads generated for the pool, the percentage of total reads mapped to the reference genome, the percentage of total reads mapped to the target regions, the percentage of mapped reads that mapped to the target regions, and the median read depth of the target regions. The total number of reads for a pool is calculated from the fastq file(s) generated for each lane of sequencing. The percentage of reads mapped to the reference is calculated from the BAM file generated from merging all the Maq map files for each lane for a pool. The percentage of reads mapped to the target regions is calculated as the number of reads with at least one base overlapping a target region divided by the total number of reads. The percentage of mapped reads mapped to the target is calculated as the number of reads with at least one base overlapping a target region divided by the total number or reads mapped in the BAM file.
(PDF)

Table S6 **Enrichment success for technical replicates after duplicate removal.** For each technical replicate of the Pool of 20 this table details the total number of reads generated for the pool, the percentage of total reads mapped to the reference genome after duplicate removal, the percentage of total reads mapped to the target regions after duplicate removal, the percentage of mapped reads that mapped to the target regions after duplicate removal, and the median read depth of the target regions after duplicate removal. The total number of reads for a pool is calculated from the fastq file(s) generated for each lane of sequencing. The percentage of reads mapped to the reference is calculated from the BAM file generated from merging all the Maq map files for each lane for a pool. The percentage of reads mapped to the target regions is calculated as the number of reads with at least one base overlapping a target region divided by the total number of reads. The percentage of mapped reads mapped to the target is calculated as the number of reads with at least one base overlapping a target region divided by the total number or reads mapped in the BAM file.
(PDF)

Table S7 **Coverage of the target region before duplicate removal.** For each pool and enrichment method this table shows the mean, median and standard deviation of target coverage before duplicate removal. The mean coverage is calculated by summing the read depth for each target base and dividing by the total length of the target regions. The median and standard deviation are calculated from the distribution of read depths for target bases.
(PDF)

Table S8 **Coverage of the target region after duplicate removal.** For each pool and enrichment method this table shows the mean, median and standard deviation of target coverage after duplicate removal. The mean coverage is calculated by summing the read depth for each target base and dividing by the total length of the target regions. The median and standard deviation are calculated from the distribution of read depths for target bases.
(PDF)

Table S9 **Percentage of target region reads that mapped to the coding vs non-coding regions before duplicate removal.** This table gives the percentage of target reads that mapped to the coding (COD) and non-coding (NON-COD) regions before duplicate removal. This table also gives the median read depth in the coding and non-coding target regions
(PDF)

Table S10 **Percentage of target region reads that mapped to the coding vs non-coding regions after duplicate removal.** This table gives the percentage of target reads that mapped to the coding (COD) and non-coding (NON-COD) regions after duplicate removal. This table also gives the median read depth in the coding and non-coding target regions.
(PDF)

Table S11 **Sequence characteristics of non-coding vs coding target regions.** An analysis of the sequence characteristics of the target coding (COD) and non-coding (NON-COD) regions including the repeat content as analyzed by RepeatMasker open 3.2.9.
(PDF)

Table S12 **Total number of variants called by pool and enrichment technique after duplicate removal.** For each pool size and sequence enrichment method this table details the total number of variants called from the sequencing data.
(PDF)

Table S13 **Total number of variants called by pool and enrichment technique before duplicate removal.** For each pool size and sequence enrichment method this table details the total number of variants called from the sequencing data.
(PDF)

Table S14 **dbSNP129 overlap after duplicate removal.** This table contains the percentage of called variants for each pool and enrichment method that are present in the non-redundant dbSNP129.
(PDF)

Table S15 **dbSNP129 overlap before duplicate removal.** This table contains the percentage of called variants for each pool and enrichment method that are present in the non-redundant dbSNP129.
(PDF)

Table S16 **HapMap variation detection sensitivity before duplicate removal.** This table contains the percentage of the known HapMap variants with at least one non-reference allele in the pool that each pool and enrichment method discovered (true positives). The false negative rate is 100 minus this value.
(PDF)

Table S17 **HapMap variation detection specificity before duplicate removal.** This table contains the percentage of the known HapMap variants with no non-reference alleles and no missing genotypes in the pool that each pool and enrichment method correctly didn't call as a variant (true negatives). The false positive rate is 100 minus this value.
(PDF)

Table S18 **1KG support for HapMap false positive loci before duplicate removal.** This table contains the number of loci considered false positives based on HapMap data that are present in 1KG and the percentage of these overlapping loci that the 1KG data supports the presence of non-reference alleles in the pool.
(PDF)

Table S19 **1KG variation detection sensitivity after duplicate removal.** This table contains the percentage of the known 1KG variants with at least one non-reference allele in the pool that each pool and enrichment method discovered (true positives). The false negative rate is 100 minus this value.
(PDF)

Table S20 **1KG variation detection sensitivity before duplicate removal.** This table contains the percentage of the known 1KG variants with at least one non-reference allele in the pool that each pool and enrichment method discovered (true positives). The false negative rate is 100 minus this value.
(PDF)

Table S21 **Total known HapMap/1KG variation detection sensitivity after duplicate removal.** This table contains the percentage of all the known variants with at least one non-reference allele in the pool that each pool and enrichment method discovered (true positives). The false negative rate is 100 minus this value. For individuals that have both 1KG and HapMap data, if a locus occurred in both data sets the HapMap genotype was selected. If a locus occurred in both data sets and an individual's HapMap genotype was missing but called in 1KG, the 1KG genotype was used.
(PDF)

Table S22 **Total known HapMap/1KG variation detection sensitivity before duplicate removal.** This table contains the percentage of all the known variants with at least one non-reference allele in the pool that each pool and enrichment method discovered (true positives). The false negative rate is 100 minus this value. For individuals that have both 1KG and HapMap data, if a locus occurred in both data sets the HapMap genotype was selected. If a locus occurred in both data sets and an individual's HapMap genotype was missing but called in 1KG, the 1KG genotype was used.
(PDF)

Table S23 **HapMap singleton detection sensitivity after duplicate removal.** This table illustrates the ability of the sequencing based variant calling to identify variants where the HapMap genotypes have a single non-reference or reference base. The only loci analyzed here are those where there are no missing genotypes for pooled individuals.
(PDF)

Table S24 **Variation detection sensitivity as pool size grows after duplicate removal.** This table shows the percentage of the variants called in the pool of 1 individual that are also called as variants in the larger pool sizes. The individual in the pool of 1 was also in each subsequent larger pool, therefore all variants called in the pool of 1 should also be found in all subsequent pools.
(PDF)

Table S25 **Variation detection sensitivity as pool size grows before duplicate removal.** This table shows the percentage of the variants called in the pool of 1 individual that are also called as variants in the larger pool sizes. The individual in the pool of 1 was also in each subsequent larger pool, therefore all variants called in the pool of 1 should also be found in all subsequent pools.
(PDF)

Table S26 **Percent of called HapMap variants with correctly, under, and over estimated non-reference allele frequencies after duplicate removal.** For the pools of 10, 20 and 50 individuals and each enrichment method this table details the percent of true positive variants that the non-reference allele frequency was correctly, under, or over estimated by the sequencing based variant caller relative to the reference genotypes.
(PDF)

Table S27 **Percent of called HapMap variants with correctly, under, and over estimated non-reference allele frequencies before duplicate removal.** For the pools of 10, 20 and 50 individuals and each enrichment method this table details the percent of true positive variants that the non-reference allele frequency was correctly, under, or over estimated by the sequencing based variant caller relative to the reference genotypes.
(PDF)

Table S28 **Pool of 20 technical replicates dbSNP overlap and HapMap/1KG sensitivity after duplicate removal.** This table contains the percentage of the called variants in dbSNP129, and the percentage of known HapMap/1KG variants with at least one non-reference allele in the pool that each replicate discovered (true positives). The false negative rate is 100 minus this value.
(PDF)

Table S29 **Pool of 20 technical replicate variant overlap after duplicate removal.** For the PCR and aHC technical replicates for the pool of 20 this table details the total number of variants called for each replicate, the number of variants called by both replicates, the percent overlap of the called variants in the replicates, the average absolute difference in non-reference allele frequency for the overlapping variants, and the Pearson's correlation coefficient for the non-reference allele frequency estimates between the replicates. The average absolute difference is calculated as the sum of the absolute value of the difference in non-reference allele frequency, divided by the total number of sites.
(PDF)
