# Supplementary figures and images for: An Evaluation of Different Target Enrichment Methods in Pooled Sequencing Designs for Complex Disease Association Studies
Source: PLoS One. 2011 Nov 1;6(11):e26279. doi: 10.1371/journal.pone.0026279 (PMC3206031; doi:10.1371/journal.pone.0026279)

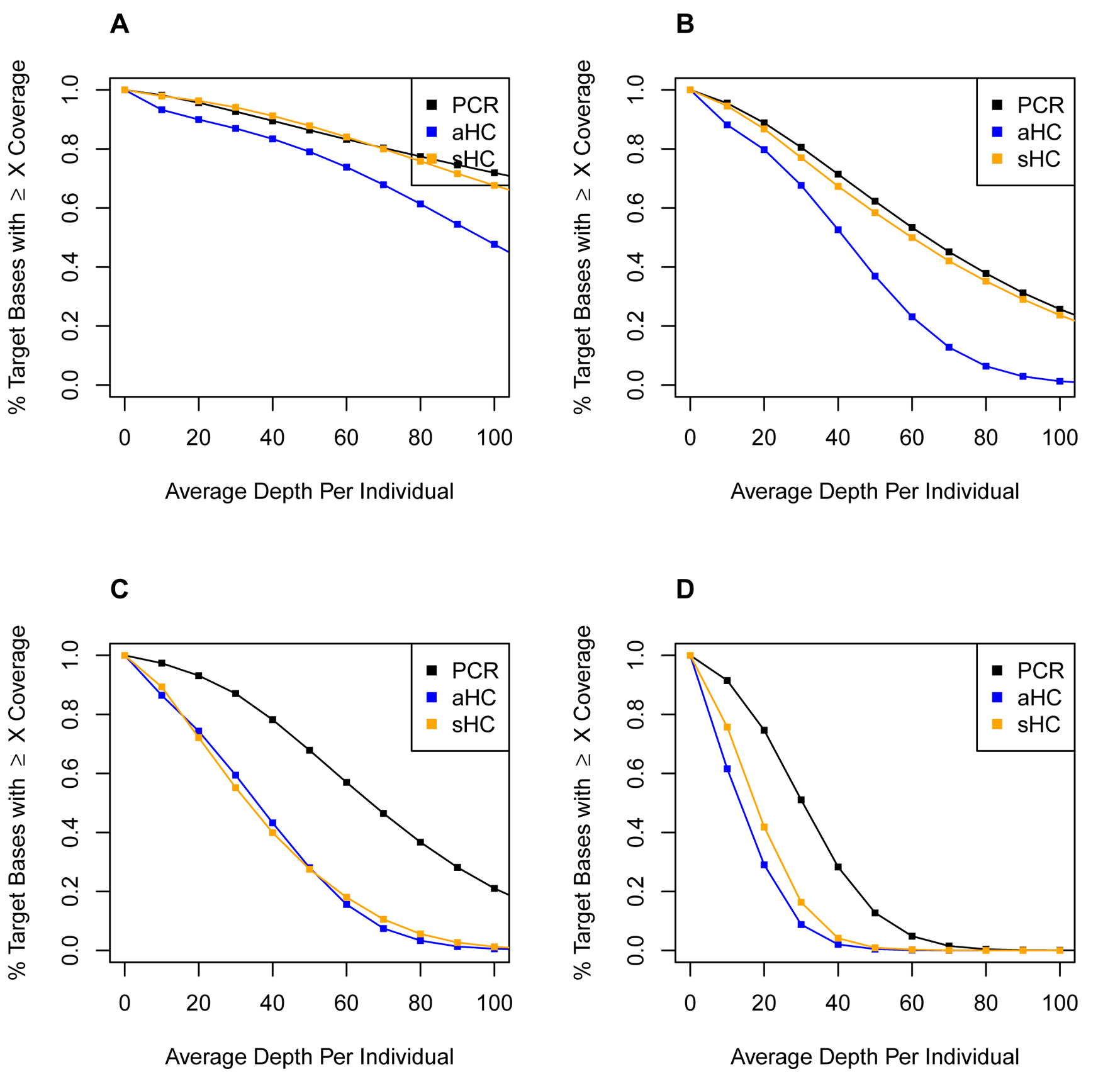

Supplement: Figure S1 — Target coverage per individual in pool after duplicate removal. This shows a cumulative relative frequency plot of the percentage of target bases with X coverage depth normalized by the number of individuals sequenced for: (A) Pool of 2, (B) Pool of 10, (C) Pool of 20 and (D) Pool of 50 individuals. The x-axis is in increments of 10× coverage. The black squares/lines illustrate the data for PCR enrichment, the blue squares/lines illustrate the data for aHC enrichment and the orange squares/lines illustrate the data for sHC enrichment. The first square represents the percentage of target bases with 10× coverage per individual in the pool, and so on for each square in increments of 10×. This analysis assumes equal representation of each individual in the pool of DNA. (TIF) [file pone.0026279.s001.tif]

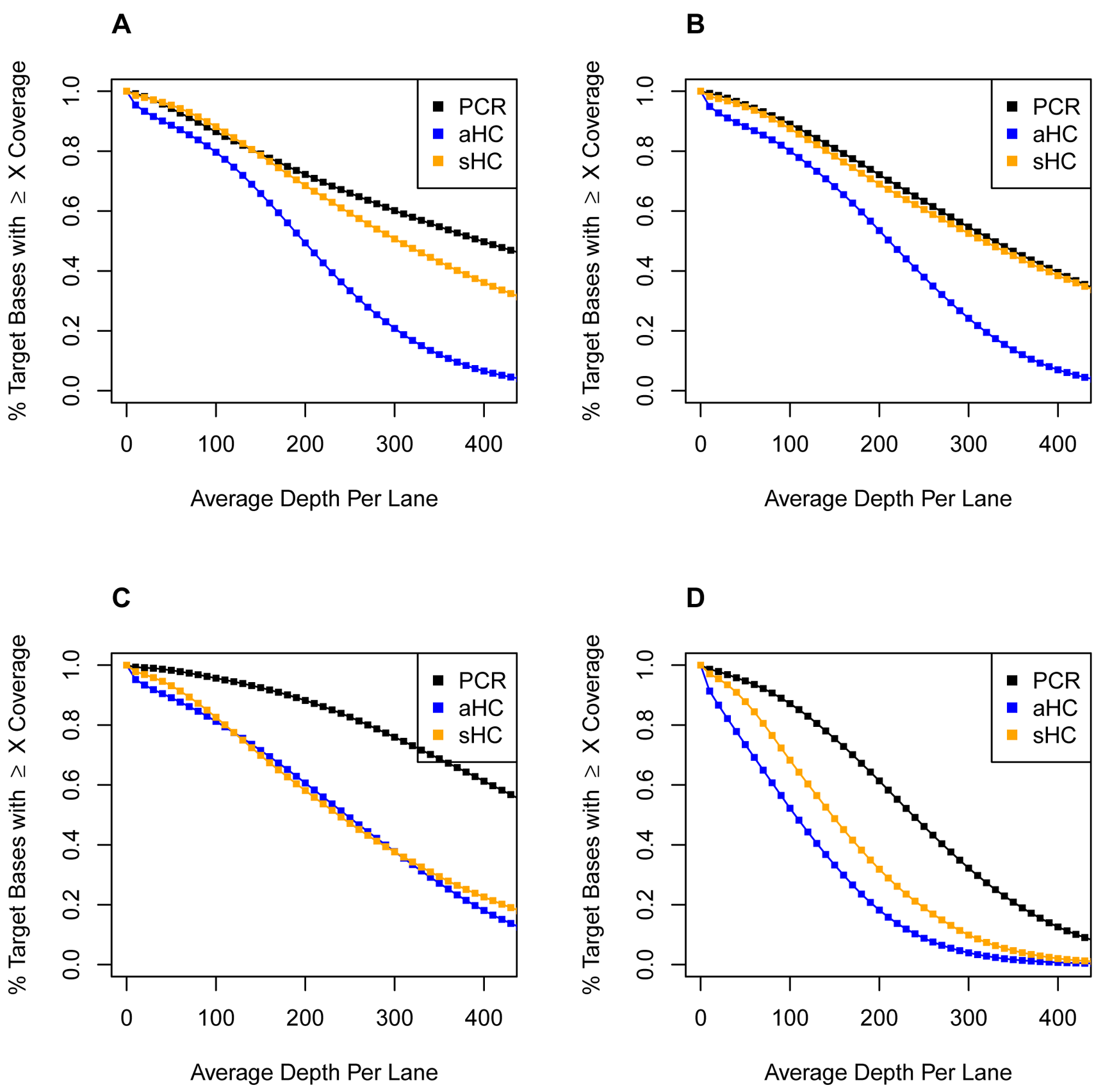

Supplement: Figure S2 — Target coverage per lane of sequencing before duplicate removal. This shows a cumulative relative frequency plot of the percentage of target bases with X coverage depth normalized by the number of lanes sequenced for: (A) Pool of 2, (B) Pool of 10, (C) Pool of 20 and (D) Pool of 50 individuals. The x-axis is in increments of 10× coverage. The black squares/lines illustrate the data for PCR enrichment, the blue squares/lines illustrate the data for aHC enrichment and the orange squares/lines illustrate the data for sHC enrichment. The first square represents the percentage of target bases with 10× coverage per lane sequenced, and so on for each square in increments of 10×. (TIF) [file pone.0026279.s002.tif]

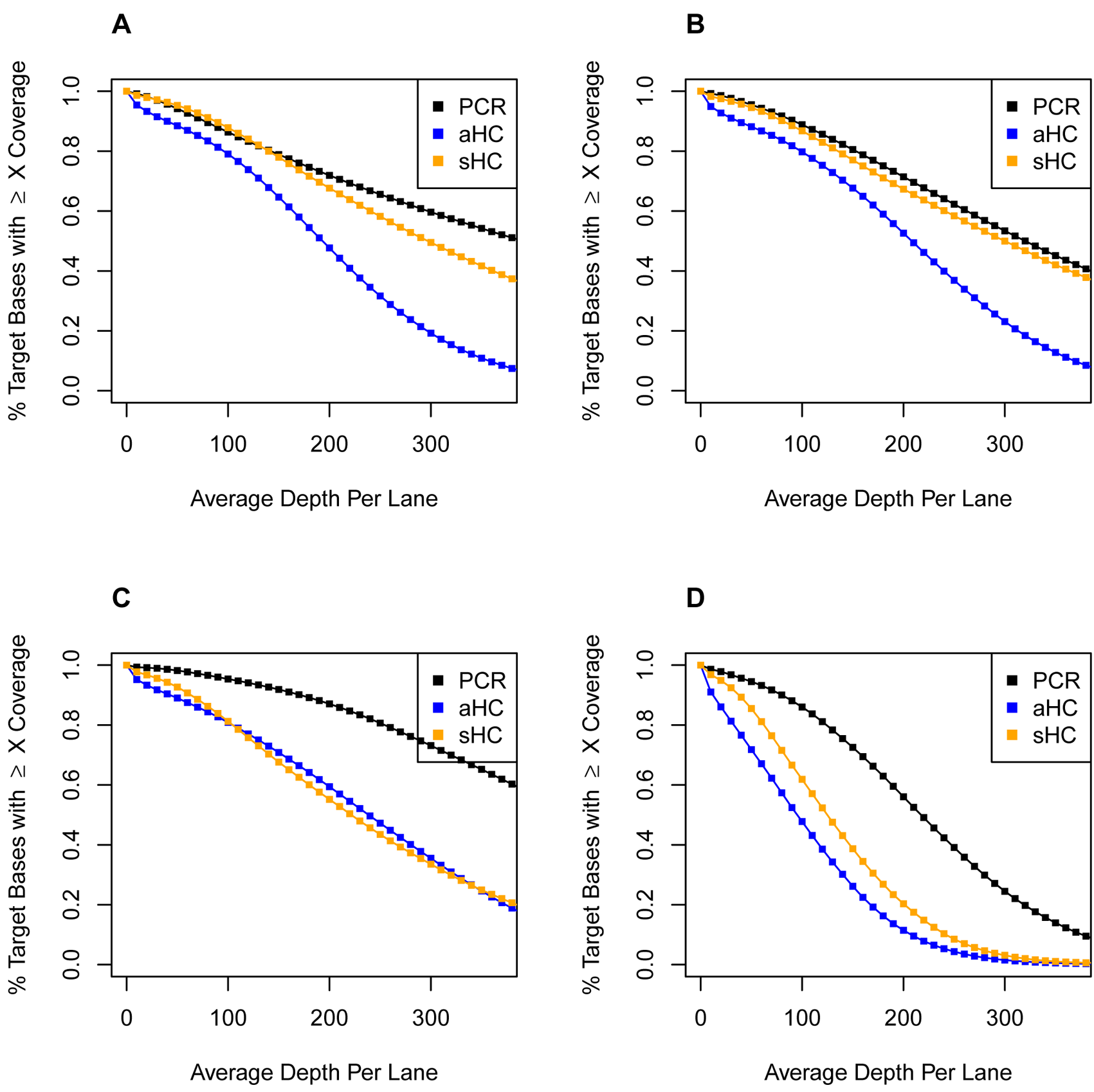

Supplement: Figure S3 — Target coverage per lane of sequencing after duplicate removal. This shows a cumulative relative frequency plot of the percentage of target bases with X coverage depth normalized by the number of lanes sequenced after duplicate removal for: (A) Pool of 2, (B) Pool of 10, (C) Pool of 20 and (D) Pool of 50 individuals. The x-axis is in increments of 10× coverage. The black squares/lines illustrate the data for PCR enrichment, the blue squares/lines illustrate the data for aHC enrichment and the orange squares/lines illustrate the data for sHC enrichment. The first square represents the percentage of target bases with 10× coverage per lane sequenced, and so on for each square in increments of 10×. (TIF) [file pone.0026279.s003.tif]

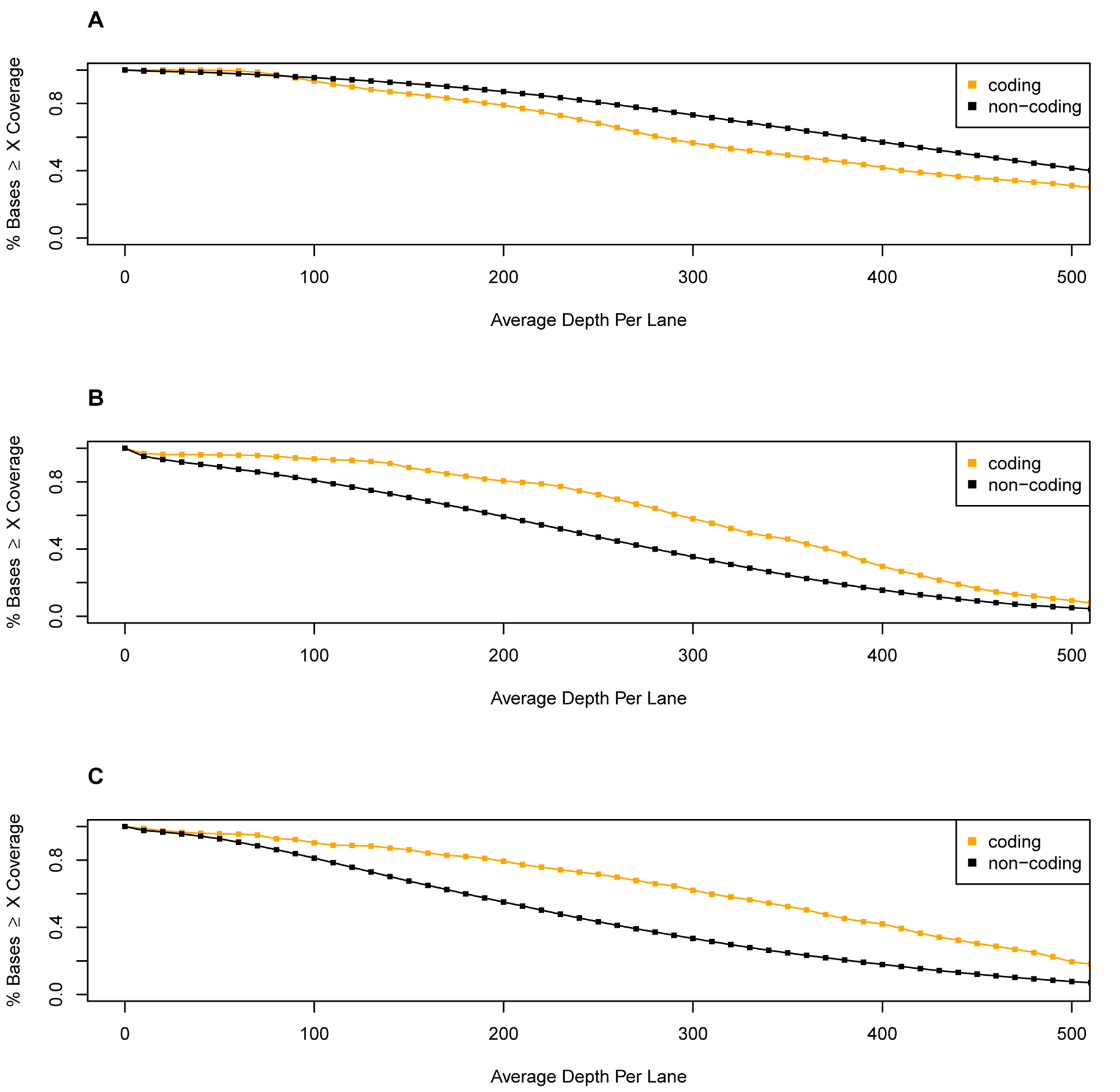

Supplement: Figure S4 — Pool of 20 coding vs. non-coding target coverage per lane after duplicate removal. This figure shows a cumulative relative frequency plot of the percentage of target bases with X coverage depth normalized by the number of lanes sequenced after duplicate removal for the Pool of 20 individuals for: (A) PCR, (B) aHC and (C) sHC enrichment. The orange squares/lines illustrate the data for protein coding target bases and the black squares/lines illustrate the data for the non-coding target bases. The first square represents the percentage of target bases with 10× coverage per lane in the pool, and so on for each square in increments of 10×. (TIF) [file pone.0026279.s004.tif]

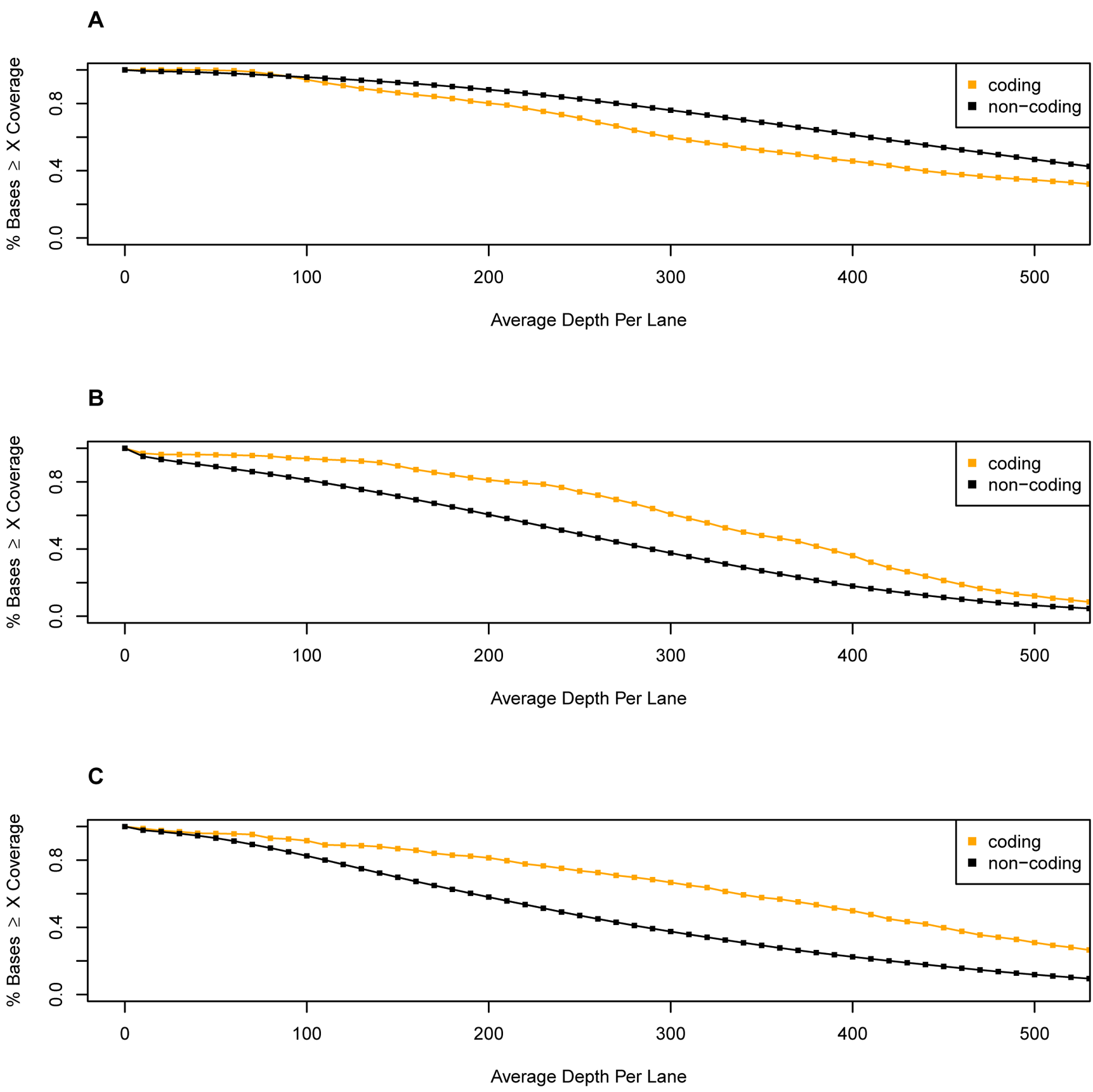

Supplement: Figure S5 — Pool of 20 coding vs. non-coding target coverage per lane before duplicate removal. This figure shows a cumulative relative frequency plot of the percentage of target bases with X coverage depth normalized by the number of lanes sequenced for the Pool of 20 individuals for: (A) PCR, (B) aHC and (C) sHC enrichment. The orange squares/lines illustrate the data for protein coding target bases and the black squares/lines illustrate the data for the non-coding target bases. The first square represents the percentage of target bases with 10× coverage per lane in the pool, and so on for each square in increments of 10×. (TIF) [file pone.0026279.s005.tif]

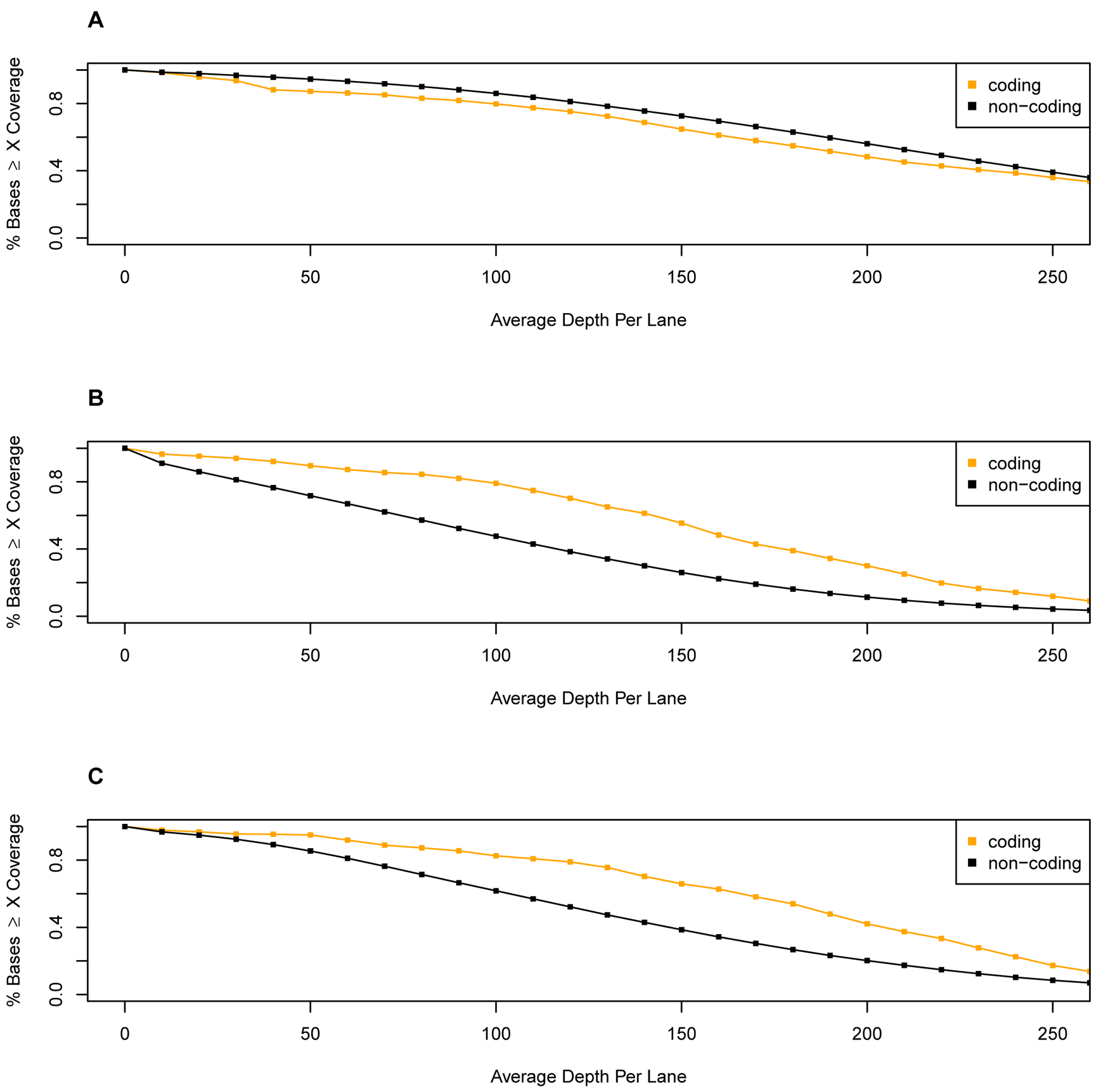

Supplement: Figure S6 — Pool of 50 coding vs. non-coding target coverage per lane after duplicate removal. This figure shows a cumulative relative frequency plot of the percentage of target bases with X coverage depth normalized by the number of lanes sequenced after duplicate removal for the Pool of 50 individuals for: (A) PCR, (B) aHC and (C) sHC enrichment. The orange squares/lines illustrate the data for protein coding target bases and the black squares/lines illustrate the data for the non-coding target bases. The first square represents the percentage of target bases with 10× coverage per lane in the pool, and so on for each square in increments of 10×. (TIF) [file pone.0026279.s006.tif]

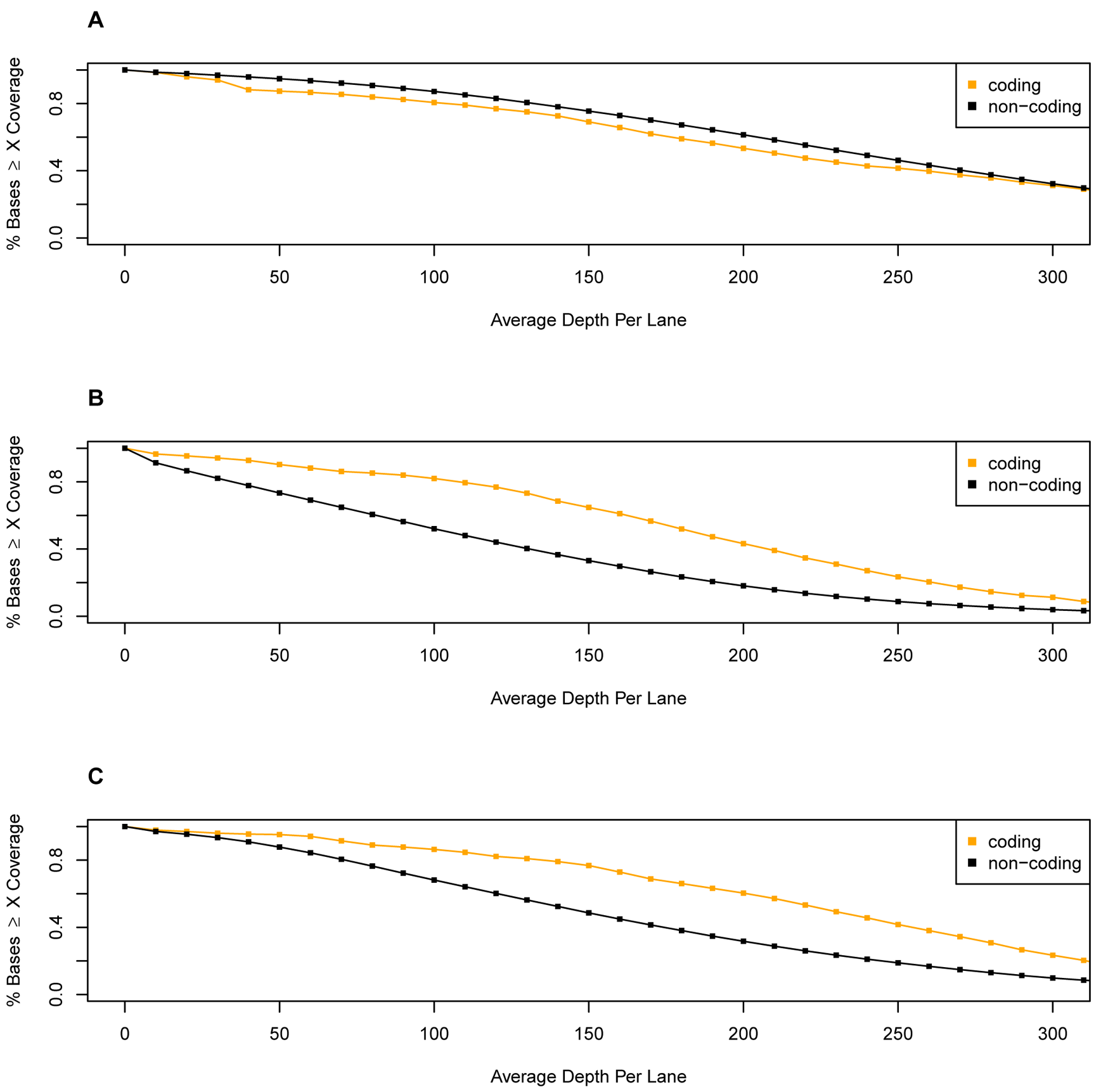

Supplement: Figure S7 — Pool of 50 coding vs. non-coding target coverage per lane before duplicate removal. This figure shows a cumulative relative frequency plot of the percentage of target bases with X coverage depth normalized by the number of lanes sequenced for the Pool of 50 individuals for: (A) PCR, (B) aHC and (C) sHC enrichment. The orange squares/lines illustrate the data for protein coding target bases and the black squares/lines illustrate the data for the non-coding target bases. The first square represents the percentage of target bases with 10× coverage per lane in the pool, and so on for each square in increments of 10×. (TIF) [file pone.0026279.s007.tif]

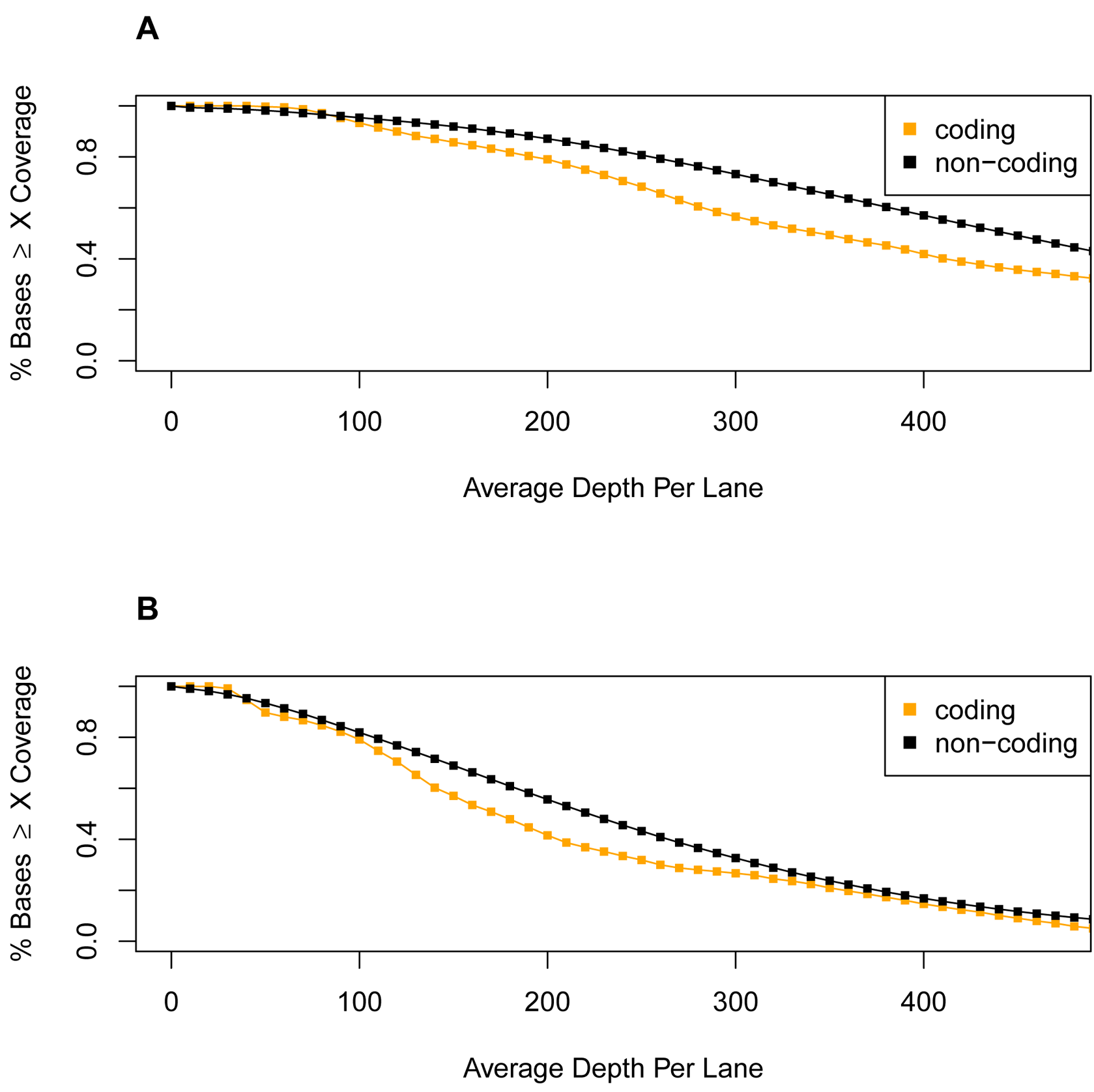

Supplement: Figure S8 — Pool of 20 PCR replicates coding vs. non-coding target coverage per lane after duplicate removal. This figure shows a cumulative relative frequency plot of the percentage of target bases with X coverage depth normalized by the number of lanes sequenced for the Pool of 20 individuals PCR replicates for: (A) Replicate 1, (B) Replicate 2. Replicate 1 is the replicate used in all the main analyses. The orange squares/lines illustrate the data for protein coding target bases and the black squares/lines illustrate the data for the non-coding target bases. The first square represents the percentage of target bases with 10× coverage per lane in the pool, and so on for each square in increments of 10×. (TIF) [file pone.0026279.s008.tif]

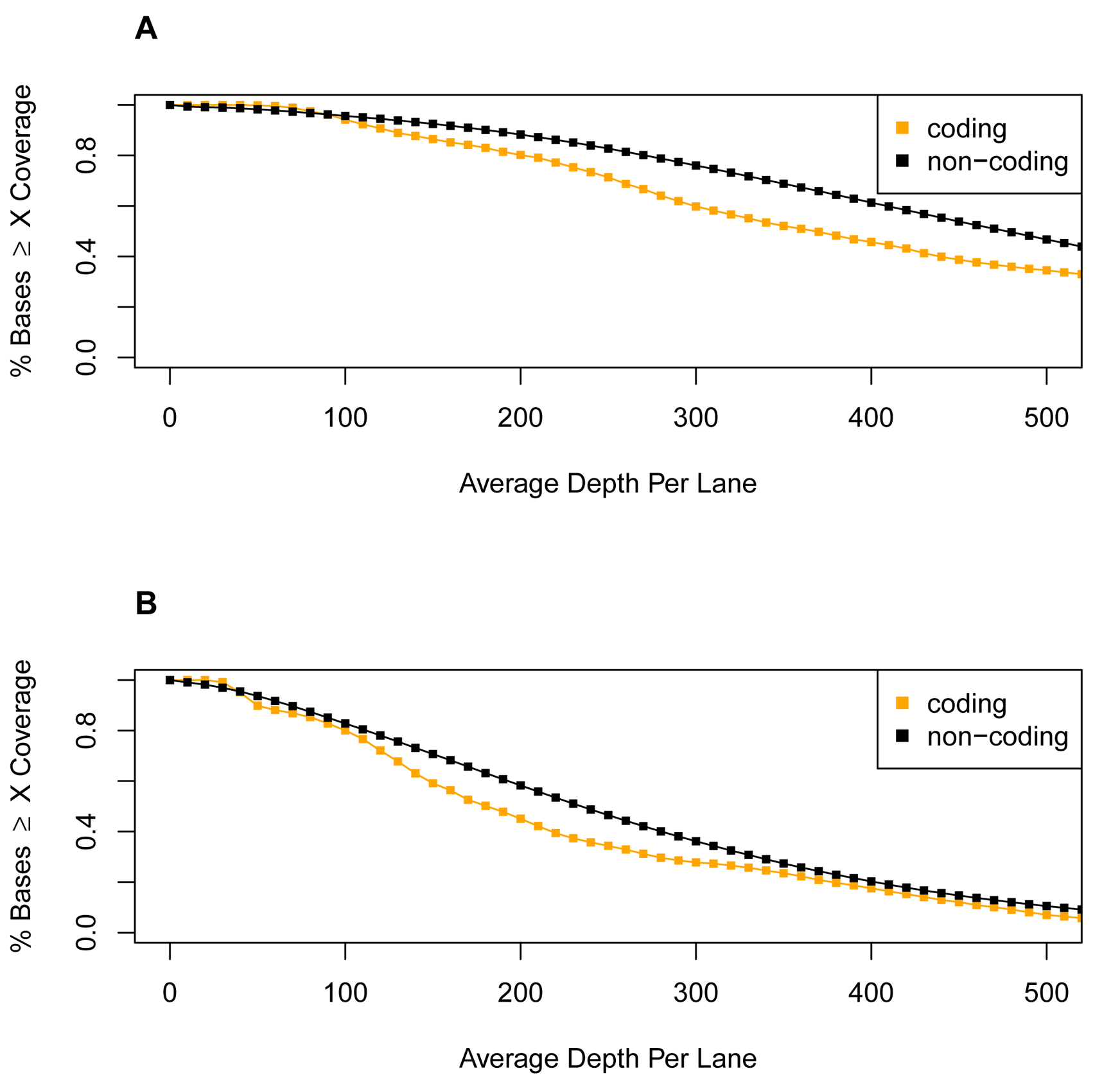

Supplement: Figure S9 — Pool of 20 PCR replicates coding vs. non-coding target coverage per lane before duplicate removal. This figure shows a cumulative relative frequency plot of the percentage of target bases with X coverage depth normalized by the number of lanes sequenced for the Pool of 20 individuals PCR replicates for: (A) Replicate 1, (B) Replicate 2. Replicate 1 is the replicate used in all the main analyses. The orange squares/lines illustrate the data for protein coding target bases and the black squares/lines illustrate the data for the non-coding target bases. The first square represents the percentage of target bases with 10× coverage per lane in the pool, and so on for each square in increments of 10×. (TIF) [file pone.0026279.s009.tif]

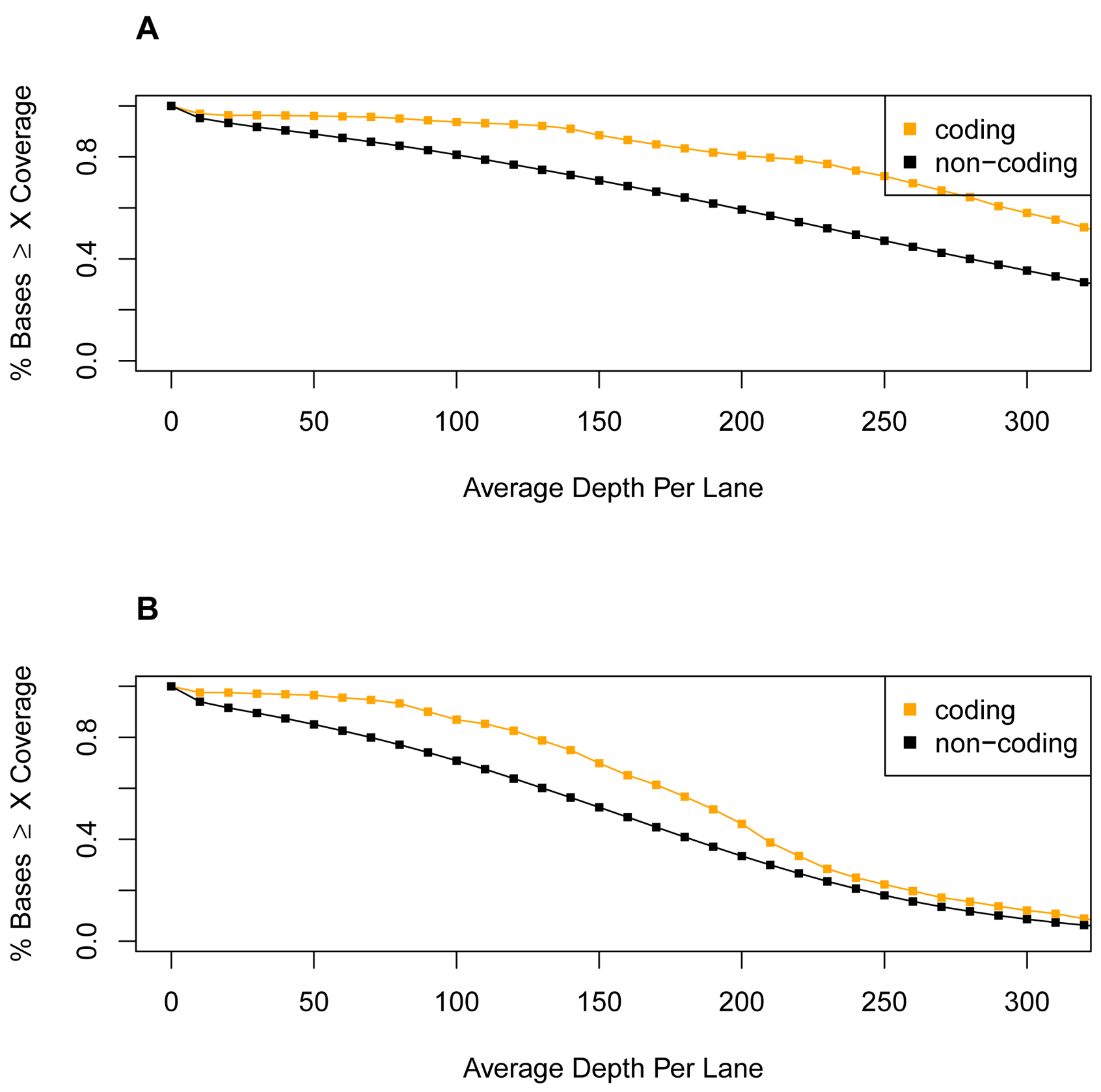

Supplement: Figure S10 — Pool of 20 aHC replicates coding vs. non-coding target coverage per lane after duplicate removal. This figure shows a cumulative relative frequency plot of the percentage of target bases with X coverage depth normalized by the number of lanes sequenced for the Pool of 20 individuals aHC replicates for: (A) Replicate 1, (B) Replicate 2. Replicate 1 is the replicate used in all the main analyses. The orange squares/lines illustrate the data for protein coding target bases and the black squares/lines illustrate the data for the non-coding target bases. The first square represents the percentage of target bases with 10× coverage per lane in the pool, and so on for each square in increments of 10×. (TIF) [file pone.0026279.s010.tif]

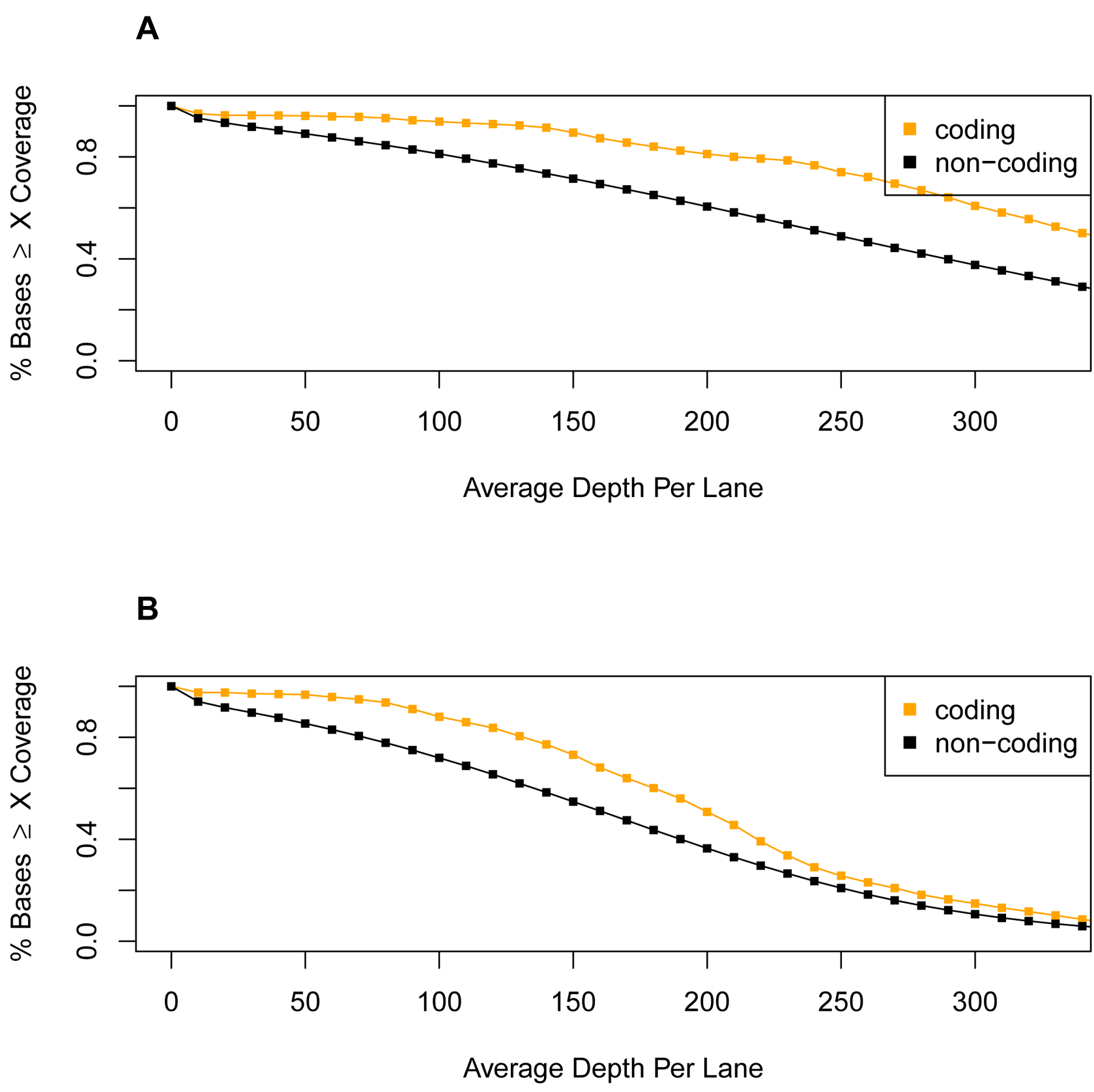

Supplement: Figure S11 — Pool of 20 aHC replicates coding vs. non-coding target coverage per lane before duplicate removal. This figure shows a cumulative relative frequency plot of the percentage of target bases with X coverage depth normalized by the number of lanes sequenced for the Pool of 20 individuals aHC replicates for: (A) Replicate 1, (B) Replicate 2. Replicate 1 is the replicate used in all the main analyses. The orange squares/lines illustrate the data for protein coding target bases and the black squares/lines illustrate the data for the non-coding target bases. The first square represents the percentage of target bases with 10× coverage per lane in the pool, and so on for each square in increments of 10×. (TIF) [file pone.0026279.s011.tif]

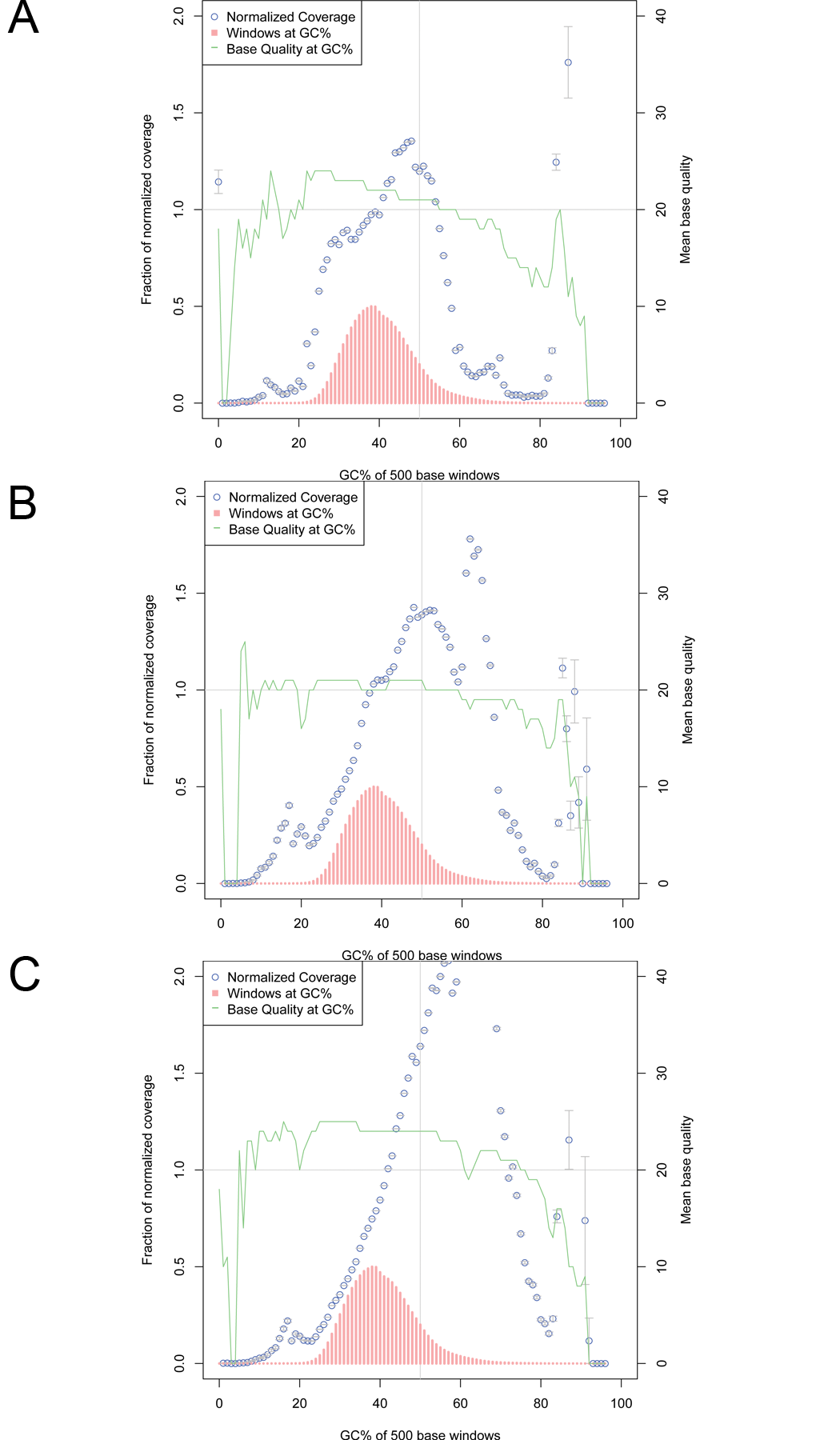

Supplement: Figure S12 — Pool of 20 genomic coverage as function of %GC of reference after duplicate removal. This figure analyzes the normalized coverage and mean base quality of mapped bases compared to the percentage of GC bases for the reference genome divided into 500 base-pair windows in the Pool of 20 individuals for: (A) PCR, (B) aHC and (C) sHC enrichment. Normalized coverage for a %GC bin is the proportion of coverage this window accounts for relative to the mean coverage across all %GC bins. (TIF) [file pone.0026279.s012.tif]

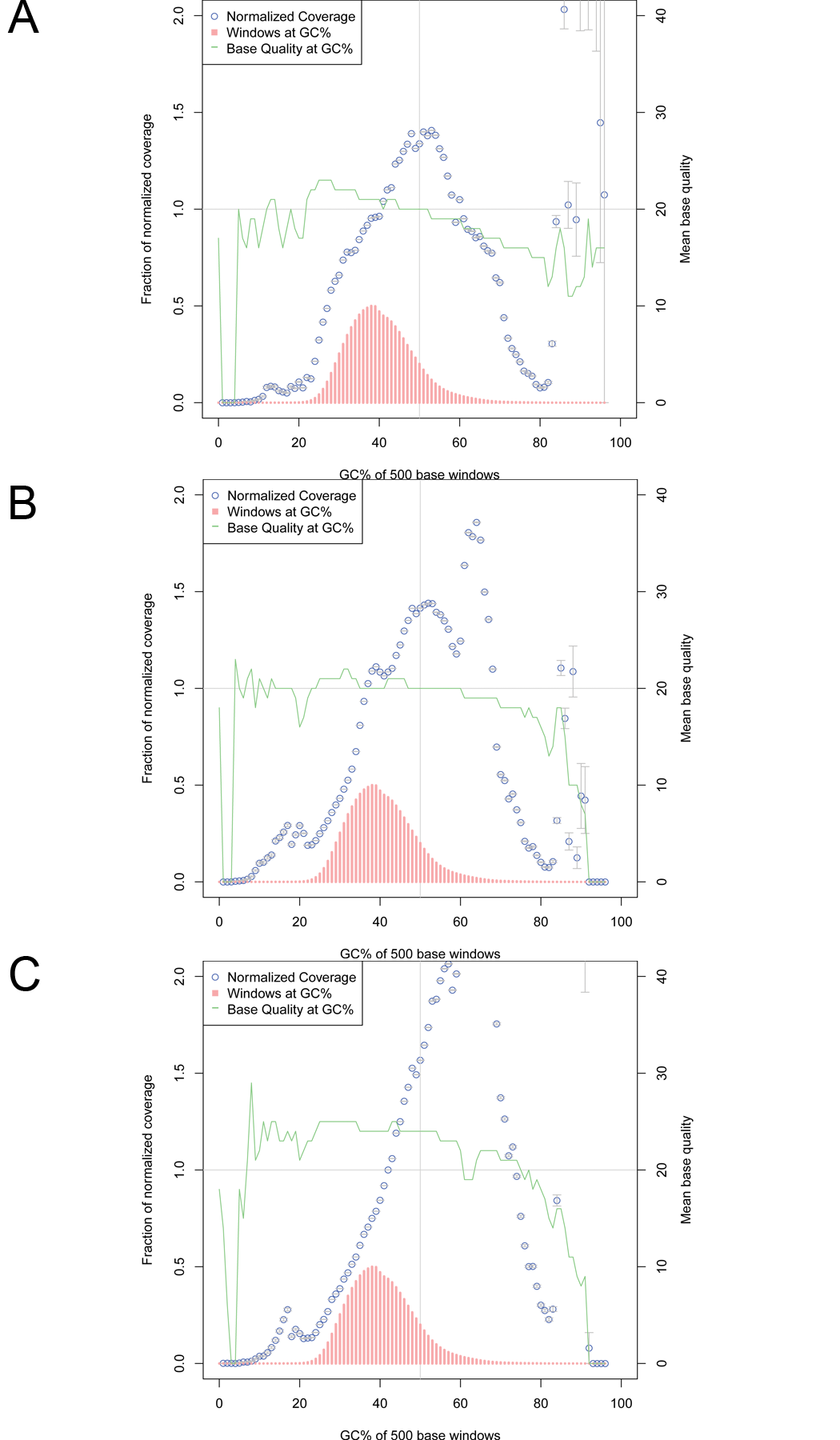

Supplement: Figure S13 — Pool of 50 genomic coverage as function of %GC of reference after duplicate removal. This figure analyzes the normalized coverage and mean base quality of mapped bases compared to the percentage of GC bases for the reference genome divided into 500 base-pair windows in the Pool of 50 individuals for: (A) PCR, (B) aHC and (C) sHC enrichment. Normalized coverage for a %GC bin is the proportion of coverage this window accounts for relative to the mean coverage across all %GC bins. (TIF) [file pone.0026279.s013.tif]

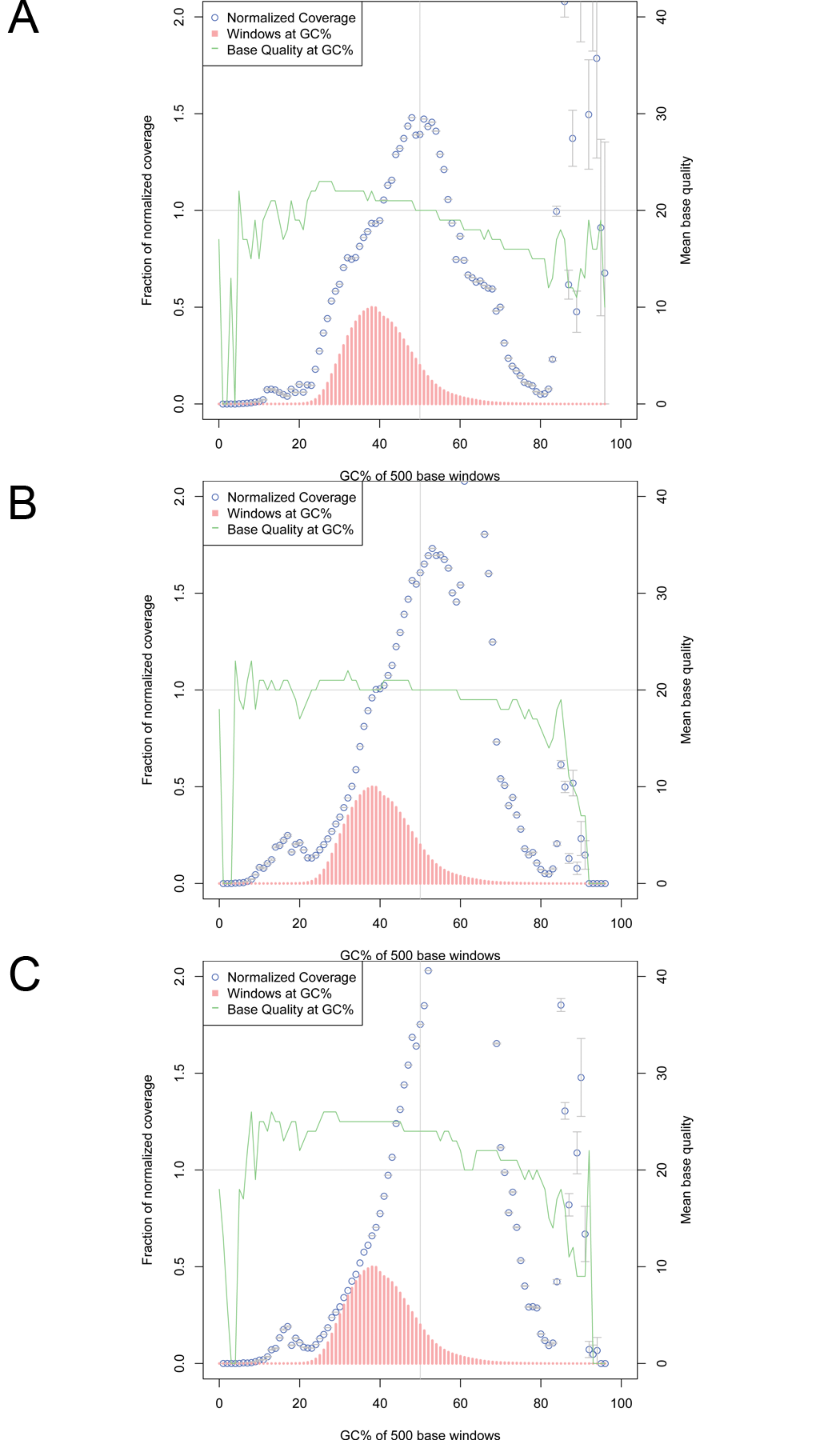

Supplement: Figure S14 — Pool of 50 genomic coverage as function of %GC of reference before duplicate removal. This figure analyzes the normalized coverage and mean base quality of mapped bases compared to the percentage of GC bases for the reference genome divided into 500 base-pair windows in the Pool of 50 individuals for: (A) PCR, (B) aHC and (C) sHC enrichment. Normalized coverage for a %GC bin is the proportion of coverage this window accounts for relative to the mean coverage across all %GC bins. (TIF) [file pone.0026279.s014.tif]

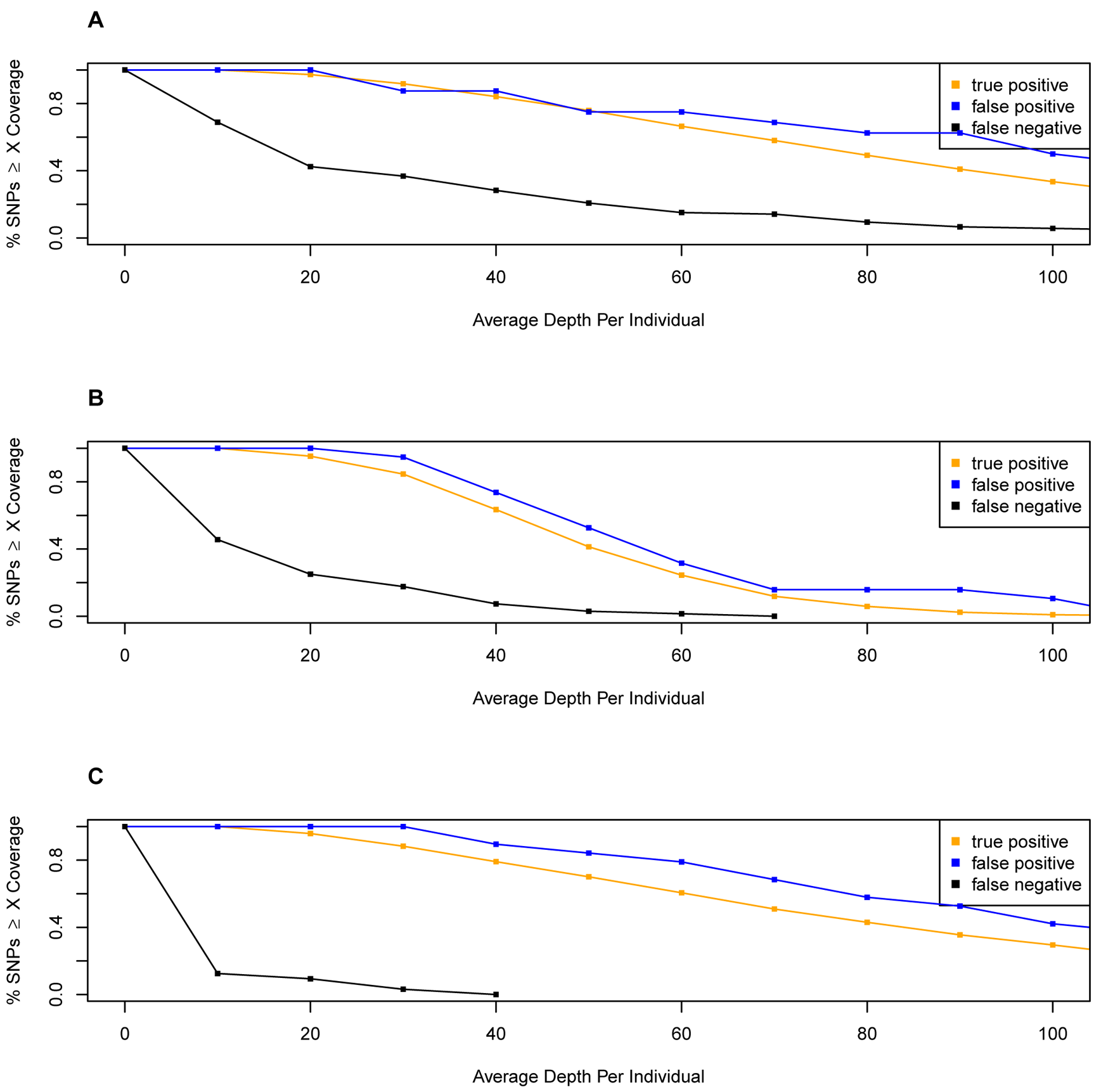

Supplement: Figure S15 — Pool of 10 per individual coverage at HapMap true positive, false positive and false negative variants after duplicate removal. This figure shows a cumulative relative frequency plot of the percentage of variants with X coverage per individual in the pool at HapMap true positive, false positive and false negative variants for: (A) PCR, (B) aHC and (C) sHC enrichment. The black squares/lines illustrate the data for false negative variants, the blue squares/lines illustrate the data for false positive variants and the orange squares/lines illustrate the data for true positive variants. The first square represents the percentage of variants in a class with 10× coverage per individual in the pool, and so on for each square in increments of 10×. (TIF) [file pone.0026279.s015.tif]

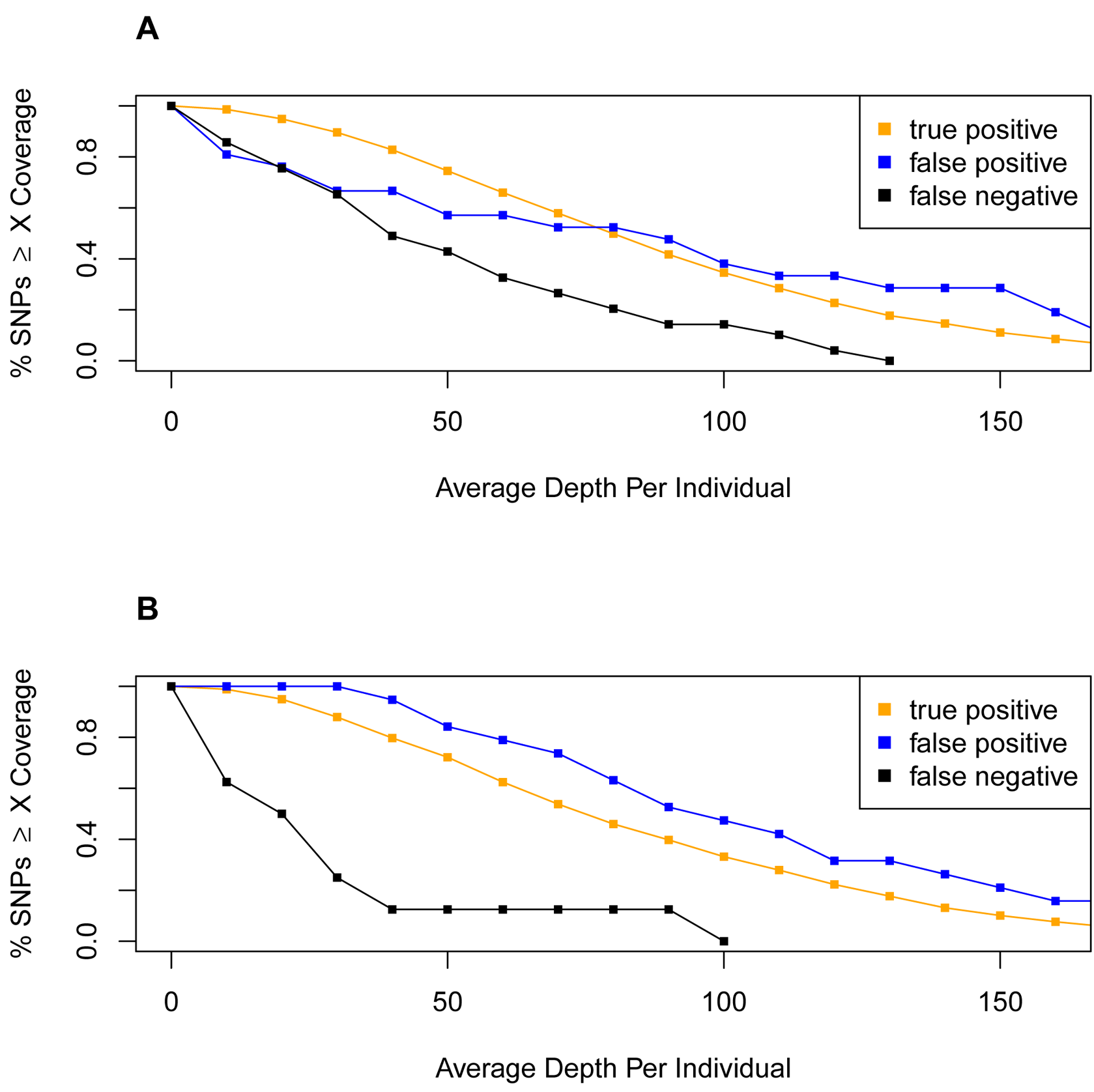

Supplement: Figure S16 — Pool of 10 per individual coverage at HapMap true positive, false positive and false negative variants before duplicate removal. This figure shows a cumulative relative frequency plot of the percentage of variants with X coverage per individual in the pool at HapMap true positive, false positive and false negative variants for: (A) PCR and (B) sHC enrichment. The black squares/lines illustrate the data for false negative variants, the blue squares/lines illustrate the data for false positive variants and the orange squares/lines illustrate the data for true positive variants. The first square represents the percentage of variants in a class with 10× coverage per individual in the pool, and so on for each square in increments of 10×. (TIF) [file pone.0026279.s016.tif]

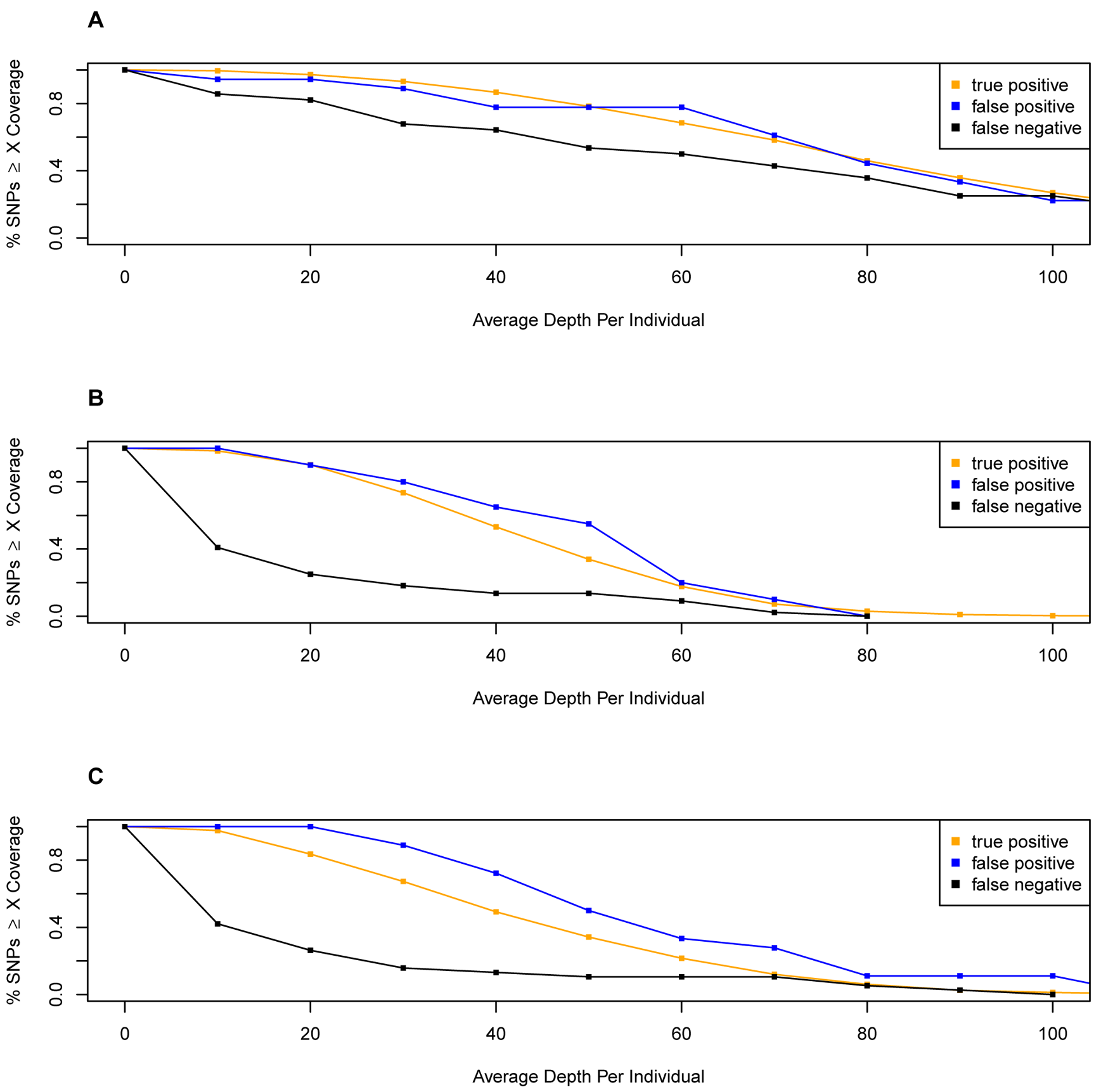

Supplement: Figure S17 — Pool of 20 per individual coverage at HapMap true positive, false positive and false negative variants after duplicate removal. This figure shows a cumulative relative frequency plot of the percentage of variants with X coverage per individual in the pool at HapMap true positive, false positive and false negative variants for: (A) PCR, (B) aHC and (C) sHC enrichment. The black squares/lines illustrate the data for false negative variants, the blue squares/lines illustrate the data for false positive variants and the orange squares/lines illustrate the data for true positive variants. The first square represents the percentage of variants in a class with 10× coverage per individual in the pool, and so on for each square in increments of 10×. (TIF) [file pone.0026279.s017.tif]

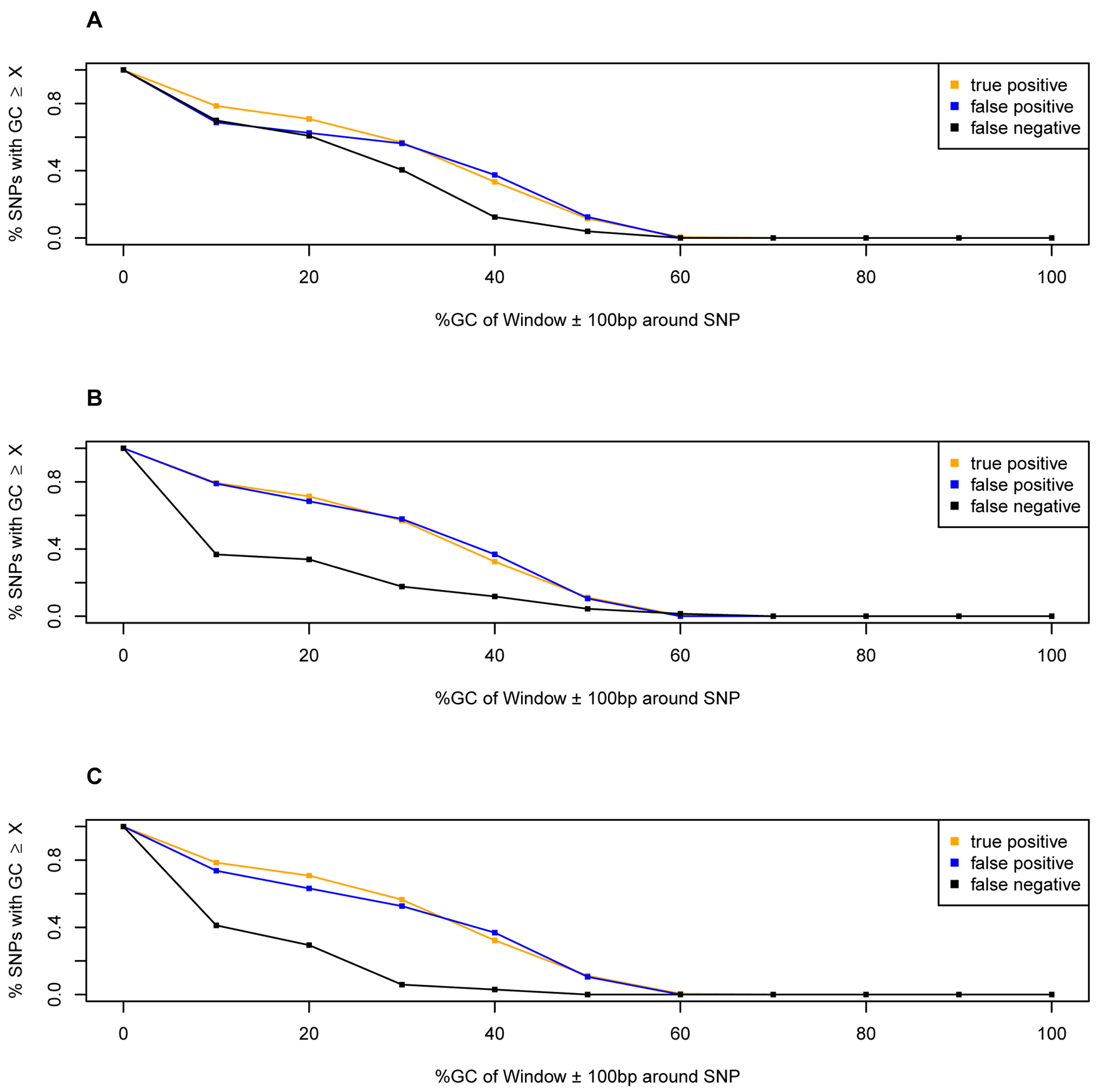

Supplement: Figure S18 — Pool of 10 %GC context at HapMap true positive, false positive and false negative variants after duplicate removal. This figure shows a cumulative relative frequency plot of the percentage of variants with a genomic context %GC of X% in a window of 100 base-pairs around each HapMap true positive, false positive and false negative variants for: (A) PCR, (B) aHC and (C) sHC enrichment. The black squares/lines illustrate the data for false negative variants, the blue squares/lines illustrate the data for false positive variants and the orange squares/lines illustrate the data for true positive variants. The first square represents the percentage of variants in a class with 10% GC in a 100 base-pair window around a variant coverage, and so on for each square in increments of 10% GC content. (TIF) [file pone.0026279.s018.tif]

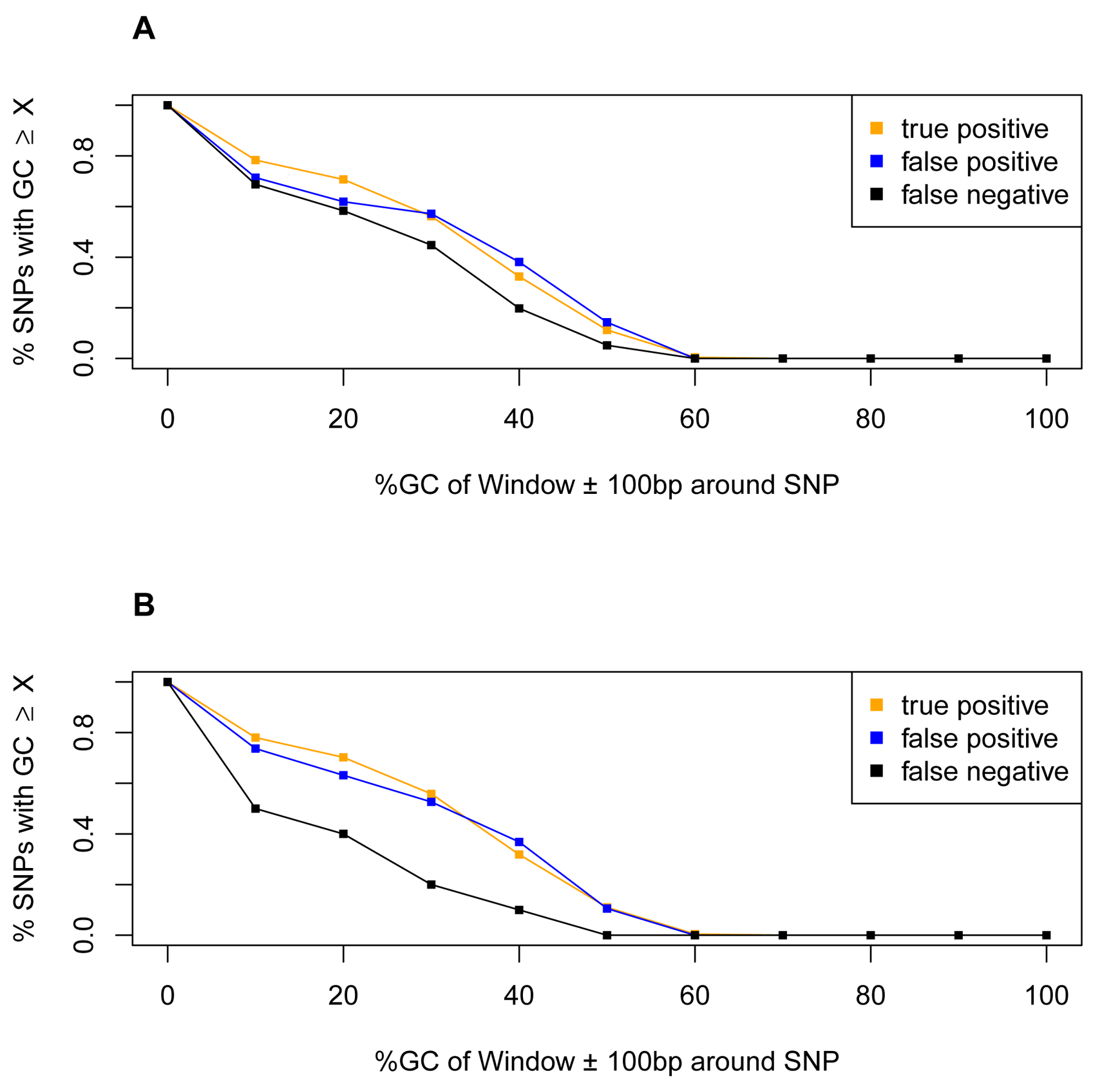

Supplement: Figure S19 — Pool of 10 %GC context at HapMap true positive, false positive and false negative variants before duplicate removal. This figure shows a cumulative relative frequency plot of the percentage of variants with a genomic context %GC of X% in a window of 100 base-pairs around each HapMap true positive, false positive and false negative variants for: (A) PCR and (B) sHC enrichment. The black squares/lines illustrate the data for false negative variants, the blue squares/lines illustrate the data for false positive variants and the orange squares/lines illustrate the data for true positive variants. The first square represents the percentage of variants in a class with 10% GC in a 100 base-pair window around a variant coverage, and so on for each square in increments of 10% GC content. (TIF) [file pone.0026279.s019.tif]

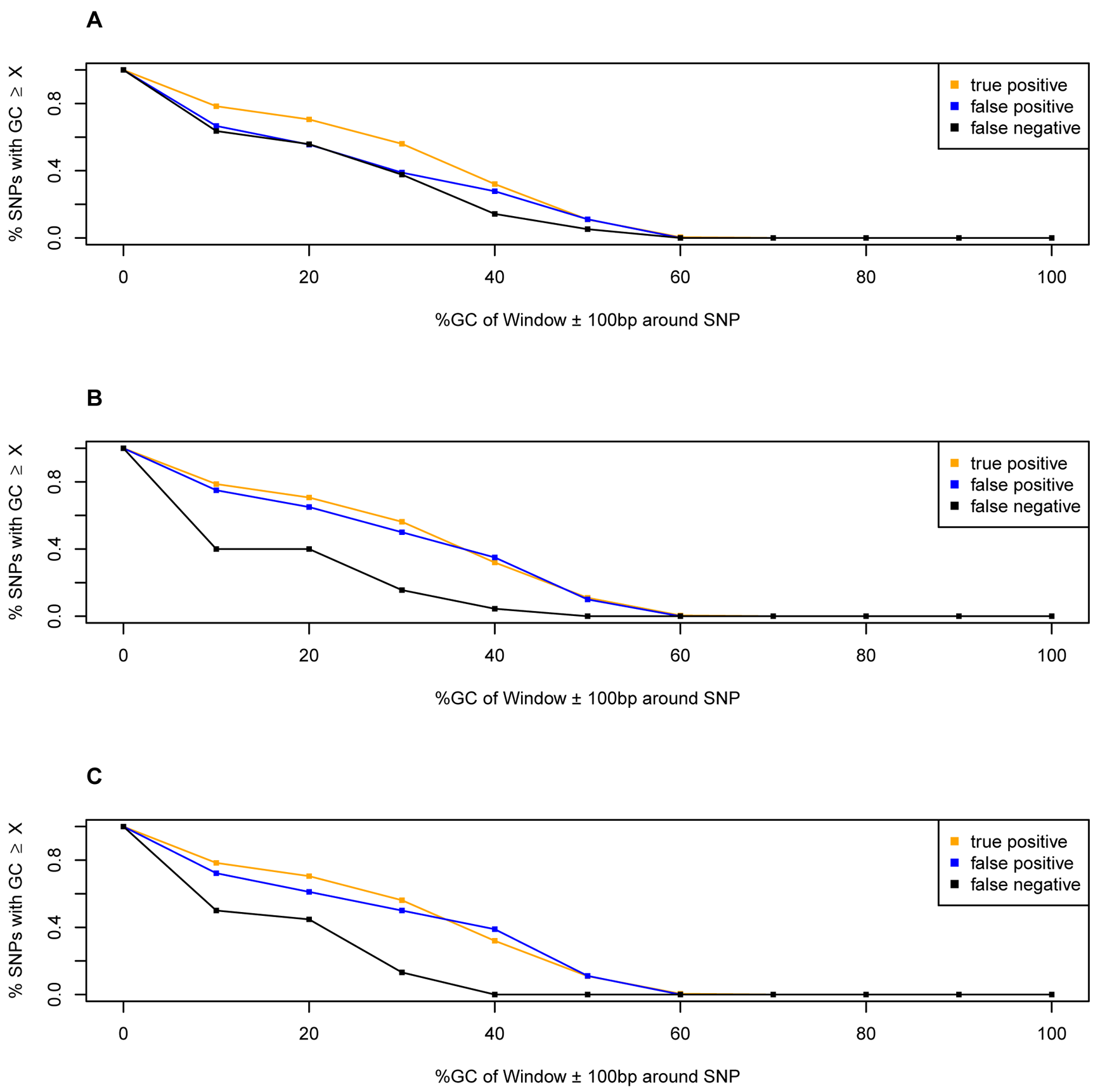

Supplement: Figure S20 — Pool of 20 %GC context at HapMap true positive, false positive and false negative variants after duplicate removal. This figure shows a cumulative relative frequency plot of the percentage of variants with a genomic context %GC of X% in a window of 100 base-pairs around each HapMap true positive, false positive and false negative variants for: (A) PCR, (B) aHC and (C) sHC enrichment. The black squares/lines illustrate the data for false negative variants, the blue squares/lines illustrate the data for false positive variants and the orange squares/lines illustrate the data for true positive variants. The first square represents the percentage of variants in a class with 10% GC in a 100 base-pair window around a variant coverage, and so on for each square in increments of 10% GC content. (TIF) [file pone.0026279.s020.tif]

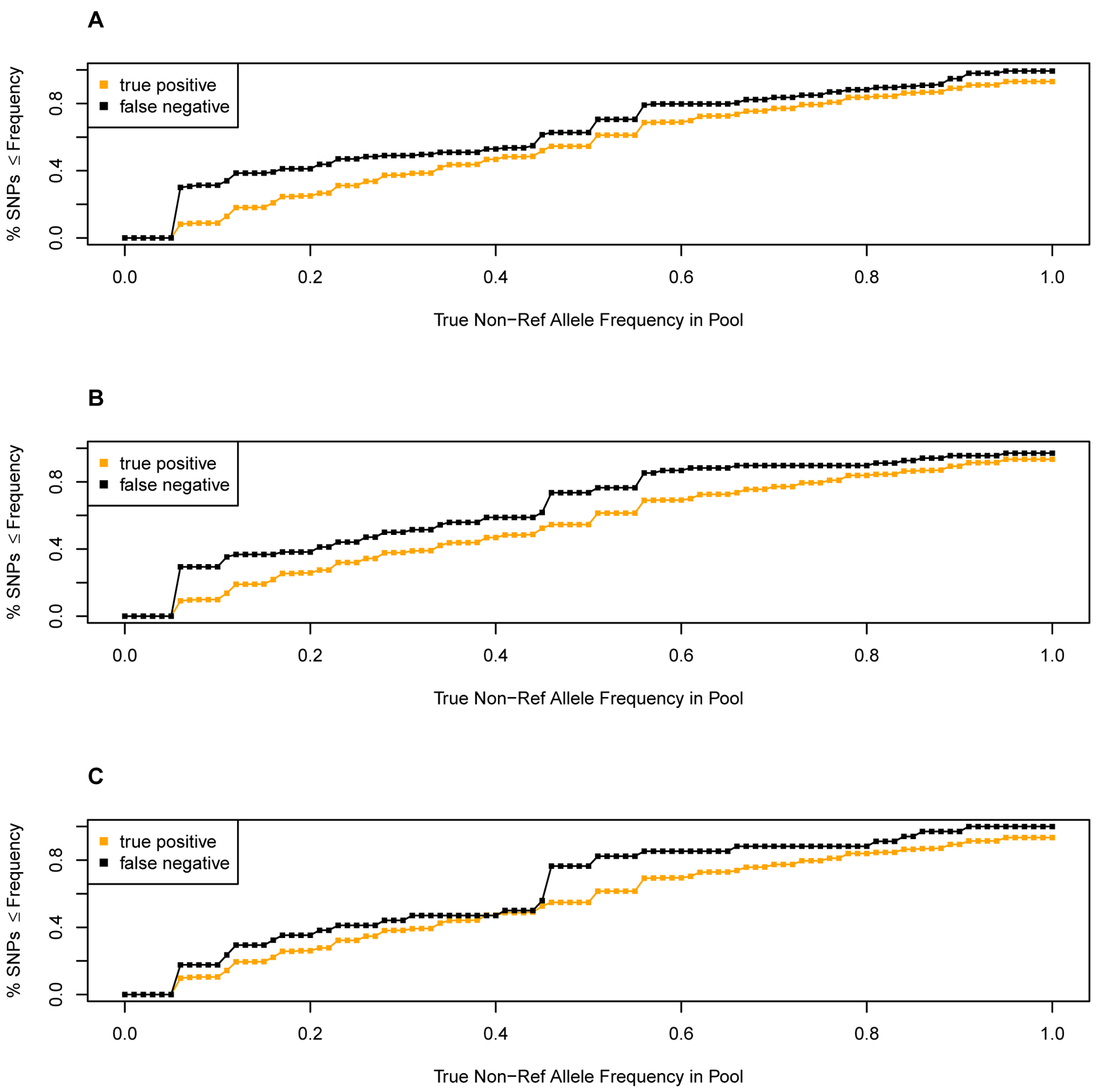

Supplement: Figure S21 — HapMap frequency distribution of true positive and false negative variants in the Pool of 10 after duplicate removal. This figure shows a cumulative relative frequency plot of the percentage of variants with true allele frequency X in the pool at HapMap true positive and false negative variants for: (A) PCR, (B) aHC and (C) sHC enrichment. The black squares/lines illustrate the data for false negative variants and the orange squares/lines illustrate the data for true positive variants. The first square represents the percentage of variants in a class with allele frequency 0.01, and so on for each square in 0.01 frequency increments. (TIF) [file pone.0026279.s021.tif]

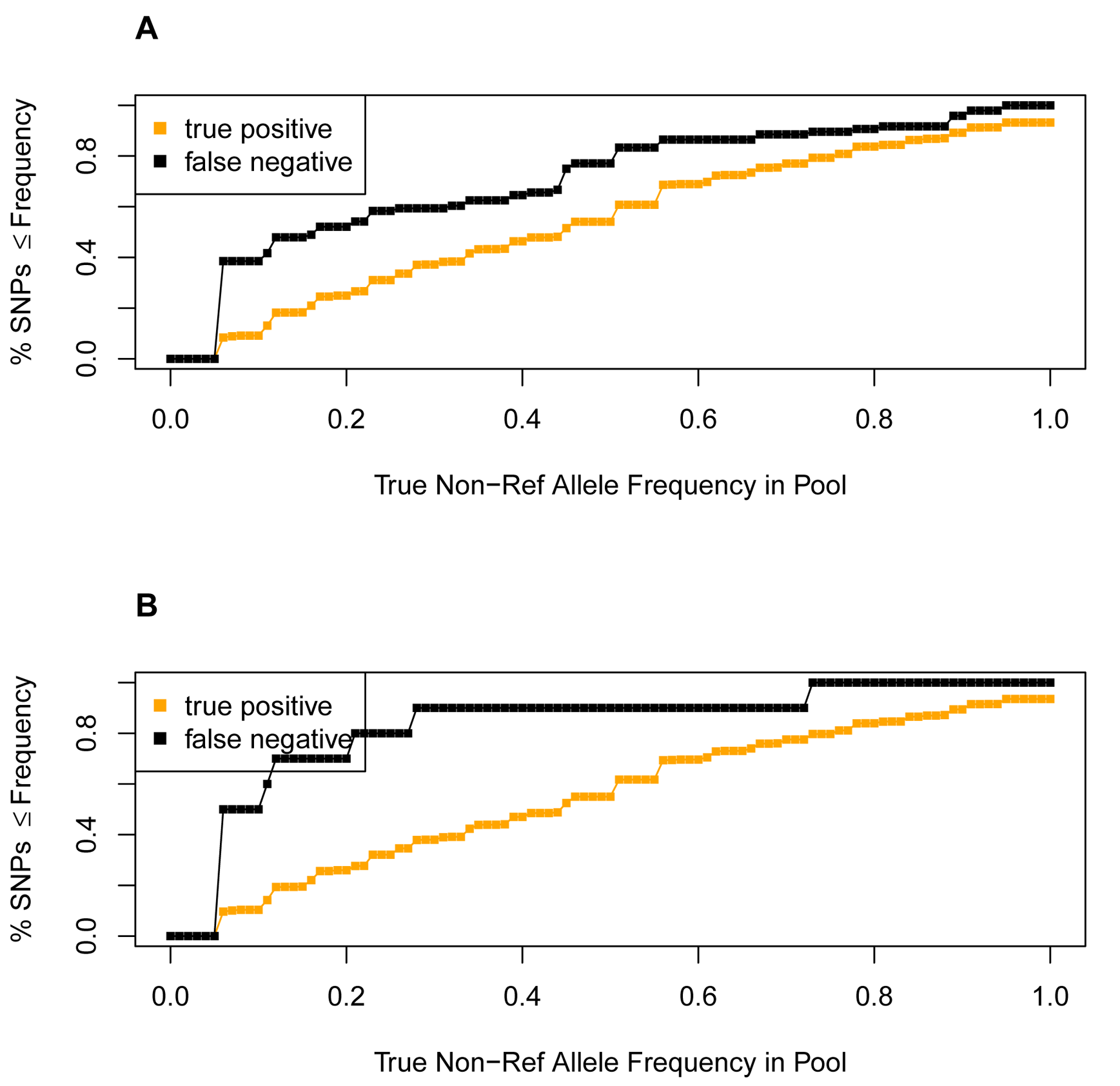

Supplement: Figure S22 — HapMap frequency distribution of true positive and false negative variants in the Pool of 10 before duplicate removal. This figure shows a cumulative relative frequency plot of the percentage of variants with true allele frequency X in the pool at HapMap true positive and false negative variants for: (A) PCR and (B) sHC enrichment. The black squares/lines illustrate the data for false negative variants and the orange squares/lines illustrate the data for true positive variants. The first square represents the percentage of variants in a class with allele frequency 0.01, and so on for each square in 0.01 frequency increments. (TIF) [file pone.0026279.s022.tif]

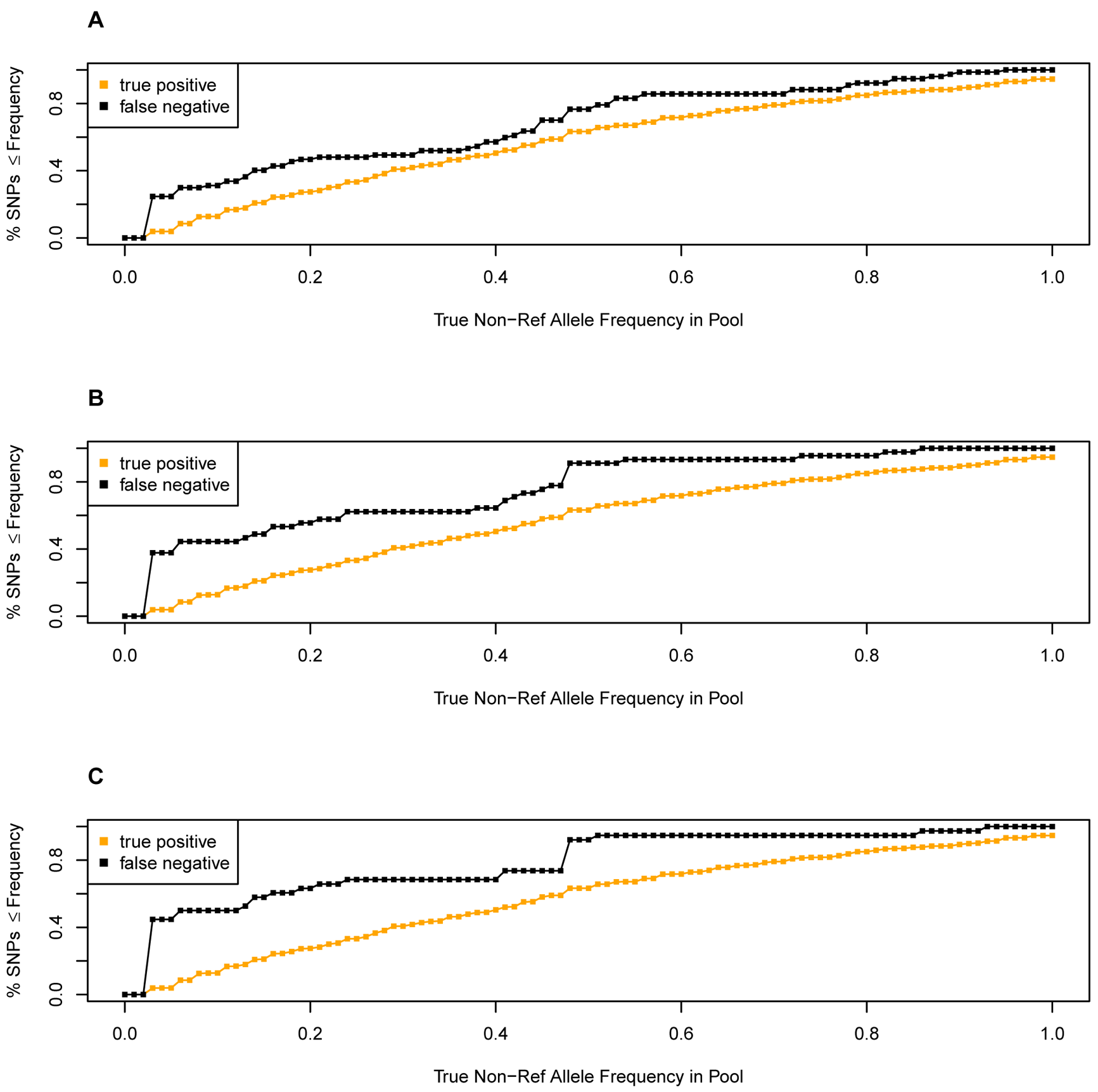

Supplement: Figure S23 — HapMap frequency distribution of true positive and false negative variants in the Pool of 20 after duplicate removal. This figure shows a cumulative relative frequency plot of the percentage of variants with true allele frequency X in the pool at HapMap true positive and false negative variants for: (A) PCR, (B) aHC and (C) sHC enrichment. The black squares/lines illustrate the data for false negative variants and the orange squares/lines illustrate the data for true positive variants. The first square represents the percentage of variants in a class with allele frequency 0.01, and so on for each square in 0.01 frequency increments. (TIF) [file pone.0026279.s023.tif]

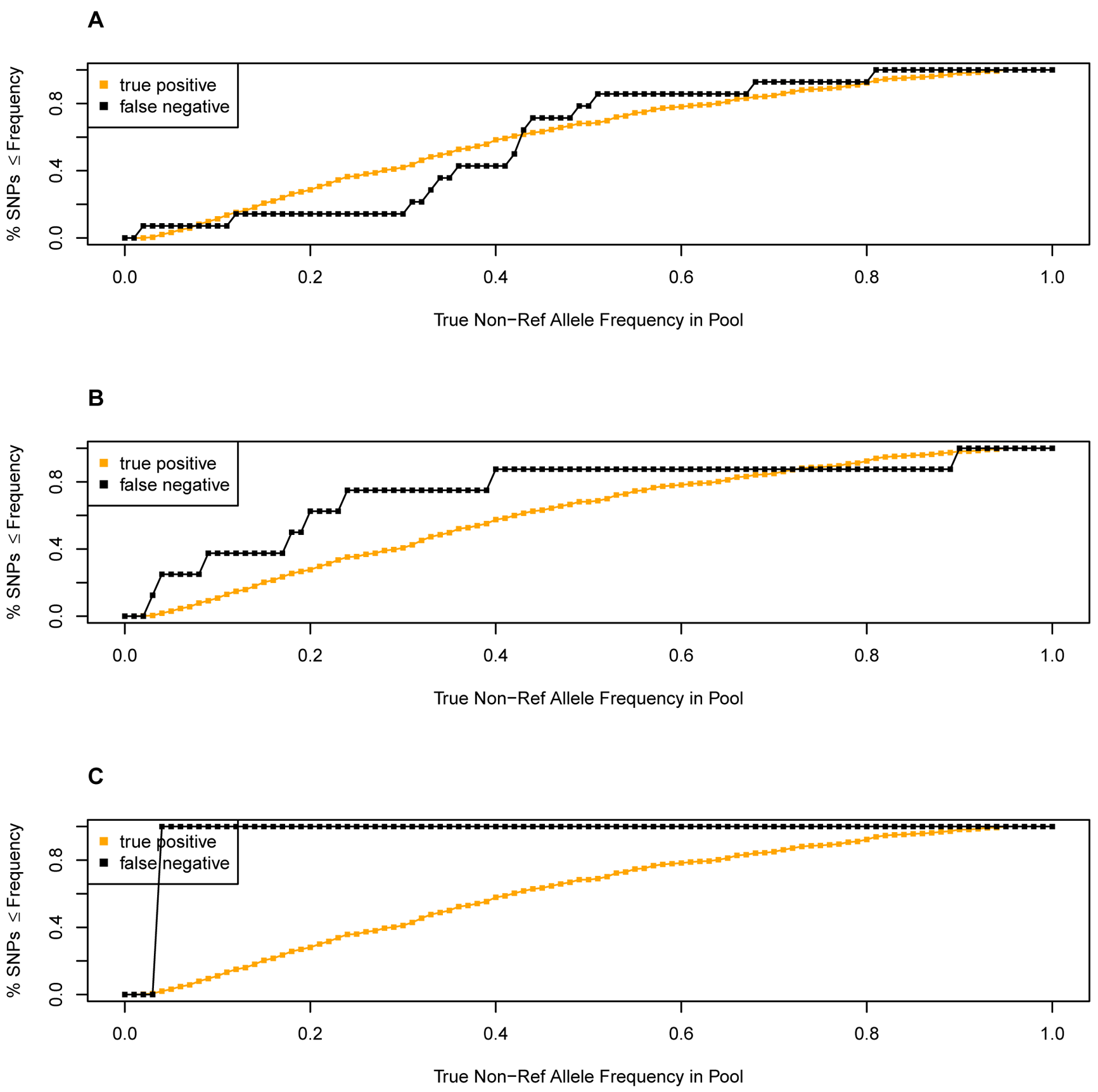

Supplement: Figure S24 — HapMap/58C intersection frequency distribution of true positive and false negative variants in the Pool of 50 after duplicate removal. This figure shows a cumulative relative frequency plot of the percentage of variants with true allele frequency X in the pool at HapMap/58C intersection true positive and false negative variants for: (A) PCR, (B) aHC and (C) sHC enrichment. The black squares/lines illustrate the data for false negative variants and the orange squares/lines illustrate the data for true positive variants. The first square represents the percentage of variants in a class with allele frequency 0.01, and so on for each square in 0.01 frequency increments. This analysis is for the 507 sites where all 50 individuals had genotype data for, which lead to only 14 false negatives for PCR, 8 false negatives for aHC and 1 false negative for sHC. (TIF) [file pone.0026279.s024.tif]

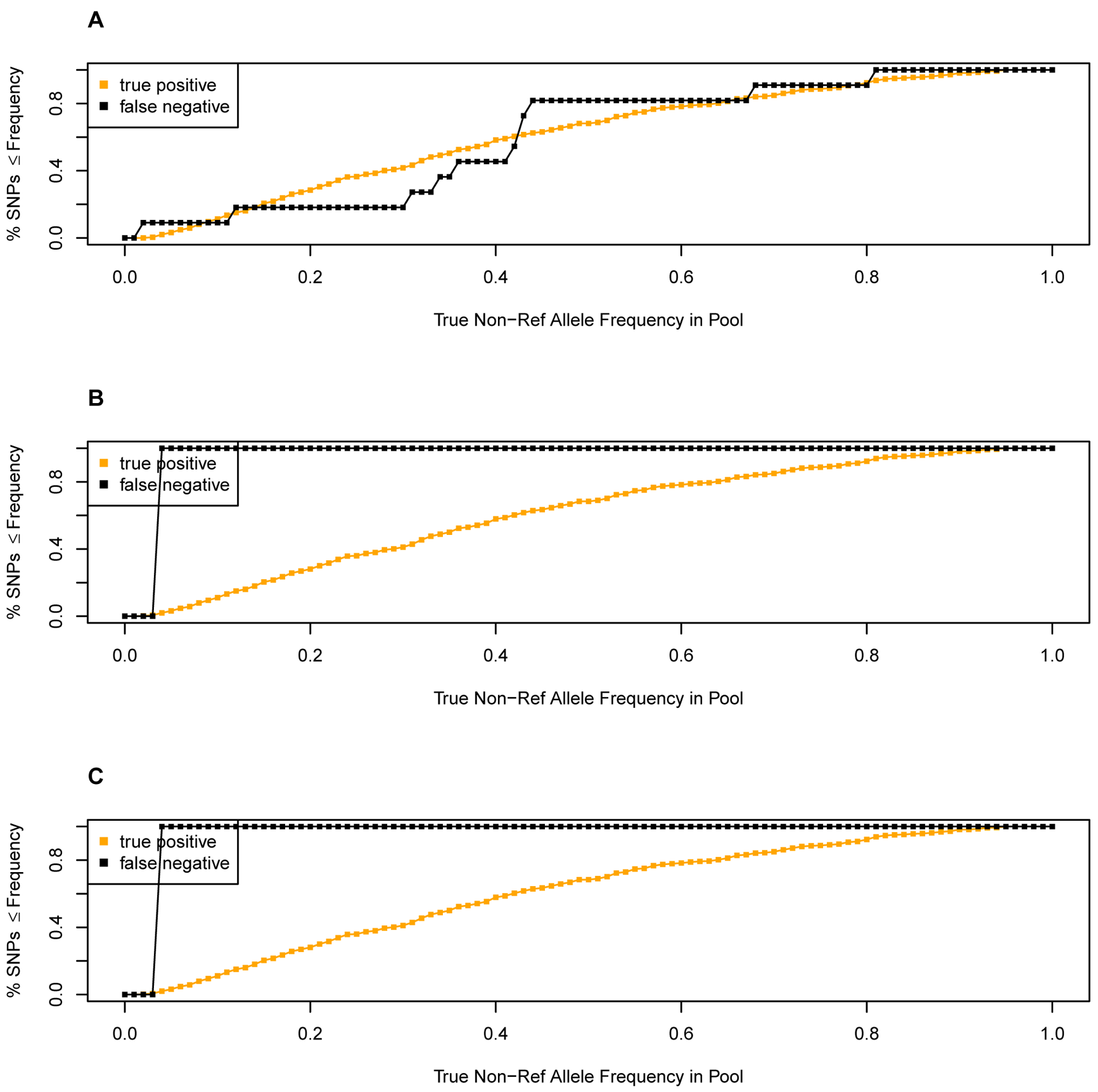

Supplement: Figure S25 — HapMap/58C intersection frequency distribution of true positive and false negative variants in the Pool of 50 before duplicate removal. This figure shows a cumulative relative frequency plot of the percentage of variants with true allele frequency X in the pool at HapMap/58C intersection true positive and false negative variants for: (A) PCR, (B) aHC and (C) sHC enrichment. The black squares/lines illustrate the data for false negative variants and the orange squares/lines illustrate the data for true positive variants. The first square represents the percentage of variants in a class with allele frequency 0.01, and so on for each square in 0.01 frequency increments. This analysis is for the 507 sites where all 50 individuals had genotype data for, which lead to only 11 false negatives for PCR, 1 false negatives for aHC and 1 false negative for sHC. (TIF) [file pone.0026279.s025.tif]

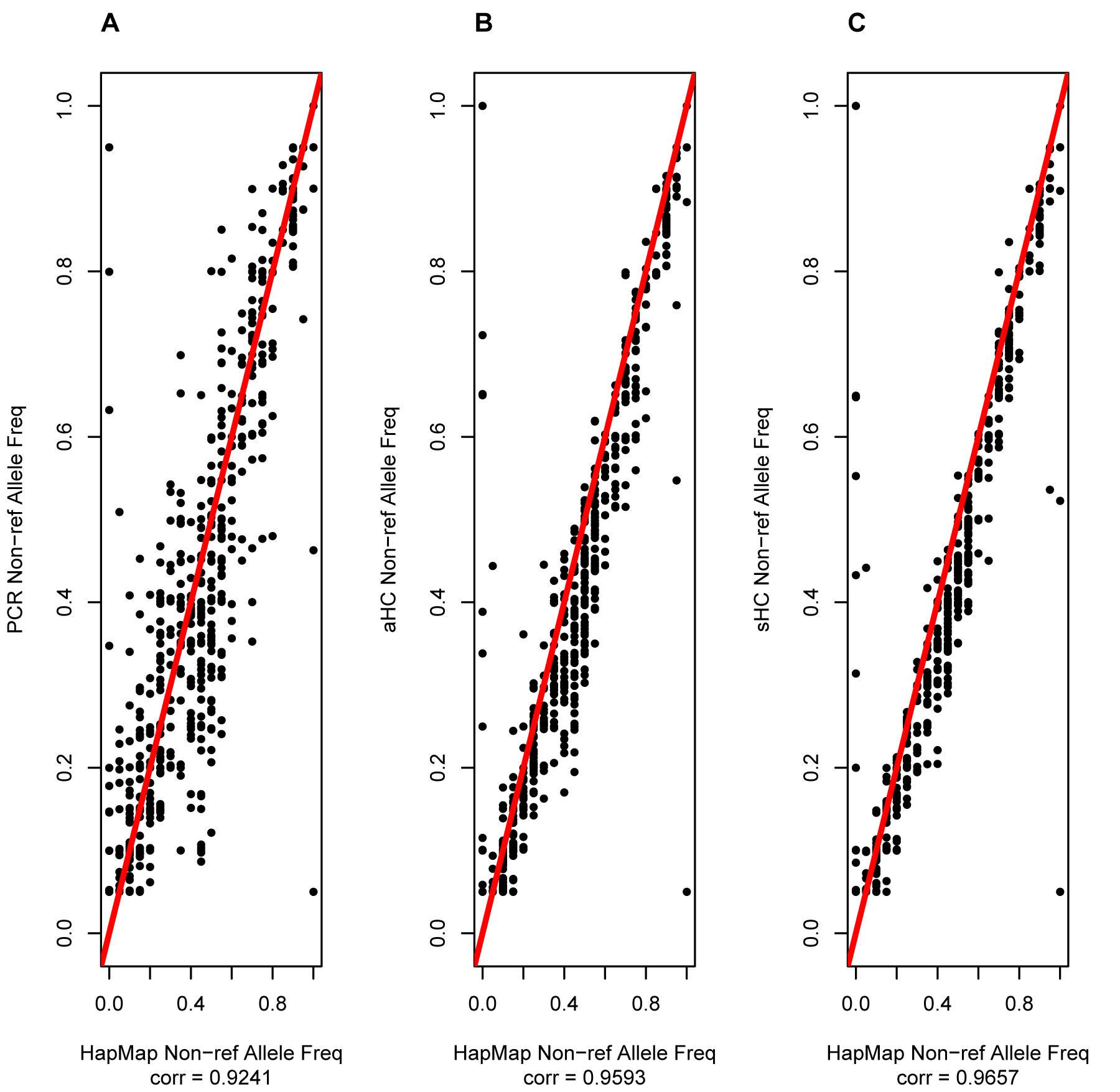

Supplement: Figure S26 — Accuracy of non-reference allele frequency estimation at HapMap variants for the Pool of 10 after duplicate removal. An analysis of the correlation between the non-reference allele frequency estimate from the sequencing based variant caller and the allele frequency from the reference genotypes. The analysis includes the true positive variants called by the sequencing based variant caller for which there were no missing genotypes in the reference genotypes. The correlation coefficient is the Pearson's correlation coefficient. The figure shows the analysis for: (A) PCR, (B) aHC and (C) sHC enrichment. (TIF) [file pone.0026279.s026.tif]

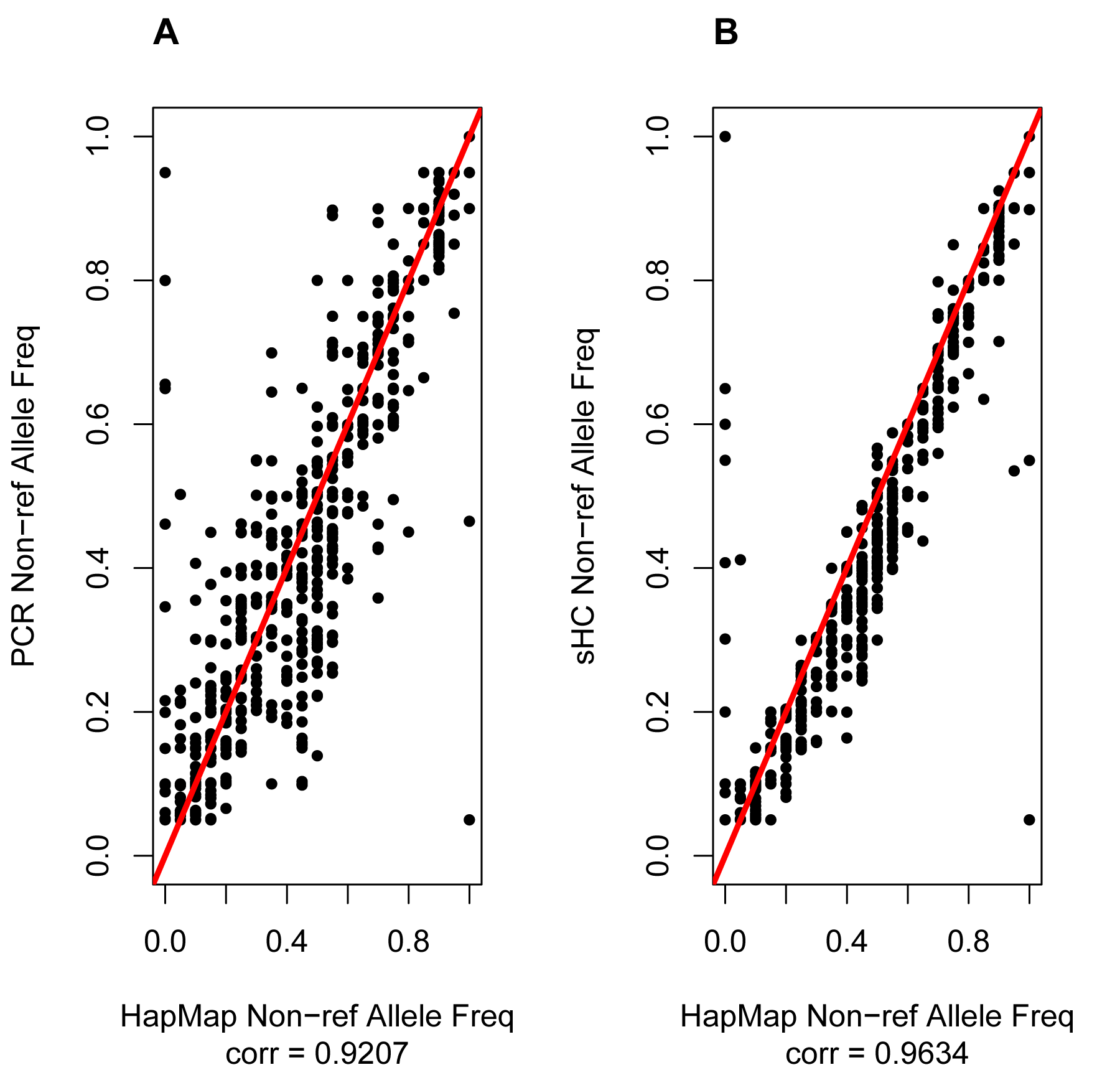

Supplement: Figure S27 — Accuracy of non-reference allele frequency estimation at HapMap variants for the Pool of 10 before duplicate removal. An analysis of the correlation between the non-reference allele frequency estimate from the sequencing based variant caller and the allele frequency from the reference genotypes. The analysis includes the true positive variants called by the sequencing based variant caller for which there were no missing genotypes in the reference genotypes. The correlation coefficient is the Pearson's correlation coefficient. The figure shows the analysis for: (A) PCR and (B) sHC enrichment. (TIF) [file pone.0026279.s027.tif]

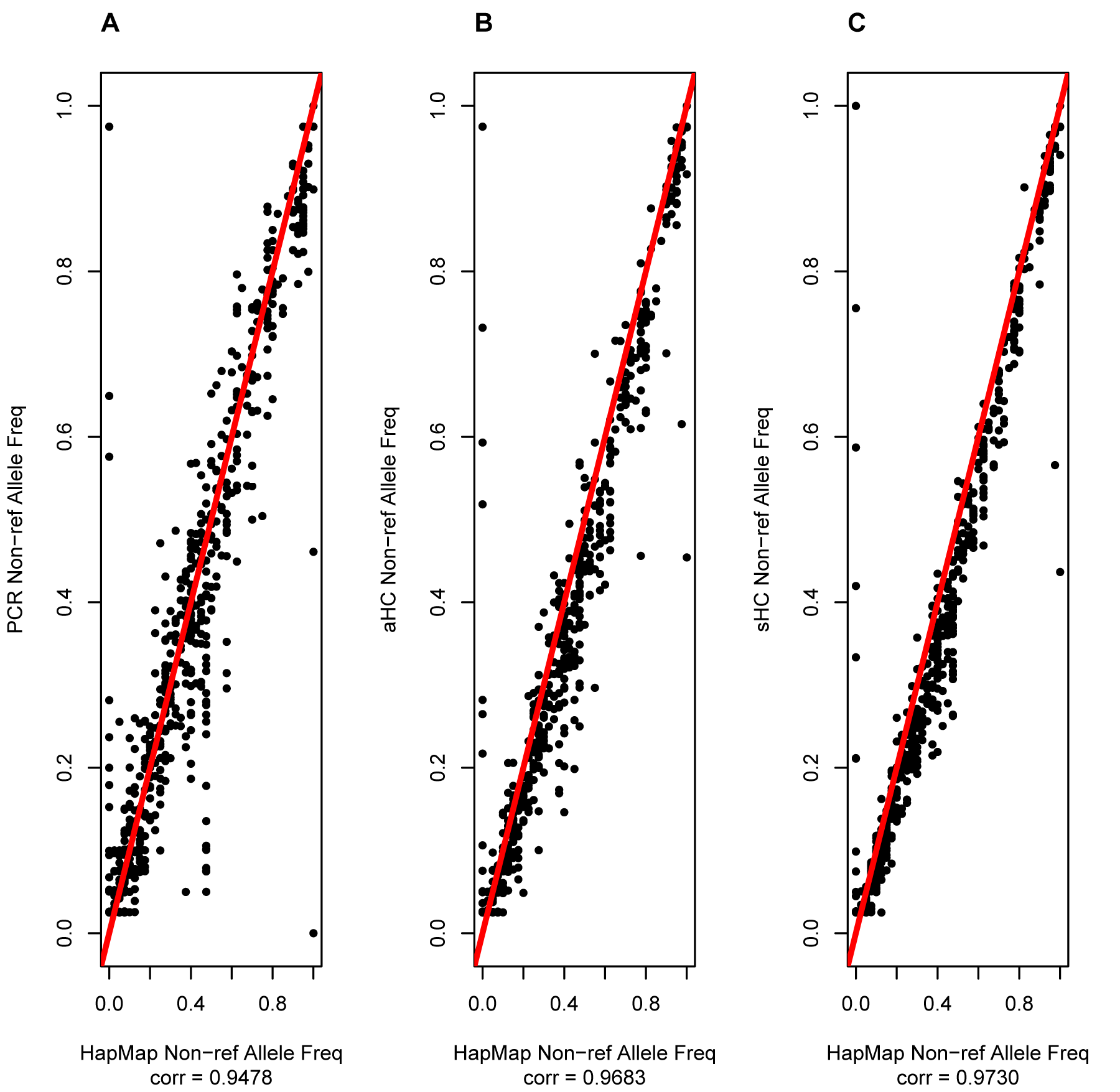

Supplement: Figure S28 — Accuracy of non-reference allele frequency estimation at HapMap variants for the Pool of 20 after duplicate removal. An analysis of the correlation between the non-reference allele frequency estimate from the sequencing based variant caller and the allele frequency from the reference genotypes. The analysis includes the true positive variants called by the sequencing based variant caller for which there were no missing genotypes in the reference genotypes. The correlation coefficient is the Pearson's correlation coefficient. The figure shows the analysis for: (A) PCR, (B) aHC and (C) sHC enrichment. (TIF) [file pone.0026279.s028.tif]

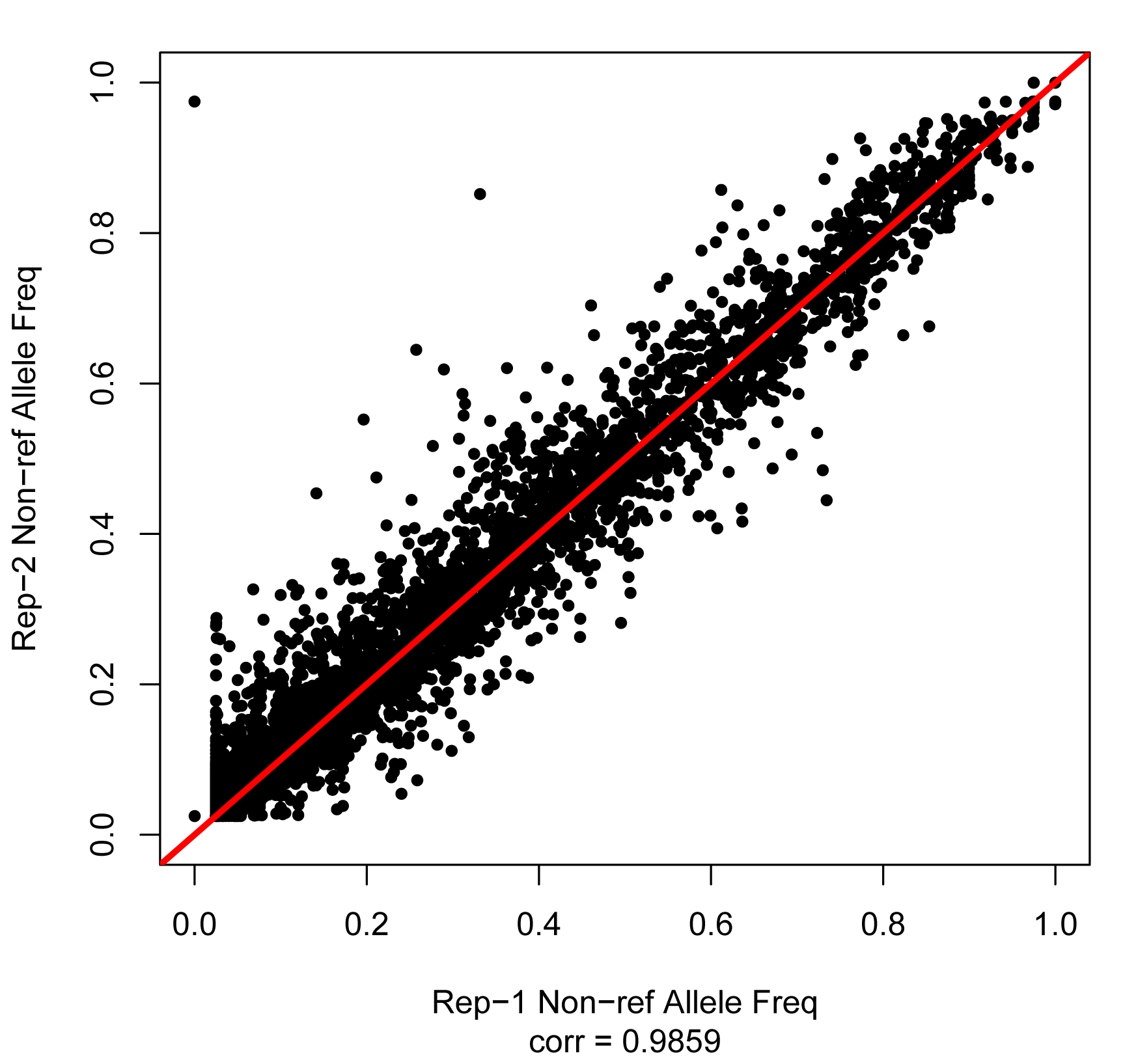

Supplement: Figure S29 — Comparison of non-reference allele frequency estimation for Pool of 20 PCR technical replicates after duplicate removal. The correlation of non-reference allele frequency estimates for overlapping variants between the PCR technical replicates. The y-axis are the non-reference allele frequencies for replicate 2 and the x-axis are the non-reference allele frequencies for replicate 1. The correlation is the Pearson' correlation coefficient between allele frequencies. (TIF) [file pone.0026279.s029.tif]

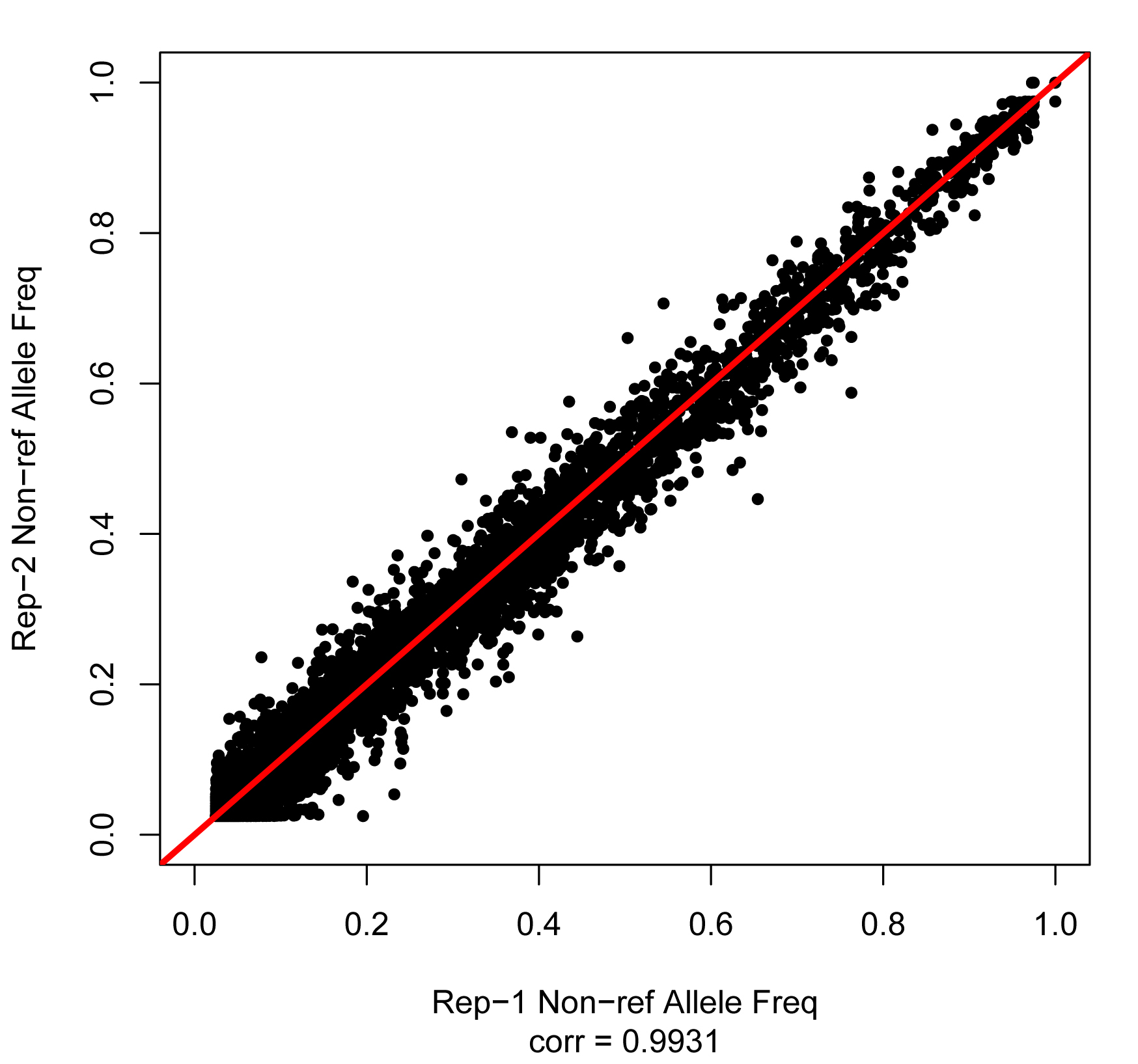

Supplement: Figure S30 — Comparison of non-reference allele frequency estimation for Pool of 20 aHC technical replicates after duplicate removal. The correlation of non-reference allele frequency estimates for overlapping variants between the aHC technical replicates. The y-axis are the non-reference allele frequencies for replicate 2 and the x-axis are the non-reference allele frequencies for replicate 1. The correlation is the Pearson' correlation coefficient between allele frequencies. (TIF) [file pone.0026279.s030.tif]

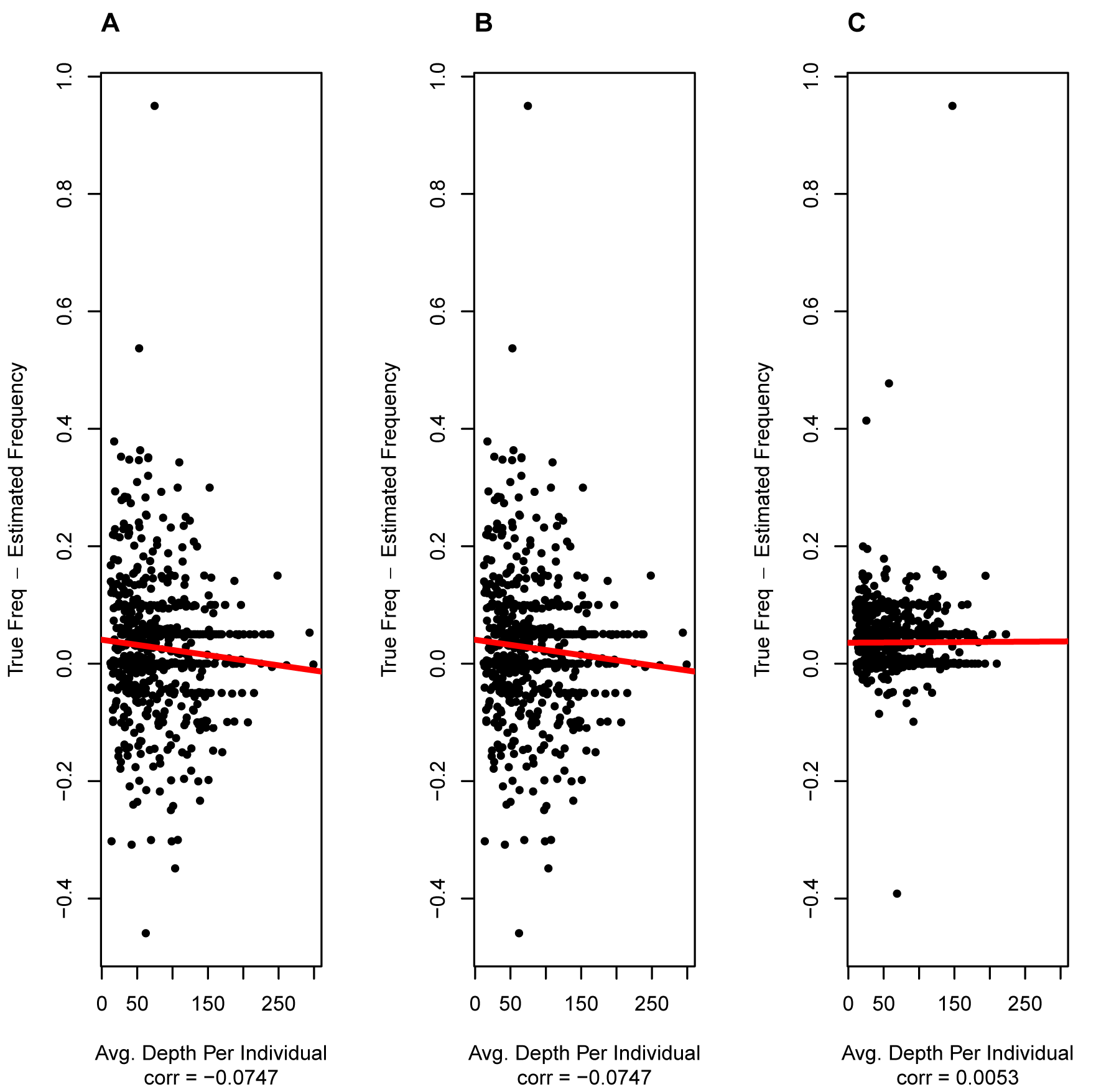

Supplement: Figure S31 — HapMap allele frequency estimation accuracy as a function of per individual depth in the Pool of 10 after duplicate removal. This figure is a scatter plot of the accuracy of the allele frequency estimates from the sequencing compared to the per individual read depth at HapMap true positive variants in the Pool of 10 individuals for: (A) PCR, (B) aHC and (C) sHC enrichment. The accuracy of the estimates are calculated as the frequency calculated from the HapMap genotypes minus the frequency estimated from the sequencing data. The y-axis is the accuracy value and the x-axis is the per individual read depth in the pool. The red line is the least squares fit of the model , and the corr is the Pearson's correlation coefficient between the accuracy and read depth. (TIF) [file pone.0026279.s031.tif]

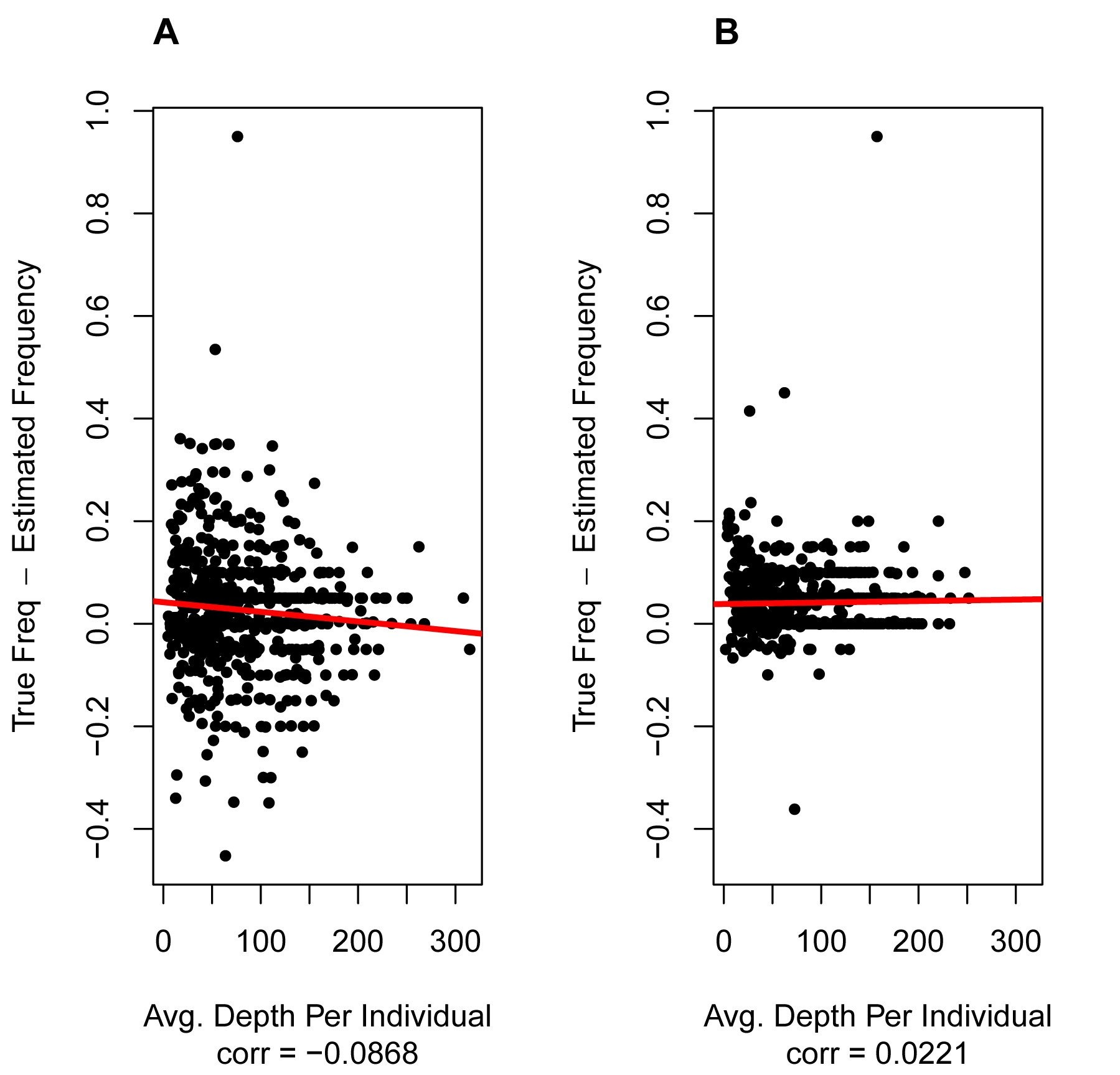

Supplement: Figure S32 — HapMap allele frequency estimation accuracy as a function of per individual depth in the Pool of 10 before duplicate removal. This figure is a scatter plot of the accuracy of the allele frequency estimates from the sequencing compared to the per individual read depth at HapMap true positive variants in the Pool of 10 individuals for: (A) PCR and (B) sHC enrichment. The accuracy of the estimates are calculated as the frequency calculated from the HapMap genotypes minus the frequency estimated from the sequencing data. The y-axis is the accuracy value and the x-axis is the per individual read depth in the pool. The red line is the least squares fit of the model , and the corr is the Pearson's correlation coefficient between the accuracy and read depth. (TIF) [file pone.0026279.s032.tif]

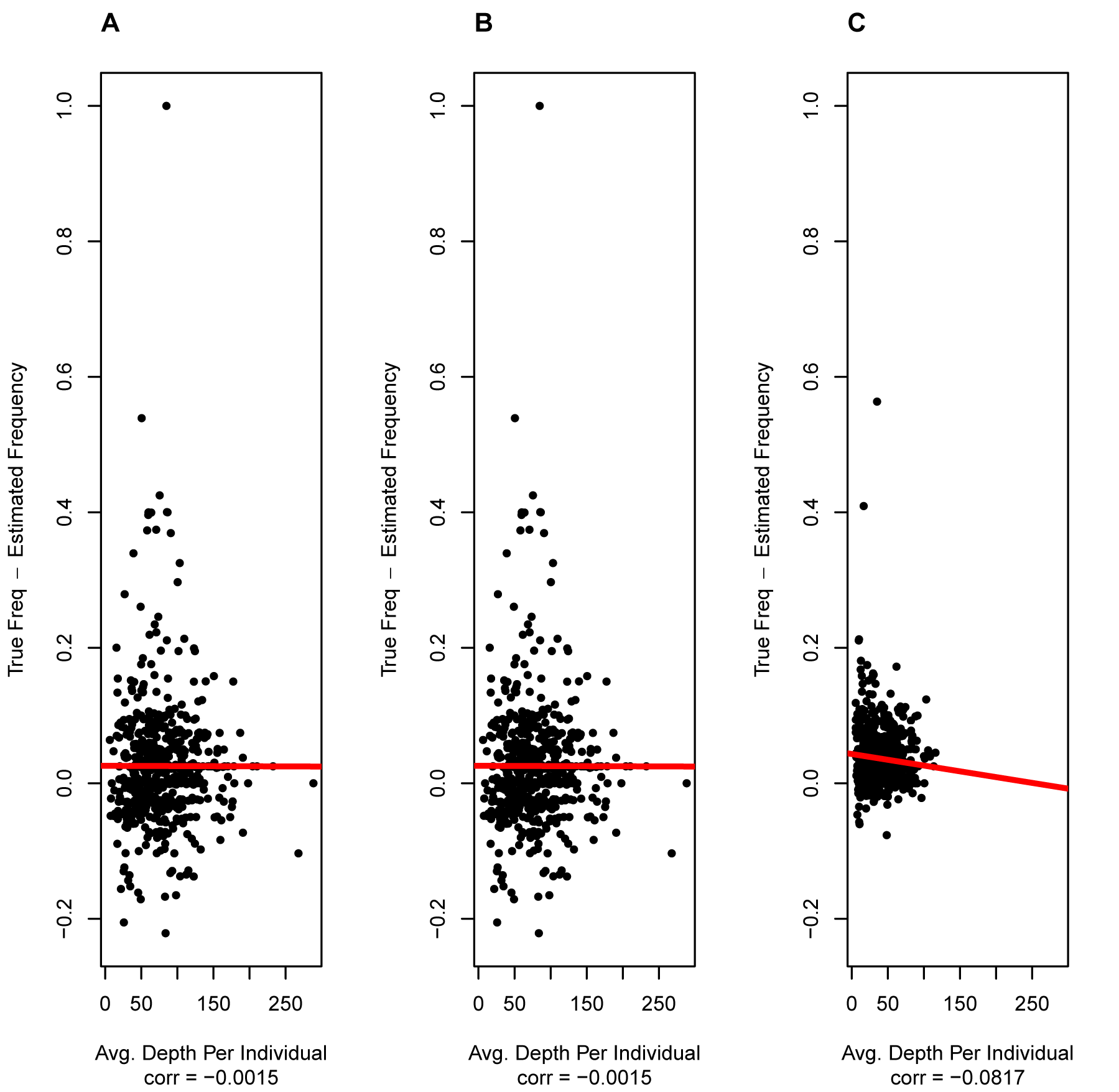

Supplement: Figure S33 — HapMap allele frequency estimation accuracy as a function of per individual depth in the Pool of 20 after duplicate removal. This figure is a scatter plot of the accuracy of the allele frequency estimates from the sequencing compared to the per individual read depth at HapMap true positive variants in the Pool of 20 individuals for: (A) PCR, (B) aHC and (C) sHC enrichment. The accuracy of the estimates are calculated as the frequency calculated from the HapMap genotypes minus the frequency estimated from the sequencing data. The y-axis is the accuracy value and the x-axis is the per individual read depth in the pool. The red line is the least squares fit of the model , and the corr is the Pearson's correlation coefficient between the accuracy and read depth. (TIF) [file pone.0026279.s033.tif]

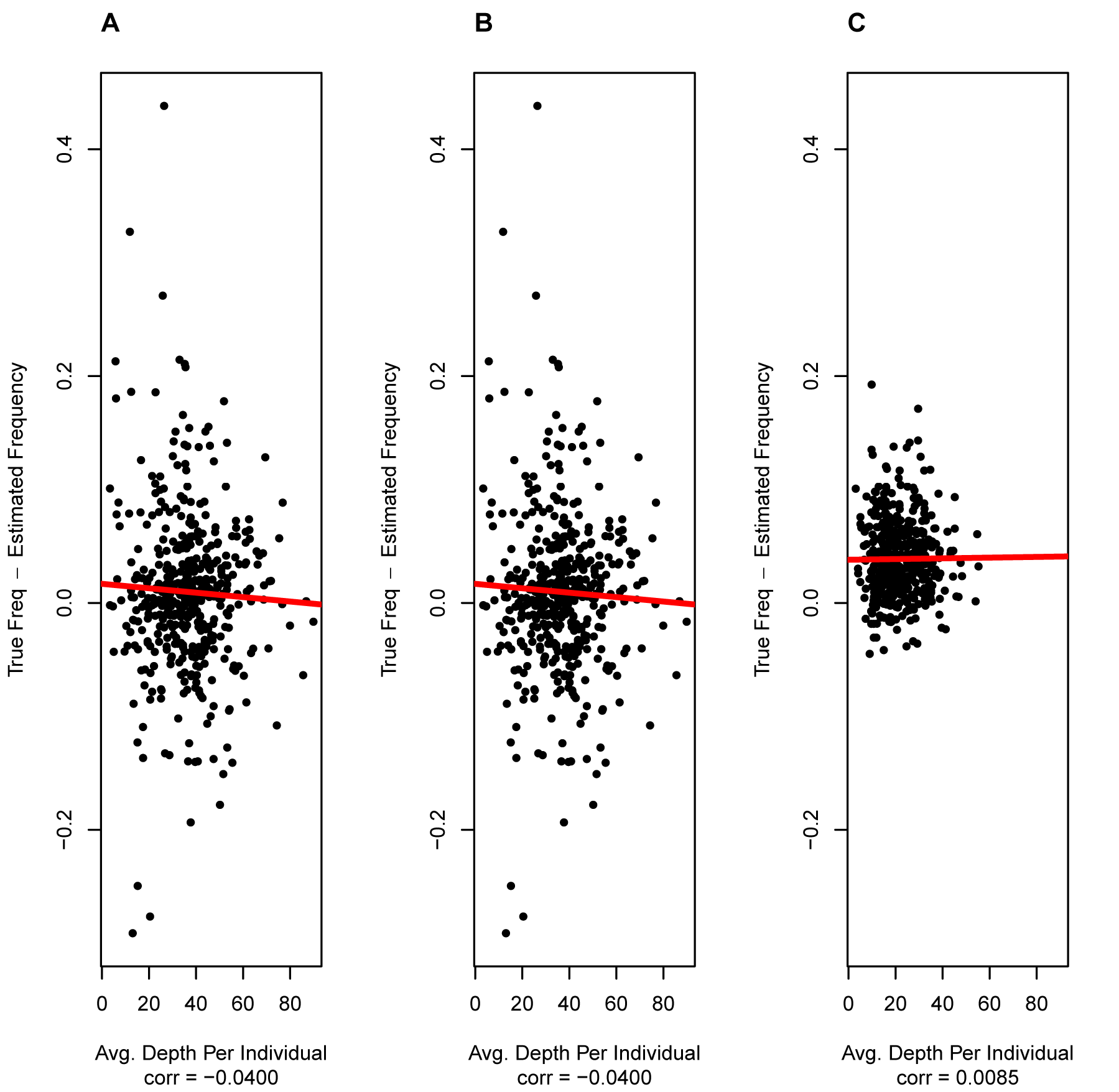

Supplement: Figure S34 — HapMap/58BC intersection allele frequency estimation accuracy as a function of per individual depth in the Pool of 50 after duplicate removal. This figure is a scatter plot of the accuracy of the allele frequency estimates from the sequencing compared to the per individual read depth at HapMap/58BC intersection true positive variants in the Pool of 50 individuals for: (A) PCR, (B) aHC and (C) sHC enrichment. The accuracy of the estimates are calculated as the frequency calculated from the HapMap genotypes minus the frequency estimated from the sequencing data. The y-axis is the accuracy value and the x-axis is the per individual read depth in the pool. The red line is the least squares fit of the model , and the corr is the Pearson's correlation coefficient between the accuracy and read depth. (TIF) [file pone.0026279.s034.tif]

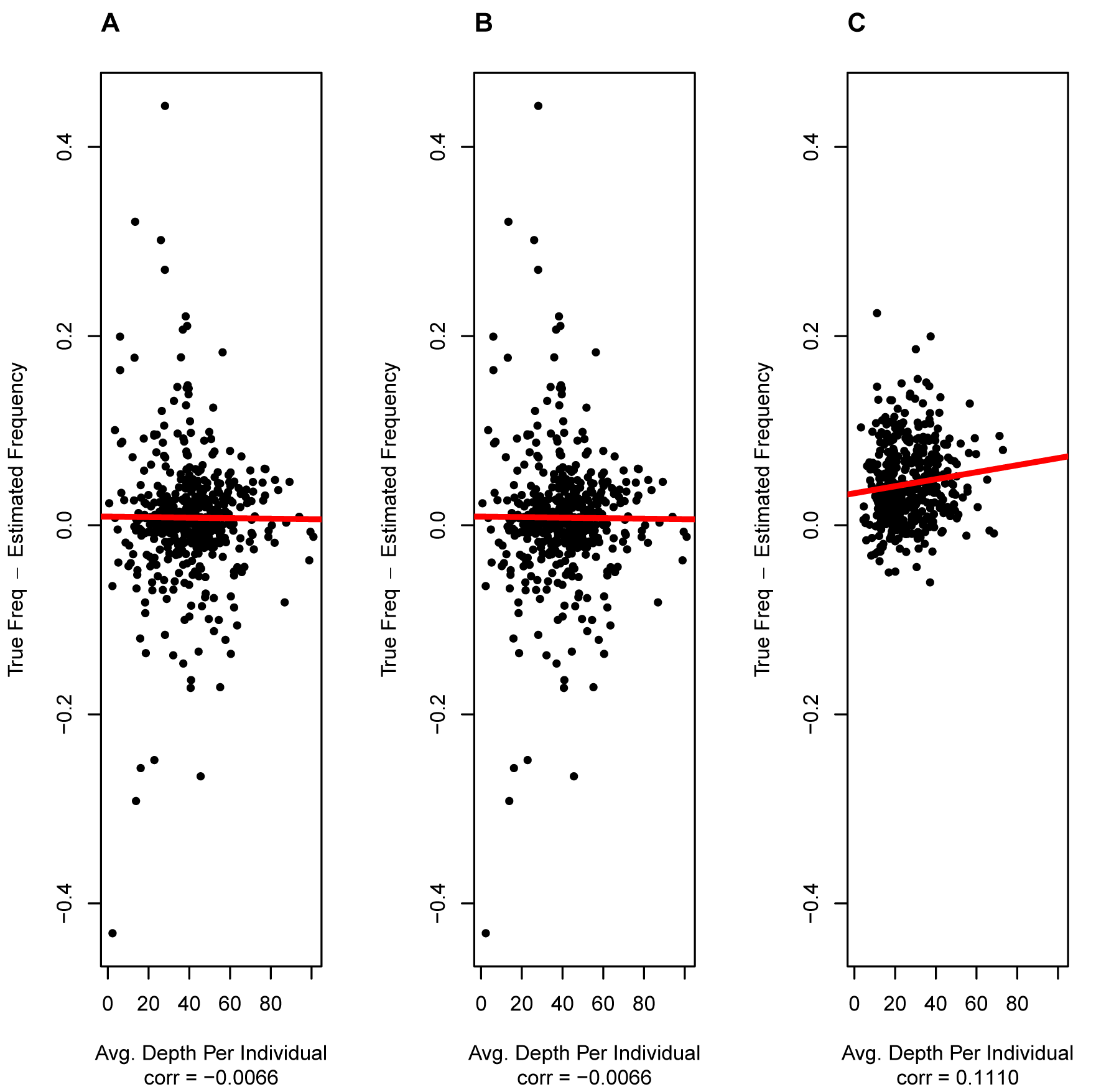

Supplement: Figure S35 — HapMap/58BC intersection allele frequency estimation accuracy as a function of per individual depth in the Pool of 50 before duplicate removal. This figure is a scatter plot of the accuracy of the allele frequency estimates from the sequencing compared to the per individual read depth at HapMap/58BC intersection true positive variants in the Pool of 50 individuals for: (A) PCR, (B) aHC and (C) sHC enrichment. The accuracy of the estimates are calculated as the frequency calculated from the HapMap genotypes minus the frequency estimated from the sequencing data. The y-axis is the accuracy value and the x-axis is the per individual read depth in the pool. The red line is the least squares fit of the model , and the corr is the Pearson's correlation coefficient between the accuracy and read depth. (TIF) [file pone.0026279.s035.tif]

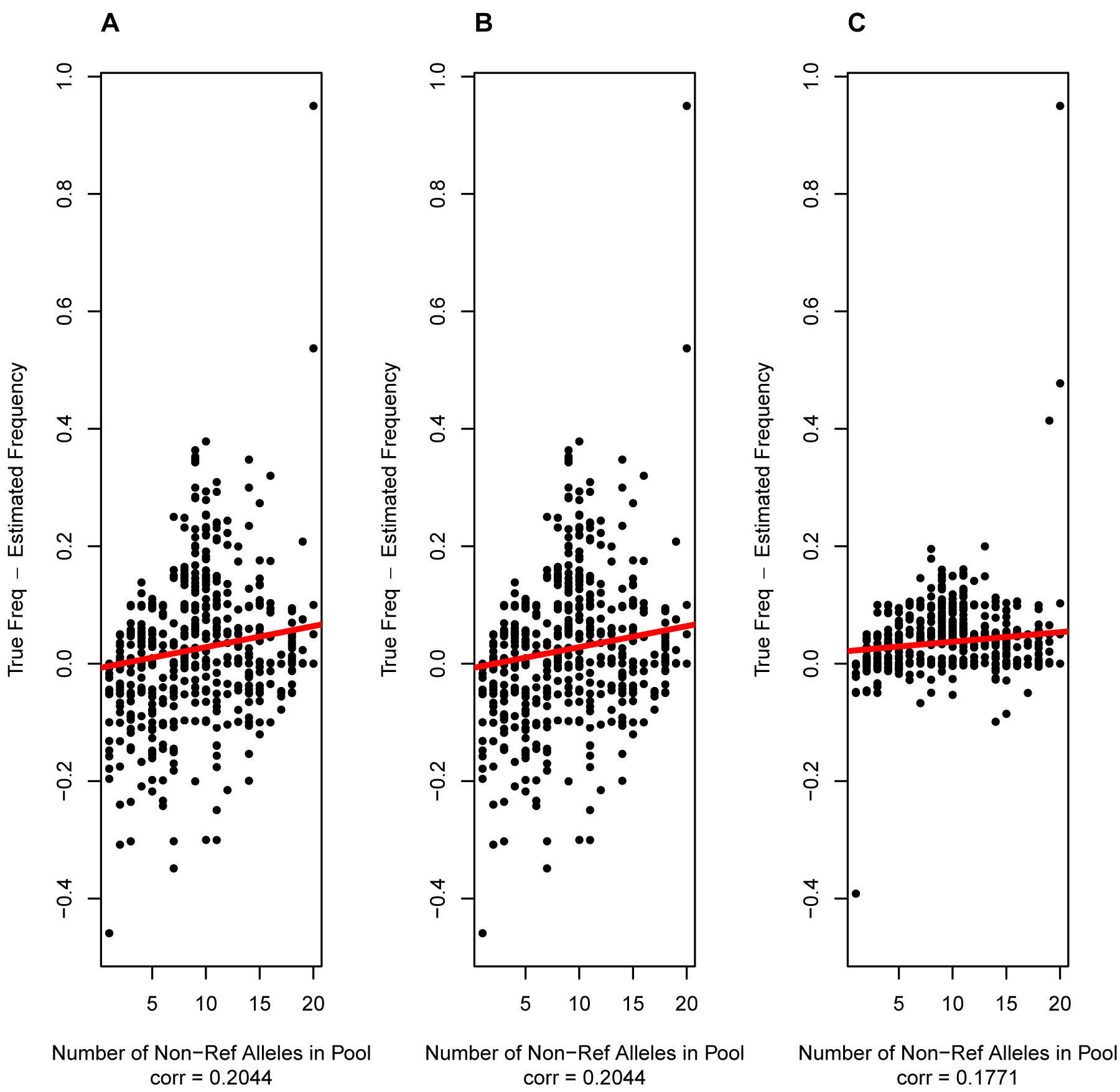

Supplement: Figure S36 — HapMap allele frequency estimation accuracy as a function of allele count in the Pool of 10 after duplicate removal. This figure is a scatter plot of the accuracy of the allele frequency estimates from the sequencing compared to the number of variant alleles at HapMap true positive variants in the Pool of 10 individuals for: (A) PCR, (B) aHC and (C) sHC enrichment. The accuracy of the estimates are calculated as the frequency calculated from the HapMap genotypes minus the frequency estimated from the sequencing data. The y-axis is the accuracy value and the x-axis is the number of variant alleles in the pool. The red line is the least squares fit of the model , and the corr is the Pearson's correlation coefficient between the accuracy and read depth. (TIF) [file pone.0026279.s036.tif]

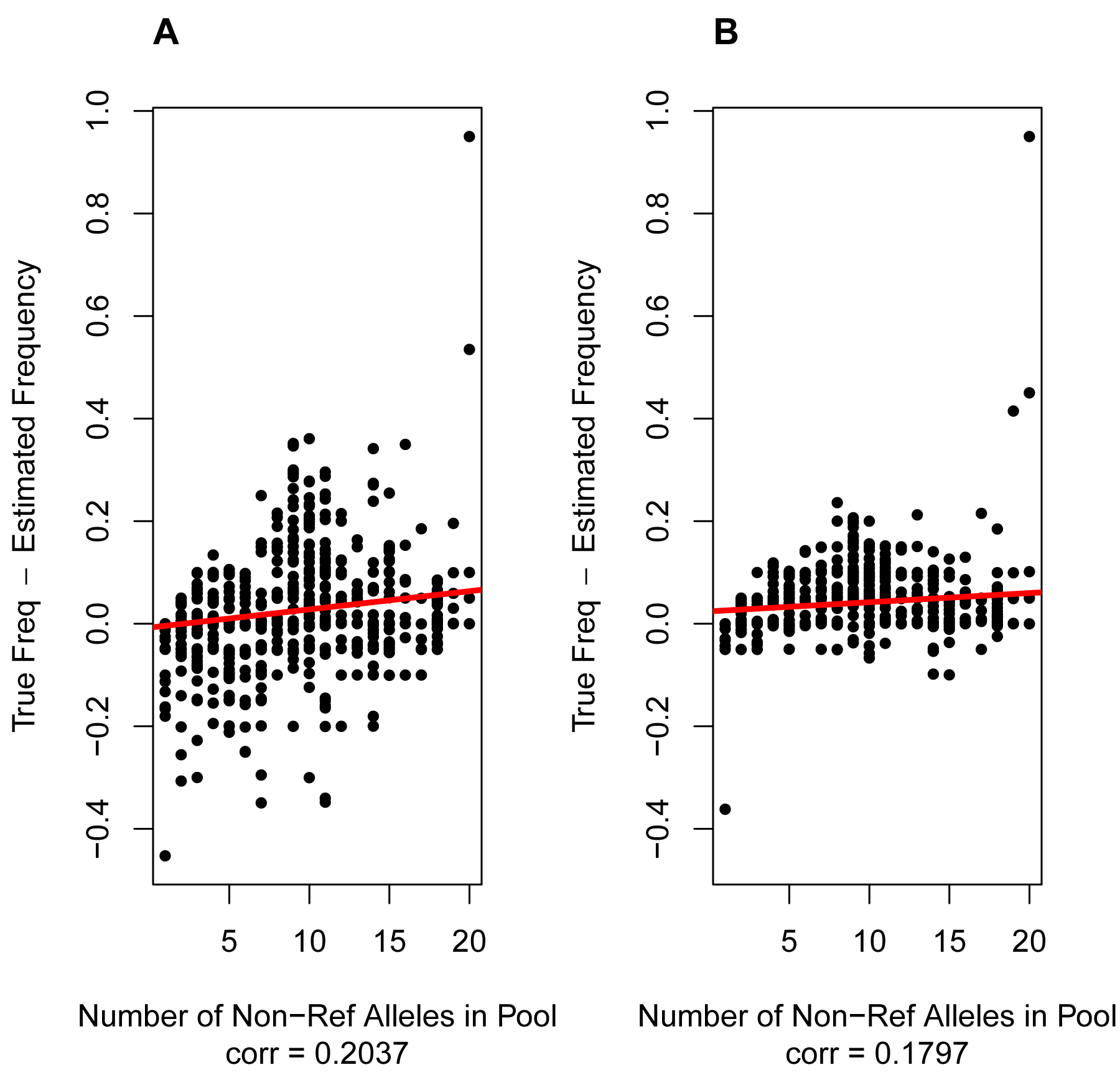

Supplement: Figure S37 — HapMap allele frequency estimation accuracy as a function of allele count in the Pool of 10 before duplicate removal. This figure is a scatter plot of the accuracy of the allele frequency estimates from the sequencing compared to the number of variant alleles at HapMap true positive variants in the Pool of 10 individuals for: (A) PCR and (B) sHC enrichment. The accuracy of the estimates are calculated as the frequency calculated from the HapMap genotypes minus the frequency estimated from the sequencing data. The y-axis is the accuracy value and the x-axis is the number of variant alleles in the pool. The red line is the least squares fit of the model , and the corr is the Pearson's correlation coefficient between the accuracy and read depth. (TIF) [file pone.0026279.s037.tif]

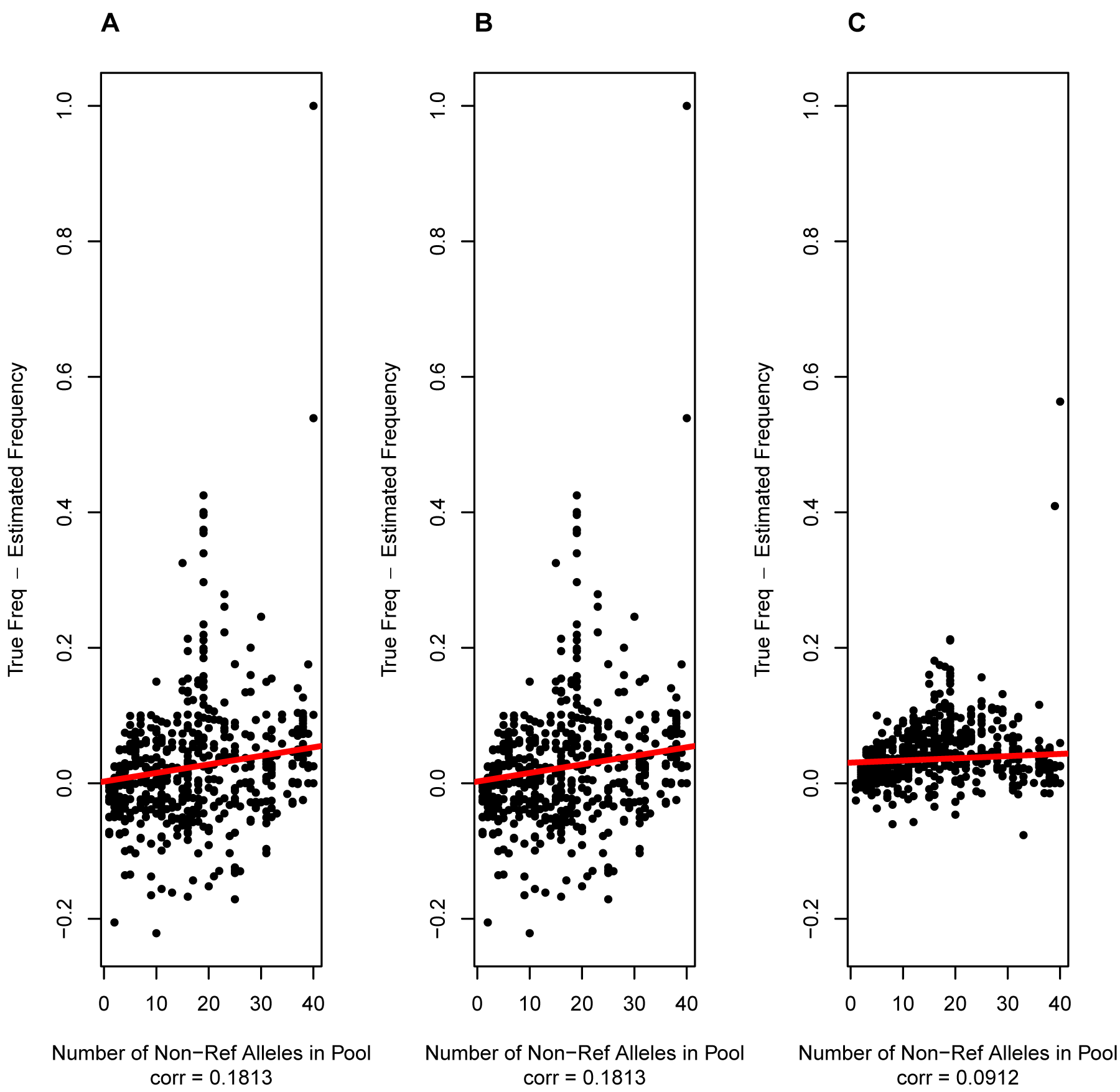

Supplement: Figure S38 — HapMap allele frequency estimation accuracy as a function of allele count in the Pool of 20 after duplicate removal. This figure is a scatter plot of the accuracy of the allele frequency estimates from the sequencing compared to the number of variant alleles at HapMap true positive variants in the Pool of 20 individuals for: (A) PCR, (B) aHC and (C) sHC enrichment. The accuracy of the estimates are calculated as the frequency calculated from the HapMap genotypes minus the frequency estimated from the sequencing data. The y-axis is the accuracy value and the x-axis is the number of variant alleles in the pool. The red line is the least squares fit of the model , and the corr is the Pearson's correlation coefficient between the accuracy and read depth. (TIF) [file pone.0026279.s038.tif]

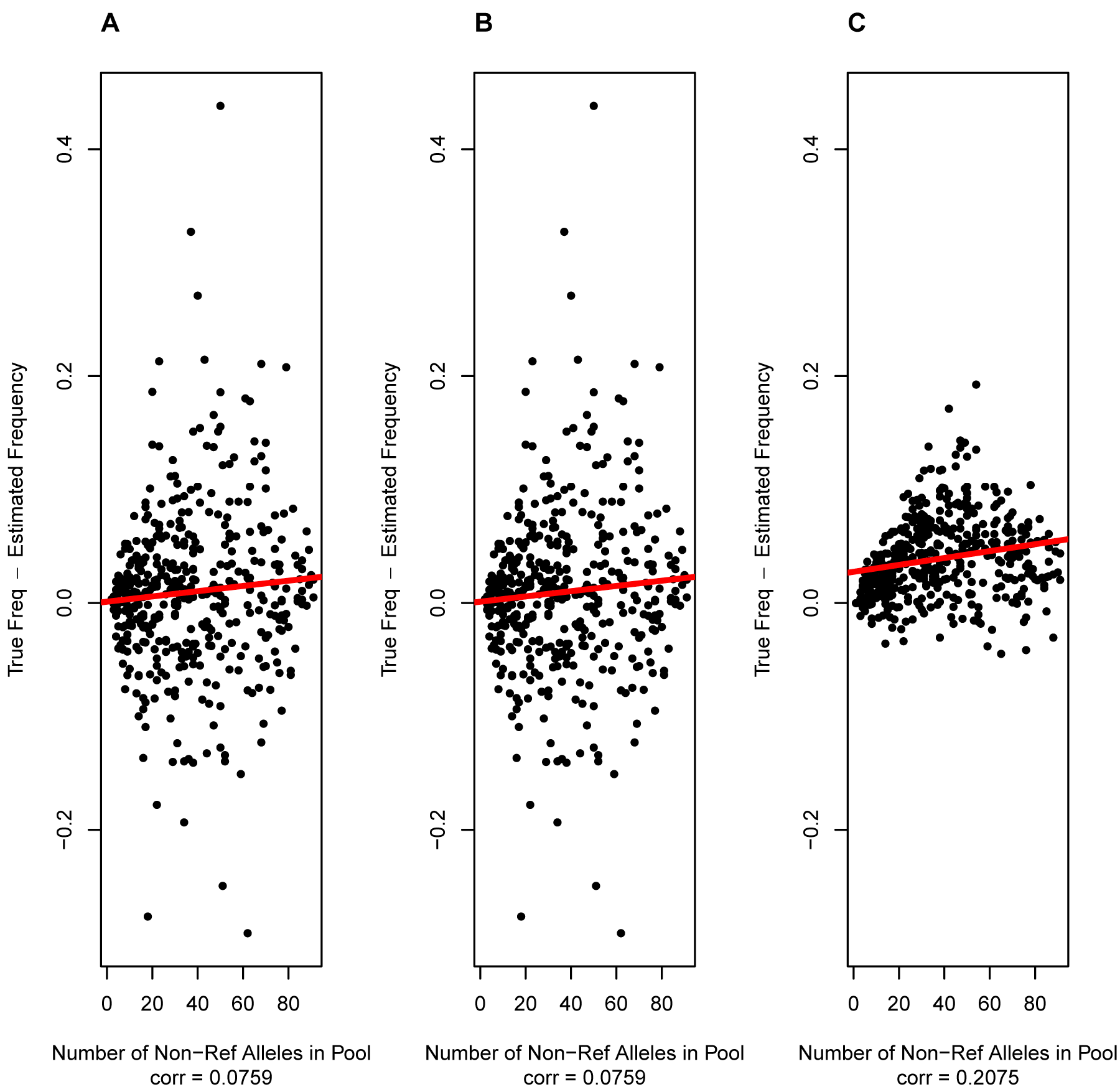

Supplement: Figure S39 — HapMap/58BC intersection allele frequency estimation accuracy as a function of allele count in the Pool of 50 after duplicate removal. This figure is a scatter plot of the accuracy of the allele frequency estimates from the sequencing compared to the number of variant alleles at HapMap/58BC intersection true positive variants in the Pool of 50 individuals for: (A) PCR, (B) aHC and (C) sHC enrichment. The accuracy of the estimates are calculated as the frequency calculated from the HapMap genotypes minus the frequency estimated from the sequencing data. The y-axis is the accuracy value and the x-axis is the number of variant alleles in the pool. The red line is the least squares fit of the model , and the corr is the Pearson's correlation coefficient between the accuracy and read depth. (TIF) [file pone.0026279.s039.tif]

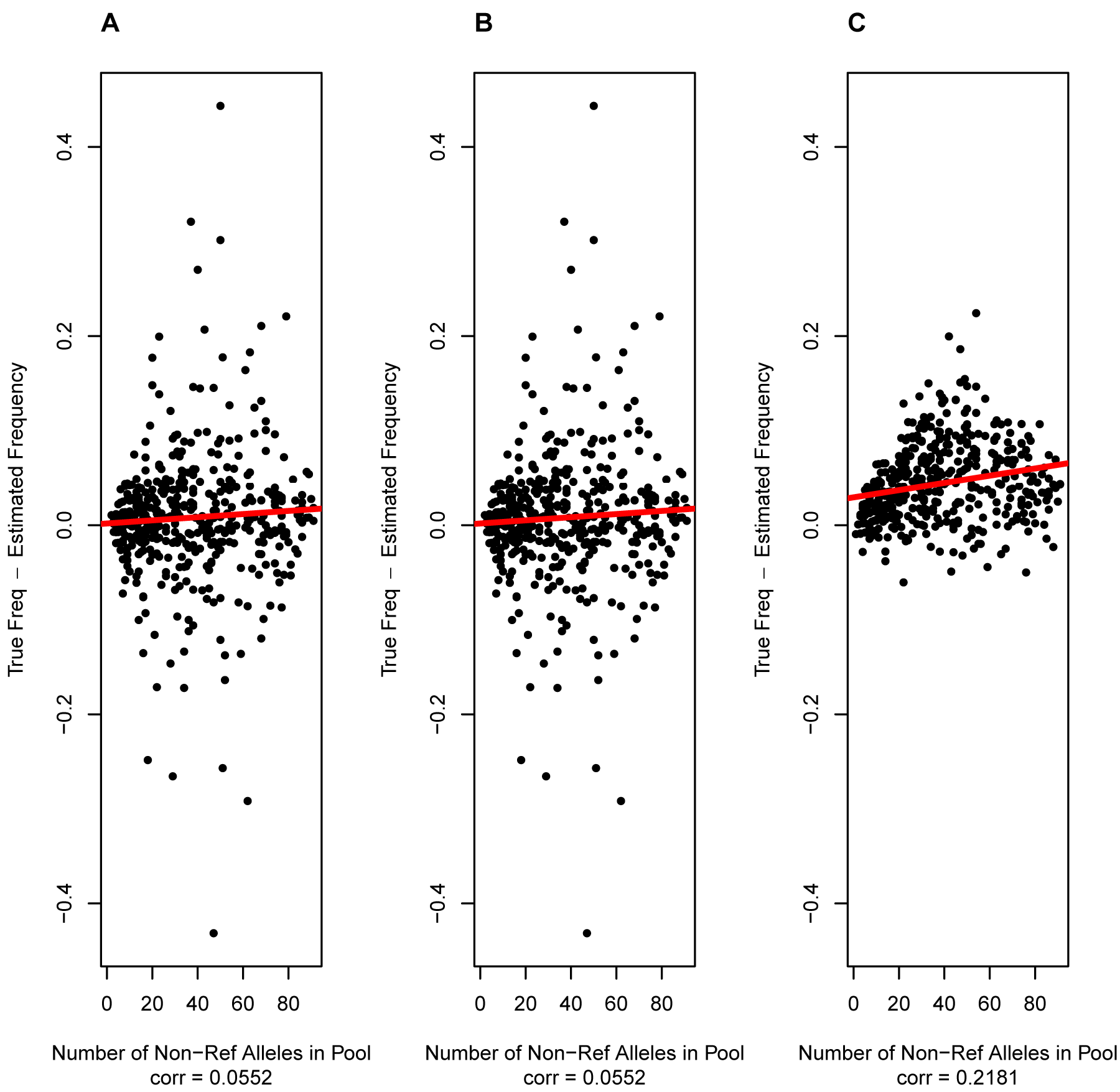

Supplement: Figure S40 — HapMap/58BC intersection allele frequency estimation accuracy as a function of allele count in the Pool of 50 before duplicate removal. This figure is a scatter plot of the accuracy of the allele frequency estimates from the sequencing compared to the number of variant alleles at HapMap/58BC intersection true positive variants in the Pool of 50 individuals for: (A) PCR, (B) aHC and (C) sHC enrichment. The accuracy of the estimates are calculated as the frequency calculated from the HapMap genotypes minus the frequency estimated from the sequencing data. The y-axis is the accuracy value and the x-axis is the number of variant alleles in the pool. The red line is the least squares fit of the model , and the corr is the Pearson's correlation coefficient between the accuracy and read depth. (TIF) [file pone.0026279.s040.tif]
