# Supplementary material for: An Evaluation of Different Target Enrichment Methods in Pooled Sequencing Designs for Complex Disease Association Studies
Source: PLoS One. 2011 Nov 1;6(11):e26279. doi: 10.1371/journal.pone.0026279 (PMC3206031; doi:10.1371/journal.pone.0026279)
Supplement: Table S1 — Non-indexed pool designs. This table details the HapMap and 1958BC sample composition of the non-indexed pools of size 2, 10, 20 and 50. The table also details the number of HapMap individuals in each pool that were sequenced in pilot 1 of the 1KG project. (PDF) [file pone.0026279.s041.pdf]

Pool of	Number HapMap	Number 1958 BC	Number Seq 1000 genome
1	1	0	1
2	2	0	2
10	10	0	9
20	20	0	17
50	31	19	22

Table S1: Non-indexed pool designs. This table details the HapMap and 1958BC sample composition of the non-indexed pools of size 2, 10, 20 and 50. The table also details the number of HapMap individuals in each pool that were sequenced in pilot 1 of the 1KG project.
