# Supplementary material for: An Evaluation of Different Target Enrichment Methods in Pooled Sequencing Designs for Complex Disease Association Studies
Source: PLoS One. 2011 Nov 1;6(11):e26279. doi: 10.1371/journal.pone.0026279 (PMC3206031; doi:10.1371/journal.pone.0026279)
Supplement: Table S2 — Pool sequencing designs. This table details the number of lanes sequenced per pool, the read lengths generated per lane, and whether the pool had technical replicates performed. (PDF) [file pone.0026279.s042.pdf]

Pool of	Number Lanes	Read Length(s)	Performed in Duplicate
1	1	54	No
2	1	37	No
10	2	37,54	No
20	3	37,54	Yes
50	7	37,54	No

Table S2: Pool sequencing designs. This table details the number of lanes sequenced per pool, the read lengths generated per lane, and whether the pool had technical replicates performed.
