# Supplementary material for: An Evaluation of Different Target Enrichment Methods in Pooled Sequencing Designs for Complex Disease Association Studies
Source: PLoS One. 2011 Nov 1;6(11):e26279. doi: 10.1371/journal.pone.0026279 (PMC3206031; doi:10.1371/journal.pone.0026279)
Supplement: Table S3 — Target regions for enrichment. These 6 genomic regions were selected for sequence enrichment on the basis of preliminary rare variant association to Type 2 Diabetes. The target regions include 50 Kb upstream and down stream of the translation start and stop sites for each gene, and include both protein coding (COD) and non-coding (NON-COD) sequence. (PDF) [file pone.0026279.s043.pdf]

Gene	Chr	NON-COD BP	COD BP	Total BP
BMPR1B	4	498,965	1,509	500,474
HAPLN1	5	178,890	1,065	179,955
EFHA2	8	193,807	1,593	195,400
CPN1	10	138,201	1,377	139,578
GALNTL4	11	449,306	1,824	451,130
KCNJ15	21	143,955	1,128	145,083
Total Mb		1,603,124 (99.47%)[a]	8,496 (0.52%)[a]	1,611,614

a: percentage of total target region

Table S3: Target regions for enrichment. These 6 genomic regions were selected for sequence enrichment on the basis of preliminary rare variant association to Type 2 Diabetes. The target regions include 50Kb upstream and down stream of the translation start and stop sites for each gene, and include both protein coding (COD) and non-coding (NON-COD) sequence.
