# Supplementary material for: An Evaluation of Different Target Enrichment Methods in Pooled Sequencing Designs for Complex Disease Association Studies
Source: PLoS One. 2011 Nov 1;6(11):e26279. doi: 10.1371/journal.pone.0026279 (PMC3206031; doi:10.1371/journal.pone.0026279)
Supplement: Table S4 — Target sequence enrichment success after duplicate removal. For each pool and sequence enrichment method this table details the total number of reads generated for the pool, the estimated percentage of duplicate reads, the percentage of total reads mapped to the reference genome after duplicate removal, the percentage of total reads mapped to the target regions after duplicate removal, and the percentage of mapped reads that mapped to the target regions with mapping quality 20 after duplicate removal. The total number of reads for a pool is calculated from the fastq file(s) generated for each lane of sequencing. The percentage of reads mapped to the reference is calculated from the BAM file generated from merging all the Maq map files for each lane for a pool. The percentage of reads mapped to the target regions is calculated as the number of reads with at least one base overlapping a target region divided by the total number of reads. The percentage of reads mapped to the target regions with a mapping quality score Q20 is calculated as the number of reads with at least one base overlapping a target region with mapping Q20 divided by the total number of reads. (PDF) [file pone.0026279.s044.pdf]

Pool of	Number Lanes	Total Number Reads	Estimated % Duplicates[a]	% Reads Mapped to Reference[b]	% Reads Mapped to Target[b]	% Reads Mapped to Target w/ $\geq$ Q20[c]
1 PCR	1	44,232,852	68.71	18.42	16.27	14.72
1 aHC	1	61,487,334	15.01	83.36	18.40	18.18
1 sHC	1	35,813,898	25.63	74.07	33.37	32.83
2 PCR	1	30,843,770	15.88	86.34	74.78	68.83
2 aHC	1	58,352,664	19.47	78.13	10.64	10.49
2 sHC	1	29,554,192	20.15	79.05	36.55	36.00
10 PCR	2	55,278,922	31.58	63.61	53.38	47.91
10 aHC	2	90,319,688	13.15	87.09	16.35	15.89
10 sHC	2	85,783,964	41.46	58.95	26.10	25.54
20 PCR	3	121,378,560	48.44	53.88	46.88	42.24
20 aHC	3	103,231,280	26.96	73.52	24.44	23.88
20 sHC	3	111,444,476	46.70	53.73	22.20	21.70
50 PCR	7	132,547,082	39.66	63.83	40.43	37.68
50 aHC	7	251,257,124	50.12	51.47	10.55	10.24
50 sHC	7	295,115,044	71.65	30.38	12.37	11.85

a: Estimate calculated by Picard's MarkDuplicates on original mapped bam

b: Calculated by samtools view -c on bam file after duplicates removed

c: Calculated by samtoools veiw -c -q 20 on bam file after duplicates removed

Table S4: Target sequence enrichment success after duplicate removal. For each pool and sequence enrichment method this table details the total number of reads generated for the pool, the estimated percentage of duplicate reads, the percentage of total reads mapped to the reference genome after duplicate removal, the percentage of total reads mapped to the target regions after duplicate removal, and the percentage of mapped reads that mapped to the target regions with mapping quality ≥ 20 after duplicate removal. The total number of reads for a pool is calculated from the fastq file(s) generated for each lane of sequencing. The percentage of reads mapped to the reference is calculated from the BAM file generated from merging all the Maq map files for each lane for a pool. The percentage of reads mapped to the target regions is calculated as the number of reads with at least one base overlapping a target region divided by the total number of reads. The percentage of reads mapped to the target regions with a mapping quality score $\geq$ Q20 is calculated as the number of reads with at least one base overlapping a target region with mapping Q ≥ 20 divided by the total number of reads.
