# Supplementary material for: An Evaluation of Different Target Enrichment Methods in Pooled Sequencing Designs for Complex Disease Association Studies
Source: PLoS One. 2011 Nov 1;6(11):e26279. doi: 10.1371/journal.pone.0026279 (PMC3206031; doi:10.1371/journal.pone.0026279)
Supplement: Table S5 — Enrichment success for technical replicates before duplicate removal. For each technical replicate of the Pool of 20 this table details the total number of reads generated for the pool, the percentage of total reads mapped to the reference genome, the percentage of total reads mapped to the target regions, the percentage of mapped reads that mapped to the target regions, and the median read depth of the target regions. The total number of reads for a pool is calculated from the fastq file(s) generated for each lane of sequencing. The percentage of reads mapped to the reference is calculated from the BAM file generated from merging all the Maq map files for each lane for a pool. The percentage of reads mapped to the target regions is calculated as the number of reads with at least one base overlapping a target region divided by the total number of reads. The percentage of mapped reads mapped to the target is calculated as the number of reads with at least one base overlapping a target region divided by the total number or reads mapped in the BAM file. (PDF) [file pone.0026279.s045.pdf]

Pool of	Total Number Reads	% Reads Mapped to Reference[a]	% Reads Mapped to Target[a]	% of Mapped Reads Mapped to Target[a]	Median Target Coverage
20 Rep 1 PCR[b]	121,378,560	89.33	80.88	90.54	1429
20 Rep 2 PCR	58,876,300	89.05	79.28	89.03	703
20 Rep 1 aHC[b]	103,231,280	97.24	34.05	35.02	736
20 Rep 2 aHC	116,495,340	95.97	20.99	21.87	489

a: Calculated by samtools view -c on bam files before duplicates removed

b: replicate shown in all main analyses

Table S5: Enrichment success for technical replicates before duplicate removal. For each technical replicate of the Pool of 20 this table details the total number of reads generated for the pool, the percentage of total reads mapped to the reference genome, the percentage of total reads mapped to the target regions, the percentage of mapped reads that mapped to the target regions, and the median read depth of the target regions. The total number of reads for a pool is calculated from the fastq file(s) generated for each lane of sequencing. The percentage of reads mapped to the reference is calculated from the BAM file generated from merging all the Maq map files for each lane for a pool. The percentage of reads mapped to the target regions is calculated as the number of reads with at least one base overlapping a target region divided by the total number of reads. The percentage of mapped reads mapped to the target is calculated as the number of reads with at least one base overlapping a target region divided by the total number or reads mapped in the BAM file.
