# Supplementary material for: An Evaluation of Different Target Enrichment Methods in Pooled Sequencing Designs for Complex Disease Association Studies
Source: PLoS One. 2011 Nov 1;6(11):e26279. doi: 10.1371/journal.pone.0026279 (PMC3206031; doi:10.1371/journal.pone.0026279)
Supplement: Table S7 — Coverage of the target region before duplicate removal. For each pool and enrichment method this table shows the mean, median and standard deviation of target coverage before duplicate removal. The mean coverage is calculated by summing the read depth for each target base and dividing by the total length of the target regions. The median and standard deviation are calculated from the distribution of read depths for target bases. (PDF) [file pone.0026279.s047.pdf]

Pool of	Mean Target Coverage[a]	Median Target Coverage	Standard Deviation[a]
1 PCR	229	36	481
1 aHC	376	369	221
1 sHC	400	322	304
2 PCR	518	397	448
2 aHC	205	198	124
2 sHC	362	304	258
10 PCR	761	657	521
10 aHC	426	422	250
10 sHC	739	633	515
20 PCR	1537	1429	845
20 aHC	761	736	473
20 sHC	811	710	542
50 PCR	1731	1659	919
50 aHC	837	738	635
50 sHC	1130	1027	710

a: rounded to the nearest whole read count

Table S7: Coverage of the target region before duplicate removal. For each pool and enrichment method this table shows the mean, median and standard deviation of target coverage before duplicate removal. The mean coverage is calculated by summing the read depth for each target base and dividing by the total length of the target regions. The median and standard deviation are calculated from the distribution of read depths for target bases.
