# Supplementary material for: An Evaluation of Different Target Enrichment Methods in Pooled Sequencing Designs for Complex Disease Association Studies
Source: PLoS One. 2011 Nov 1;6(11):e26279. doi: 10.1371/journal.pone.0026279 (PMC3206031; doi:10.1371/journal.pone.0026279)
Supplement: Table S8 — Coverage of the target region after duplicate removal. For each pool and enrichment method this table shows the mean, median and standard deviation of target coverage after duplicate removal. The mean coverage is calculated by summing the read depth for each target base and dividing by the total length of the target regions. The median and standard deviation are calculated from the distribution of read depths for target bases. (PDF) [file pone.0026279.s048.pdf]

Pool of	Mean Target Coverage[a]	Median Target Coverage	Standard Deviation[a]
1 PCR	202	35	410
1 aHC	368	361	216
1 sHC	381	312	282
2 PCR	506	390	434
2 aHC	200	193	121
2 sHC	350	297	244
10 PCR	736	640	495
10 aHC	420	416	245
10 sHC	685	599	461
20 PCR	1426	1331	773
20 aHC	735	715	451
20 sHC	744	664	480
50 PCR	1573	1521	806
50 aHC	735	666	536
50 sHC	939	874	565

a: rounded to the nearest whole read count

Table S8: Coverage of the target region after duplicate removal. For each pool and enrichment method this table shows the mean, median and standard deviation of target coverage after duplicate removal. The mean coverage is calculated by summing the read depth for each target base and dividing by the total length of the target regions. The median and standard deviation are calculated from the distribution of read depths for target bases.
