# Supplementary material for: An Evaluation of Different Target Enrichment Methods in Pooled Sequencing Designs for Complex Disease Association Studies
Source: PLoS One. 2011 Nov 1;6(11):e26279. doi: 10.1371/journal.pone.0026279 (PMC3206031; doi:10.1371/journal.pone.0026279)
Supplement: Table S9 — Percentage of target region reads that mapped to the coding vs non-coding regions before duplicate removal. This table gives the percentage of target reads that mapped to the coding (COD) and non-coding (NON-COD) regions before duplicate removal. This table also gives the median read depth in the coding and non-coding target regions (PDF) [file pone.0026279.s049.pdf]

Pool of	% Target Reads Mapped to COD*	Median COD Coverage	% Target Reads Mapped to NON-COD*	Median NON-COD Coverage
1 PCR	0.34	8	99.92	36
1 aHC	0.86	484	99.51	368
1 sHC	1.08	603	99.35	321
2 PCR	0.59	449	99.60	397
2 aHC	0.81	244	99.56	197
2 sHC	1.03	529	99.38	303
10 PCR	0.63	687	99.61	656
10 aHC	0.80	492	99.52	421
10 sHC	1.09	1116	99.34	631
20 PCR	0.55	1102	99.70	1431
20 aHC	0.91	1020	99.44	735
20 sHC	1.07	1197	99.36	708
50 PCR	0.66	1481	99.65	1660
50 aHC	1.12	1287	99.31	735
50 sHC	1.07	1598	99.36	1024

*: Percentage of reads with at least one base overlapping region of interest;
A single read can overlap both coding and non-coding regions therefore
the percentages won't sum to 100%.

Table S9: Percentage of target region reads that mapped to the coding vs non-coding regions before duplicate removal. This table gives the percentage of target reads that mapped to the coding (COD) and non-coding (NON-COD) regions before duplicate removal. This table also gives the median read depth in the coding and non-coding target regions
