# Supplementary material for: An Evaluation of Different Target Enrichment Methods in Pooled Sequencing Designs for Complex Disease Association Studies
Source: PLoS One. 2011 Nov 1;6(11):e26279. doi: 10.1371/journal.pone.0026279 (PMC3206031; doi:10.1371/journal.pone.0026279)
Supplement: Table S10 — Percentage of target region reads that mapped to the coding vs non-coding regions after duplicate removal. This table gives the percentage of target reads that mapped to the coding (COD) and non-coding (NON-COD) regions after duplicate removal. This table also gives the median read depth in the coding and non-coding target regions. (PDF) [file pone.0026279.s050.pdf]

Pool of	% Target Reads Mapped to COD*	Median COD Coverage	% Target Reads Mapped to NON-COD*	Median NON-COD Coverage
1 PCR	0.37	8	99.9	35
1 aHC	0.88	476	99.5	360
1 sHC	1.03	574	99.4	311
2 PCR	0.60	440	99.6	390
2 aHC	0.83	240	99.5	192
2 sHC	0.99	509	99.4	296
10 PCR	0.64	669	99.6	639
10 aHC	0.80	486	99.5	416
10 sHC	0.99	1022	99.4	597
20 PCR	0.56	1034	99.7	1332
20 aHC	0.88	983	99.5	714
20 sHC	0.97	1085	99.4	663
50 PCR	0.65	1365	99.6	1522
50 aHC	1.02	1102	99.4	663
50 sHC	0.93	1308	99.5	872

*: Percentage of reads with at least one base overlapping region of interest; A single read can overlap both coding and non-coding regions therefore the percentages won't sum to 100%.

Table S10: Percentage of target region reads that mapped to the coding vs non-coding regions after duplicate removal. This table gives the percentage of target reads that mapped to the coding (COD) and non-coding (NON-COD) regions after duplicate removal. This table also gives the median read depth in the coding and non-coding target regions.
