# Supplementary material for: An Evaluation of Different Target Enrichment Methods in Pooled Sequencing Designs for Complex Disease Association Studies
Source: PLoS One. 2011 Nov 1;6(11):e26279. doi: 10.1371/journal.pone.0026279 (PMC3206031; doi:10.1371/journal.pone.0026279)
Supplement: Table S11 — Sequence characteristics of non-coding vs coding target regions. An analysis of the sequence characteristics of the target coding (COD) and non-coding (NON-COD) regions including the repeat content as analyzed by RepeatMasker open 3.2.9. (PDF) [file pone.0026279.s051.pdf]

Sequence Feature	NON-COD % of Seq	COD % of Seq
GC content	41.34	50.72
SINEs	12.21	0
LINEs	15.59	0
LTR elements	8.35	0
DNA elements	3.56	0
Simple Repeats	0.76	0
Low Complexity	0.62	0.27
Total Repeat	41.09	0.27

Table S11: Sequence characteristics of non-coding vs coding target regions An analysis of the sequence characteristics of the target coding (COD) and non-coding (NON-COD) regions including the repeat content as analyzed by RepeatMasker open 3.2.9.
