# Supplementary material for: An Evaluation of Different Target Enrichment Methods in Pooled Sequencing Designs for Complex Disease Association Studies
Source: PLoS One. 2011 Nov 1;6(11):e26279. doi: 10.1371/journal.pone.0026279 (PMC3206031; doi:10.1371/journal.pone.0026279)
Supplement: Table S12 — Total number of variants called by pool and enrichment technique after duplicate removal. For each pool size and sequence enrichment method this table details the total number of variants called from the sequencing data. (PDF) [file pone.0026279.s052.pdf]

	Pool of 1	Pool of 2	Pool of 10	Pool of 20	Pool of 50
**PCR**	616	3102	6358	9316	16840
**aHC**	2272	2563	5874	8883	9469
**sHC**	2256	3270	6783	7888	9911

Table S12: Total number of variants called by pool and enrichment technique after duplicate removal. For each pool size and sequence enrichment method this table details the total number of variants called from the sequencing data.
