# Supplementary material for: An Evaluation of Different Target Enrichment Methods in Pooled Sequencing Designs for Complex Disease Association Studies
Source: PLoS One. 2011 Nov 1;6(11):e26279. doi: 10.1371/journal.pone.0026279 (PMC3206031; doi:10.1371/journal.pone.0026279)
Supplement: Table S13 — Total number of variants called by pool and enrichment technique before duplicate removal. For each pool size and sequence enrichment method this table details the total number of variants called from the sequencing data. (PDF) [file pone.0026279.s053.pdf]

	Pool of 1	Pool of 10	Pool of 50
**PCR**	1938	8100	19541
**sHC**	2742	7478	13923

Table S13: Total number of variants called by pool and enrichment technique before duplicate removal. For each pool size and sequence enrichment method this table details the total number of variants called from the sequencing data.
