# Supplementary material for: An Evaluation of Different Target Enrichment Methods in Pooled Sequencing Designs for Complex Disease Association Studies
Source: PLoS One. 2011 Nov 1;6(11):e26279. doi: 10.1371/journal.pone.0026279 (PMC3206031; doi:10.1371/journal.pone.0026279)
Supplement: Table S14 — dbSNP129 overlap after duplicate removal. This table contains the percentage of called variants for each pool and enrichment method that are present in the non-redundant dbSNP129. (PDF) [file pone.0026279.s054.pdf]

	Pool of 1	Pool of 2	Pool of 10	Pool of 20	Pool of 50
PCR	83.28	78.37	60.55	46.74	27.70
aHC	86.53	84.00	63.93	46.95	43.94
sHC	88.16	82.39	62.07	55.81	47.11

Table S14: dbSNP129 overlap after duplicate removal. This table contains the percentage of called variants for each pool and enrichment method that are present in the non-redundant dbSNP129.
