# Supplementary material for: An Evaluation of Different Target Enrichment Methods in Pooled Sequencing Designs for Complex Disease Association Studies
Source: PLoS One. 2011 Nov 1;6(11):e26279. doi: 10.1371/journal.pone.0026279 (PMC3206031; doi:10.1371/journal.pone.0026279)
Supplement: Table S15 — dbSNP129 overlap before duplicate removal. This table contains the percentage of called variants for each pool and enrichment method that are present in the non-redundant dbSNP129. (PDF) [file pone.0026279.s055.pdf]

	Pool of 1	Pool of 10	Pool of 50
PCR	70.95	50.88	24.65
sHC	83.92	59.69	35.87

Table S15: dbSNP129 overlap before duplicate removal. This table contains the percentage of called variants for each pool and enrichment method that are present in the non-redundant dbSNP129.
