# Supplementary material for: An Evaluation of Different Target Enrichment Methods in Pooled Sequencing Designs for Complex Disease Association Studies
Source: PLoS One. 2011 Nov 1;6(11):e26279. doi: 10.1371/journal.pone.0026279 (PMC3206031; doi:10.1371/journal.pone.0026279)
Supplement: Table S16 — HapMap variation detection sensitivity before duplicate removal. This table contains the percentage of the known HapMap variants with at least one non-reference allele in the pool that each pool and enrichment method discovered (true positives). The false negative rate is 100 minus this value. (PDF) [file pone.0026279.s056.pdf]

	Pool of 1	Pool of 10	Pool of 50
PCR	69.23	95.20	96.22
sHC	99.62	99.50	98.18

a: number of non-reference HapMap variants in pool

Table S16: HapMap variation detection sensitivity before duplicate removal. This table contains the percentage of the known HapMap variants with at least one non-reference allele in the pool that each pool and enrichment method discovered (true positives). The false negative rate is 100 minus this value.
