# Supplementary material for: An Evaluation of Different Target Enrichment Methods in Pooled Sequencing Designs for Complex Disease Association Studies
Source: PLoS One. 2011 Nov 1;6(11):e26279. doi: 10.1371/journal.pone.0026279 (PMC3206031; doi:10.1371/journal.pone.0026279)
Supplement: Table S17 — HapMap variation detection specificity before duplicate removal. This table contains the percentage of the known HapMap variants with no non-reference alleles and no missing genotypes in the pool that each pool and enrichment method correctly didn't call as a variant (true negatives). The false positive rate is 100 minus this value. (PDF) [file pone.0026279.s057.pdf]

	Pool of 1 (1722)[a]	Pool of 10 (683)[a]
PCR	99.77	96.93
sHC	98.84	97.22

a: number of reference HapMap variants in pool

Table S17: HapMap variation detection specificity before duplicate removal. This table contains the percentage of the known HapMap variants with no non-reference alleles and no missing genotypes in the pool that each pool and enrichment method correctly didn't call as a variant (true negatives). The false positive rate is 100 minus this value.
