# Supplementary material for: An Evaluation of Different Target Enrichment Methods in Pooled Sequencing Designs for Complex Disease Association Studies
Source: PLoS One. 2011 Nov 1;6(11):e26279. doi: 10.1371/journal.pone.0026279 (PMC3206031; doi:10.1371/journal.pone.0026279)
Supplement: Table S18 — 1KG support for HapMap false positive loci before duplicate removal. This table contains the number of loci considered false positives based on HapMap data that are present in 1KG and the percentage of these overlapping loci that the 1KG data supports the presence of non-reference alleles in the pool. (PDF) [file pone.0026279.s058.pdf]

	Pool of 1	Pool of 10
PCR	4(75%)	18(94.44%)
sHC	19(94.74%)	16(100%)

Table S18: 1KG support for HapMap false positive loci before duplicate removal. This table contains the number of loci considered false positives based on HapMap data that are present in 1KG and the percentage of these overlapping loci that the 1KG data supports the presence of non-reference alleles in the pool.
