# Supplementary material for: An Evaluation of Different Target Enrichment Methods in Pooled Sequencing Designs for Complex Disease Association Studies
Source: PLoS One. 2011 Nov 1;6(11):e26279. doi: 10.1371/journal.pone.0026279 (PMC3206031; doi:10.1371/journal.pone.0026279)
Supplement: Table S20 — 1KG variation detection sensitivity before duplicate removal. This table contains the percentage of the known 1KG variants with at least one non-reference allele in the pool that each pool and enrichment method discovered (true positives). The false negative rate is 100 minus this value. (PDF) [file pone.0026279.s060.pdf]

	Pool of 1 (2197)[a]	Pool of 10 (4408)[a]	Pool of 50 (5118)[a]
PCR	60.49	90.06	92.73
sHC	97.81	97.16	96.64

a: number of non-reference 1KG variants in pool

Table S20: 1KG variation detection sensitivity before duplicate removal. This table contains the percentage of the known 1KG variants with at least one non-reference allele in the pool that each pool and enrichment method discovered (true positives). The false negative rate is 100 minus this value.
