# Supplementary material for: An Evaluation of Different Target Enrichment Methods in Pooled Sequencing Designs for Complex Disease Association Studies
Source: PLoS One. 2011 Nov 1;6(11):e26279. doi: 10.1371/journal.pone.0026279 (PMC3206031; doi:10.1371/journal.pone.0026279)
Supplement: Table S21 — Total known HapMap/1KG variation detection sensitivity after duplicate removal. This table contains the percentage of all the known variants with at least one non-reference allele in the pool that each pool and enrichment method discovered (true positives). The false negative rate is 100 minus this value. For individuals that have both 1KG and HapMap data, if a locus occurred in both data sets the HapMap genotype was selected. If a locus occurred in both data sets and an individual's HapMap genotype was missing but called in 1KG, the 1KG genotype was used. (PDF) [file pone.0026279.s061.pdf]

	Pool of 1 (2205)^{a,b}	Pool of 2 (2992)^{a,b}	Pool of 10 (4481)^{a,b}	Pool of 20 (4957)^{a,b}	Pool of 50 (5192)^{a,c}
PCR	23.08	80.72	86.30	90.54	90.85
aHC	90.16	74.53	89.18	91.29	86.23
sHC	88.21	89.44	94.38	93.32	92.35

a: number of non-reference variants in pool

b: calculated as the union of HapMap and 1KG genotypes

c: calculated as the union of HapMap, 1KG, and 58BC Illumina genotypes

Table S21: Total known HapMap/1KG variation detection sensitivity after duplicate removal. This table contains the percentage of all the known variants with at least one non-reference allele in the pool that each pool and enrichment method discovered (true positives). The false negative rate is 100 minus this value. For individuals that have both 1KG and HapMap data, if a locus occurred in both data sets the HapMap genotype was selected. If a locus occurred in both data sets and an individual's HapMap genotype was missing but called in 1KG, the 1KG genotype was used.
