# Supplementary material for: An Evaluation of Different Target Enrichment Methods in Pooled Sequencing Designs for Complex Disease Association Studies
Source: PLoS One. 2011 Nov 1;6(11):e26279. doi: 10.1371/journal.pone.0026279 (PMC3206031; doi:10.1371/journal.pone.0026279)
Supplement: Table S23 — HapMap singleton detection sensitivity after duplicate removal. This table illustrates the ability of the sequencing based variant calling to identify variants where the HapMap genotypes have a single non-reference or reference base. The only loci analyzed here are those where there are no missing genotypes for pooled individuals. (PDF) [file pone.0026279.s063.pdf]

	Pool of 2 849[a]	Pool of 10 94[a]	Pool of 20 50[a]
**PCR**	86.1	73.4	84
**aHC**	84.6	86.2	86
**sHC**	94.6	97.9	86

a: The number of singletons in the pool where there were no missing genotypes

Table S23: HapMap singleton detection sensitivity after duplicate removal. This table illustrates the ability of the sequencing based variant calling to identify variants where the HapMap genotypes have a single non-reference or reference base. The only loci analyzed here are those where there are no missing genotypes for pooled individuals.
