# Supplementary material for: An Evaluation of Different Target Enrichment Methods in Pooled Sequencing Designs for Complex Disease Association Studies
Source: PLoS One. 2011 Nov 1;6(11):e26279. doi: 10.1371/journal.pone.0026279 (PMC3206031; doi:10.1371/journal.pone.0026279)
Supplement: Table S24 — Variation detection sensitivity as pool size grows after duplicate removal. This table shows the percentage of the variants called in the pool of 1 individual that are also called as variants in the larger pool sizes. The individual in the pool of 1 was also in each subsequent larger pool, therefore all variants called in the pool of 1 should also be found in all subsequent pools. (PDF) [file pone.0026279.s064.pdf]

	Pool of 2	Pool of 10	Pool of 20	Pool of 50
PCR (616)[a]	65.75	84.25	97.40	96.92
aHC (2272)[a]	77.33	97.40	99.34	97.71
sHC (2256)[a]	97.03	99.73	99.02	98.85

a: The number of variants called in Pool of 1

Table S24: Variation detection sensitivity as pool size grows after duplicate removal. This table shows the percentage of the variants called in the pool of 1 individual that are also called as variants in the larger pool sizes. The individual in the pool of 1 was also in each subsequent larger pool, therefore all variants called in the pool of 1 should also be found in all subsequent pools.
