# Supplementary material for: An Evaluation of Different Target Enrichment Methods in Pooled Sequencing Designs for Complex Disease Association Studies
Source: PLoS One. 2011 Nov 1;6(11):e26279. doi: 10.1371/journal.pone.0026279 (PMC3206031; doi:10.1371/journal.pone.0026279)
Supplement: Table S25 — Variation detection sensitivity as pool size grows before duplicate removal. This table shows the percentage of the variants called in the pool of 1 individual that are also called as variants in the larger pool sizes. The individual in the pool of 1 was also in each subsequent larger pool, therefore all variants called in the pool of 1 should also be found in all subsequent pools. (PDF) [file pone.0026279.s065.pdf]

| | Pool of 10 | Pool of 50 |
|---|---|---|
| PCR (1938)[a] | 80.08 | 85.81 |
| sHC (2742)[a] | 98.72 | 98.80 |

a: The number of variants called in Pool of 1

**Table S25: Variation detection sensitivity as pool size grows before duplicate removal.** This table shows the percentage of the variants called in the pool of 1 individual that are also called as variants in the larger pool sizes. The individual in the pool of 1 was also in each subsequent larger pool, therefore all variants called in the pool of 1 should also be found in all subsequent pools.
