# Supplementary material for: An Evaluation of Different Target Enrichment Methods in Pooled Sequencing Designs for Complex Disease Association Studies
Source: PLoS One. 2011 Nov 1;6(11):e26279. doi: 10.1371/journal.pone.0026279 (PMC3206031; doi:10.1371/journal.pone.0026279)
Supplement: Table S27 — Percent of called HapMap variants with correctly, under, and over estimated non-reference allele frequencies before duplicate removal. For the pools of 10, 20 and 50 individuals and each enrichment method this table details the percent of true positive variants that the non-reference allele frequency was correctly, under, or over estimated by the sequencing based variant caller relative to the reference genotypes. (PDF) [file pone.0026279.s067.pdf]

Pool of-Enrich	% Correctly Estimated	% Under Estimated	% Over Estimated	Avg. Absolute Freq. Diff
10-PCR[a]	9.8	58.2	32.0	0.0798
10-sHC[a]	18.3	70.2	11.5	0.0504
50-PCR[b]	0	58.0	42.0	0.0441
50-sHC[b]	0	88.4	11.6	0.0473

a: HapMap loci with 0 missing genotypes

b: Intersection of HapMap and 58C Illumina genotypes, allowing 2 missing genotypes

Table S27: Percent of called HapMap variants with correctly, under, and over estimated non-reference allele frequencies before duplicate removal. For the pools of 10, 20 and 50 individuals and each enrichment method this table details the percent of true positive variants that the non-reference allele frequency was correctly, under, or over estimated by the sequencing based variant caller relative to the reference genotypes.
