# Supplementary material for: An Evaluation of Different Target Enrichment Methods in Pooled Sequencing Designs for Complex Disease Association Studies
Source: PLoS One. 2011 Nov 1;6(11):e26279. doi: 10.1371/journal.pone.0026279 (PMC3206031; doi:10.1371/journal.pone.0026279)
Supplement: Table S28 — Pool of 20 technical replicates dbSNP overlap and HapMap/1KG sensitivity after duplicate removal. This table contains the percentage of the called variants in dbSNP129, and the percentage of known HapMap/1KG variants with at least one non-reference allele in the pool that each replicate discovered (true positives). The false negative rate is 100 minus this value. (PDF) [file pone.0026279.s068.pdf]

	% variants in dbSNP129	HapMap (2067)^a	1KG (4908)^a
PCR Rep 1	46.74	96.27	90.67
PCR Rep 2	40.34	92.45	85.88
aHC Rep 1	46.95	97.82	91.46
aHC Rep 2	41.64	95.21	85.64

a: number of non-reference variants in Pool

Table S 28: Pool of 20 technical replicates dbSNP overlap and HapMap/1KG sensitivity after duplicate removal. This table contains the percentage of the called variants in dbSNP129, and the percentage of known HapMap/1KG variants with at least one non-reference allele in the pool that each replicate discovered (true positives). The false negative rate is 100 minus this value.
