# Supplementary material for: An Evaluation of Different Target Enrichment Methods in Pooled Sequencing Designs for Complex Disease Association Studies
Source: PLoS One. 2011 Nov 1;6(11):e26279. doi: 10.1371/journal.pone.0026279 (PMC3206031; doi:10.1371/journal.pone.0026279)
Supplement: Table S29 — Pool of 20 technical replicate variant overlap after duplicate removal. For the PCR and aHC technical replicates for the pool of 20 this table details the total number of variants called for each replicate, the number of variants called by both replicates, the percent overlap of the called variants in the replicates, the average absolute difference in non-reference allele frequency for the overlapping variants, and the Pearson's correlation coefficient for the non-reference allele frequency estimates between the replicates. The average absolute difference is calculated as the sum of the absolute value of the difference in non-reference allele frequency, divided by the total number of sites. (PDF) [file pone.0026279.s069.pdf]

Enrichment Technique	Replicate 1 variants	Replicate 2 variants	Num variants Overlap	% Overlap[a]	Freq Diff[b]	Corr. Coeff.[c]
PCR	9316	10185	7233	59	0.027	0.9859
aHC	8883	9214	6713	59	0.021	0.9932

a: The number overlapping/total number of unique variants

b: The avg. absolute frequency diff of non-ref allele of overlap

c: The correlation coefficient of non-ref allele frequency of overlap

Table S29: Pool of 20 technical replicate variant overlap after duplicate removal. For the PCR and aHC technical replicates for the pool of 20 this table details the total number of variants called for each replicate, the number of variants called by both replicates, the percent overlap of the called variants in the replicates, the average absolute difference in non-reference allele frequency for the overlapping variants, and the Pearson's correlation coefficient for the non-reference allele frequency estimates between the replicates. The average absolute difference is calculated as the sum of the absolute value of the difference in non-reference allele frequency, divided by the total number of sites.
